# Type 2 diabetes epidemic in East Asia: a 35–year systematic trend analysis

**DOI:** 10.18632/oncotarget.22961

**Published:** 2017-12-06

**Authors:** Huiping Yuan, Xinghui Li, Gang Wan, Liang Sun, Xiaoquan Zhu, Fugang Che, Ze Yang

**Affiliations:** ^1^ The MOH Key Laboratory of Geriatrics, Beijing Hospital, National Center of Gerontology, Beijing, P.R. China; ^2^ College of Public Health, Shaanxi University of Chinese Medicine, Xianyang, P.R. China; ^3^ Statistical Room, Beijing Ditan Hospital Capital University, Beijing, P.R. China

**Keywords:** type 2 diabetes, prevalence, epidemic features, risk factors, temporal variation trend

## Abstract

Facing the challenge of effective prevention type 2 diabetes (T2DM) in China (as part of global health) requires knowledge about both the temporal trend and risk factors variation in T2DM. We searched the PubMed, CNKI, WANFANG, and International Diabetes Federation (IDF) databases for data on the prevalence of T2DM/ IGT (impaired glucose tolerance) published from January 1, 1980 to December 31, 2014 in China, Japan and Korea. The prevalence of T2DM was estimated with 95% confidence intervals (CIs) using random–effects meta–analysis. T2DM prevalence trend in the next 10 years was estimated by using a time series regression model based on the 35 years of data. The 621 articles covered 11.8 million Chinese people, 1.64 million Japanese, and 37.69 million Koreans. The aggregate prevalence of T2DM in China has increased sharply from 1.3% in 1980–1989 to 4.5% in 1990–1999, 6.8% 2000–2009, and 8.7% in 2010–2014. We estimated that by 2025, T2DM prevalence will have grown to 12.5%. Central obesity is the largest preventable cause of T2DM. We also found that female having a very high BMI (body mass index, ≥28 kg/m2) and being an older (≥50 years old) female are next–highest risk factors for T2DM compared with male. Consistent with the patterns characterized for China, T2DM prevalence in Japan increased with aging, and men were more likely to develop T2DM. It was the same as Korea. In the Far East, especially in China, T2DM prevalence will continue to increase until 2025. Statistical analyses were conducted using Stata 12.0 and SPSS 19.0.

## INTRODUCTION

Recently, non–communicable chronic diseases (NCDs), byproducts of economic growth including cardiovascular disease, cancer, and diabetes, have become the most challenging public health problems and leading causes of mortality worldwide [1]. However, rapid growth of the diabetic population has appeared in underdeveloped countries rather than developed countries, especially in China [2, 3]. Furthermore, nearly all newly diagnosed diabetic patients have T2DM (type 2 diabetes). With rapid economic growth, demographic transition, urbanization, changes in foods and lifestyles, and aging, the prevalence of T2DM in China has been increasing significantly, becoming a serious epidemic that produces a sizable burden on both the individual and the society. The prevalence of diabetes in 1980, 1994, and 2001 was 0.67%, 2.5%, and 5.5%, respectively, doubling relative to the level of the previous year [4–6]. Two recent national surveys conducted in 2008 and 2010 reported that diabetes prevalence continues to increase from 9.7% to 11.6%, 17.3 times higher than the rate observed in 1980 [7, 8]. It is striking that the prevalence of prediabetes has increased sharply from 15.5% to 50.1% in merely two years (from 2008 to 2010) [7, 8]. T2DM has become one of the most important public health crises in China.

Given the rapid and sustained T2DM growth observed over the past 35 years, particularly in China, rather than the slow growth that other countries have (for example, diabetes prevalence of U.S. in 2011–2012 is just 1.27 times higher than in 1988–1994 over the past 25 years [9]), the considerable challenging public health China facing, and the economic problems produced by diabetes in China, data that can represent the national prevalence and the distribution of T2DM over a long period are needed. While some national big surveys of China published were just some fragmented data from a single point in time of estimates. In addition, little is known about the similarities and differences of these trends in China relative to other countries especially in Korea and Japan which have similar genetic backgrounds under different economy and develop speed. With the knowledge of these, it would be easier to evaluate T2DM prevalence under social and economic explosion and to better predict the trend of T2DM prevalence in China.

Therefore, we first conducted a comprehensive systematic review and meta–analysis stratified by age, sex, and geographical area to estimate the history and present situation of T2DM development in China over the past 35 years and predict the trend of T2DM prevalence in the next 10 years. Then we summarized risk factors and complications of T2DM. Besides that, we also estimate the prevalence of T2DM on a national and regional level and address changes over time by comparing the epidemic similarities and differences of T2DM among China, Japan, and Korea.

## RESULTS

The 621 articles cover 11.8 million Chinese people in 31 provinces and municipalities in mainland China, Taiwan, and Hong Kong, as well as Japanese (1.64 million) and Koreans (37.69 million). (Supplementary Tables 1–3 and Supplementary Figure 1).

**Table 1:** T2DM prevalence in China over the last 35 years stratified by year period, age, gender, region, and BMI

Characteristic	T2DM Prevalence % (95% CI)^a^			*I2* (%)			*P* Value for Heterogeneity			*P* Value^b^	*P* Value^c^
	Male	Female	Total	Male	Female	Total	Male	Female	Total		
**Year period**											
1980–1989	1.7 (1.0–2.6)	1.4 (0.9–2.1)	1.3 (1.1–1.6)	99.3	98.6	99	<0.001	<0.001	<0.001	<0.001	<0.001
1990–1999	4.0 (3.3–4.7)	3.7 (3.0–4.4)	4.5 (4.1–4.9)	97.8	97.6	99.4	<0.001	<0.001	<0.001	0.006	
2000–2009	7.0 (6.0–8.1)	6.5 (5.5–7.6)	6.8 (6.4–7.2)	99.9	99.9	99.7	<0.001	<0.001	<0.001	<0.001	
2010–2014	8.6 (7.9–9.3)	7.8(7.0–8.6)	8.7 (8.0–9.5)	99.1	99.4	99.8	<0.001	<0.001	<0.001	<0.001	
**Age**											
15–19	0.4 (0.1–1.0)	0.4 (0.0–1.0)	0.2 (0.0–0.6)	0.0	14.1	60.4	0.931	0.322	0.005	0.960	<0.001
20–29	0.9 (0.6–1.2)	0.7 (0.5–1.0)	0.7 (0.5–1.0)	83.1	80.3	81.7	<0.001	<0.001	<0.001	0.190	
30–39	2.2 (1.4–3.3)	1.7 (1.1–2.5)	1.9 (1.5–2.3)	98.7	98.4	98.8	<0.001	<0.001	<0.001	0.003	
40–49	5.0 (4.4–5.6)	3.6 (3.2–4.0)	4.5 (4.1–4.9)	95.9	93.4	98	<0.001	<0.001	<0.001	<0.001	
50–59	8.3 (7.2–9.4)	8.4 (7.0–9.8)	8.2 (7.6–8.8)	96.9	98.3	98.4	<0.001	<0.001	<0.001	0.04	
60–69	10.5 (9.2–12.0)	12.8 (10.4–13.7)	11.6 (10.7–12.4)	97.1	98.0	98.7	<0.001	<0.001	<0.001	<0.001	
70–79	10.4 (8.2–12.7)	11.8 (9.4–14.5)	12.6 (11.5–13.8)	96.3	97.1	98.5	<0.001	<0.001	<0.001	<0.001	
? ≥80	11.0 (5.1–18.6)	16.1 (1.2–43.2)	9.2 (7.1–11.5)	84.7	96.9	85.2	<0.001	<0.001	<0.001	<0.001	
**Region**											
Urban	8.2 (7.6–8.8)	7.2 (6.5–7.8)	7.4 (7.0–7.8)	99.2	99.3	99.5	<0.001	<0.001	<0.001	<0.001	<0.001
Rural	5.9 (5.4–6.5)	6.0 (5.6–6.5)	5.2 (4.8–5.6)	99.8	99.7	99.9	<0.001	<0.001	<0.001	<0.001	
**BMI**											
<18.5	3.9 (1.7–7.0)	4.5 (1.9–8.1)	2.8 (2.2–3.5)	72.6	80.9	77.5	0.001	<0.001	<0.001	0.800	<0.001
18.5–23.9	6.7 (5.4–8.0)	6.3 (4.2–8.8)	5.6 (5.0–6.1)	91.8	98.3	97.0	<0.001	<0.001	<0.001	<0.001	
24–27.9	11.0 (8.8–13.5)	10.9 (8.1–14.0)	10.4 (9.4–11.5)	95.4	97.3	97.8	<0.001	<0.001	<0.001	<0.001	
≥28.0	12.7 (10.4–15.2)	13.5 (11.3–15.8)	15.4 (13.7–17.2)	93.1	95.5	98.0	<0.001	<0.001	<0.001	<0.001	

^a^ Pooled estimates of T2DM prevalence within the strata of each study characteristic are indicated. ^b, c^ Difference between genders in each stratum and the effect of different stratum on the evaluation of prevalence by using chi–squared (χ^2^) test, respectively.

### Epidemiological characteristics of T2DM in China over the past 35 years

### Current status of diabetes in China, 2014

**Figure 1:**
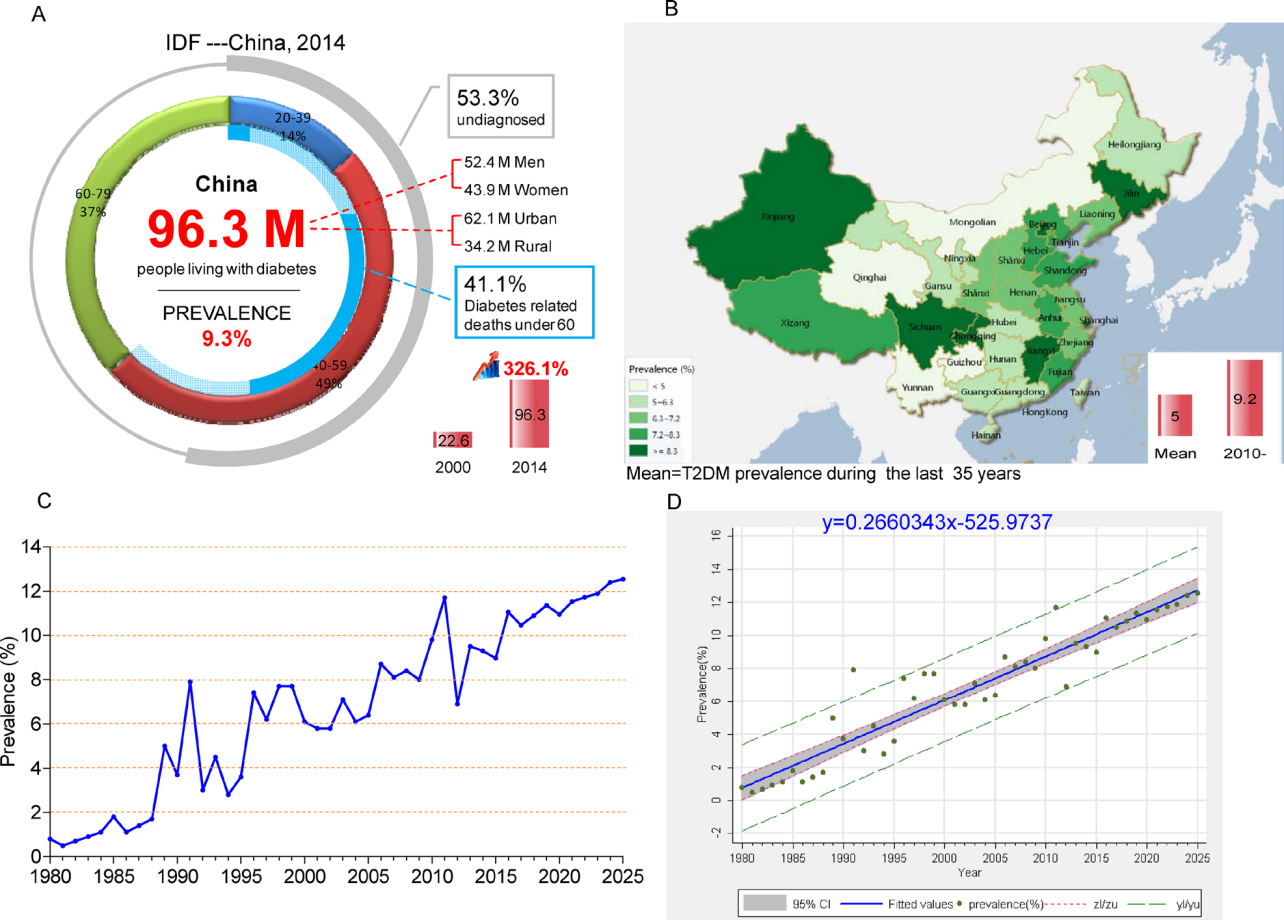
Epidemiological characteristics of T2DM and IGT in China over the past 35 years (**A**) Current status of diabetes in China, 2014; (**B**) Map of T2DM prevalence in mainland China, Hong Kong, and Taiwan over the past 35 years (Map was generated using Dituhui (version 2.0; Dituhui Technology Co., Ltd, Chengdu, China; http://www.dituhui.com)); (**C**) Time series analyses of T2DM prevalence; (**D**) Linear regression showing that T2DM prevalence continues to increase linearly. T2DM prevalence value (%) and 95% CI were marked with green dots and gray area, respectively; the blue solid line stands for fitted values and; gray The red short dash (zl/zu) represents confidence interval; the green long dash (yl/yu) represents prediction interval.

The updated 6th edition of the IDF (International Diabetes Federation) Atlas [10] reported that the diabetic population diabetes in China totaled 96.3 million patients, including 52.4 million men and 43.9 million women (Figure 1A).

Individuals aged 40–59 years old accounted for nearly half of these patients (49%) (Figure 1A). Compared with the year 2000, the number of cases has increased by 326.1% [10]. In addition, most diabetic patients live in urban areas, and the number of diabetic patients in urban areas is nearly twice the level observed in rural areas. If these trends continue, by 2035, some 142.7 million people, or one adult in 11, will have diabetes [10]. Among those under 60 years old, 41.1% of deaths are diabetes related [10]. Over the last 5 years, the doubling of the prevalence of diabetes has increased medical costs by 3.7 times in 2014 relative to 2010 [10]. In the meantime, the IDF reports estimates that more than one–half of all people (53.3%) with diabetes are unaware of their disease and are undiagnosed. Among them, the vast majorities have T2DM.

### Overview of the epidemic of T2DM and prediabetes in China over the past 35 years

From 1980 to 2014, the overall prevalence of T2DM was 5.0%. The highest rates observed in the northern region and southern region are in Beijing (9.1%) and Shanghai (10.8%), respectively. These two cities represent the political and economic centers of these two parts of China. Figure 1B clearly shows that T2DM is mainly limited to coastal cities of mainland China, which is in accordance with the character of economic development (Supplementary Results; and Supplementary Tables 2, 3). The prevalence in most of province and cities is higher than 5.0%, except for people living in Mongolia, Qinghai, Yunnan, and Guizhou, which are low– and middle–income provinces.

### Time series trend of T2DM development in China over the last 35 years and predictions for the next 10 years

To estimate the time series trend of T2DM, we calculated the prevalence of T2DM for each year from 1980 to 2014 by using double arcsine transformed methods and the prevalence values were adjusted by age and gender. Based on these data, T2DM prevalence in the next 10 years for trend was estimated by using a time series regression model. Then, the linear regression model was used to evaluate the T2DM temporal trends from 1980 to 2025. By observing the prevalence trend, we found an obvious linearly increasing trend. An ADF test of the original sequence, without an intercept or the trend of the incidence sequence, suggests the characteristic of one–step difference stationary. To observe the prevalence of one–step differential sequence of the autocorrelation and partial autocorrelation, the one–step moving average and 1:4 step auto regressions is more than 2 times the standard deviation, and ARMA (4, 1) was used to establish the time series regression model. The results show that the regression produces an *R*^2^ of 0.522, AIC of −5.48, and SBC of −5.34, and the model parameters are shown in (Supplementary Figure 4). In conclusion, linear regression showing that T2DM prevalence continues to increase linearly from 1980 to 2025 (*R*^2^ = 0.8938, *P* < 0.0001; y = 0.2660343x–525.9737) (Figure 1C–1D). Therefore, it is projected that the prevalence of T2DM will be at 12.5% in 2025 (Figure 1C–1D, Supplementary Table 4 and Supplementary Figure 4).

### The distributions of T2DM and IGT prevalence stratified by year, age, gender, and region over the past 35 years

**Figure 2:**
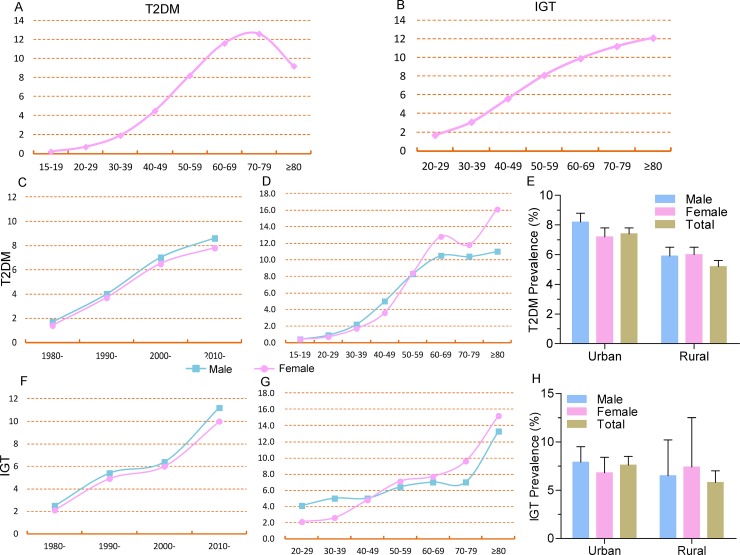
Distribution of T2DM prevalence by age, year, gender, and region (**A**) T2DM prevalence increased with aging but decreased after age 80; (**B**) IGT prevalence increased with aging; (**C**) T2DM prevalence increased in both men and women by year, and men appeared more likely to develop T2DM; (**D**) Men appeared more likely to develop T2DM with aging up to age 50, and T2DM prevalence in women was higher than in men after 50; (**E**) T2DM prevalence was higher in men than in women in urban areas but was same in rural areas; (**F**) IGT prevalence increased in both men and women by year, and men appeared more likely to develop IGT; (**G**) Men appeared more likely to develop T2DM with aging before 50, and T2DM prevalence in women was higher than men after age 50; (**H**) T2DM prevalence was higher in men than in women in urban areas but was lower than women in rural areas.

Increasing prevalence of both T2DM and IGT was seen by year and with aging (*P* < 0.001) (Figure 2A–2B, Table 1, Supplementary Table 5 and Supplementary Figures 2, 3). Prevalence of T2DM in 2010–2014 was almost 7 times that in 1980–1989. Unlike the increasing trend in IGT beyond age 80 and continuing growth among those between the ages of 40 and 79, T2DM prevalence started to decrease from 12.6% (95% CI, 11.5%–13.8%) among people 70–79 years old to 9.2% (95% CI, 7.1%–11.5%) among people over 80 years old (Figure 2A).

The prevalence of T2DM and IGT were found higher in men than in women from 1980–1989 to 2000–2009 (all *P* < 0.05 for χ^2^ in each year period stratum). Since 2010, the gender difference has started to widen, with 8.6% (95% CI, 7.9%–9.3%) in men and 7.8% (95% CI, 7.0%–8.6%) in women for T2DM (*P* < 0.001 for χ^2^); while the IGT prevalence was 11.2% (95% CI, 6.6%–16.9%) in men and 10.0% (95% CI, 5.4%–15.7%) in women for IGT, respectively, with no significant difference (*P* = 0.91) (Table 1, Figure 2C and 2F, and Supplementary Figure 4). As for age distribution, no significant difference was found between men and women before age 29 and the prevalence of T2DM showed higher in men than in women from age 30 to age 50 (all *P* < 0.05 for χ^2^ in each age stratum). Interestingly, the reverse was observed among those over 50 years old. Women had a higher T2DM prevalence than men, and the gap between them continued to increase with aging (all *P* < 0.05 for χ^2^). No significant difference of IGT prevalence was found between men and women in each age stratum (Figure 2D and 2G).

Concerning differences by region, the prevalence of T2DM and IGT were 30% and 24% higher, respectively, in urban areas than in rural areas (*P* < 0.001). In general, the rural prevalence of T2DM in women was higher than in men (*P* < 0.05 for χ^2^). T2DM prevalence in urban areas was much higher in men while IGT prevalence showed no significant differences between men and women (Figure 2E and 2H). No significant difference was found with the prevalence value of T2DM as for the different diagnosis criteria (*P* = 0.061) and diagnosis criteria in different years (all *P* > 0.05).

### The risk factors for and complications of T2DM over the past 35 years

**Figure 3:**
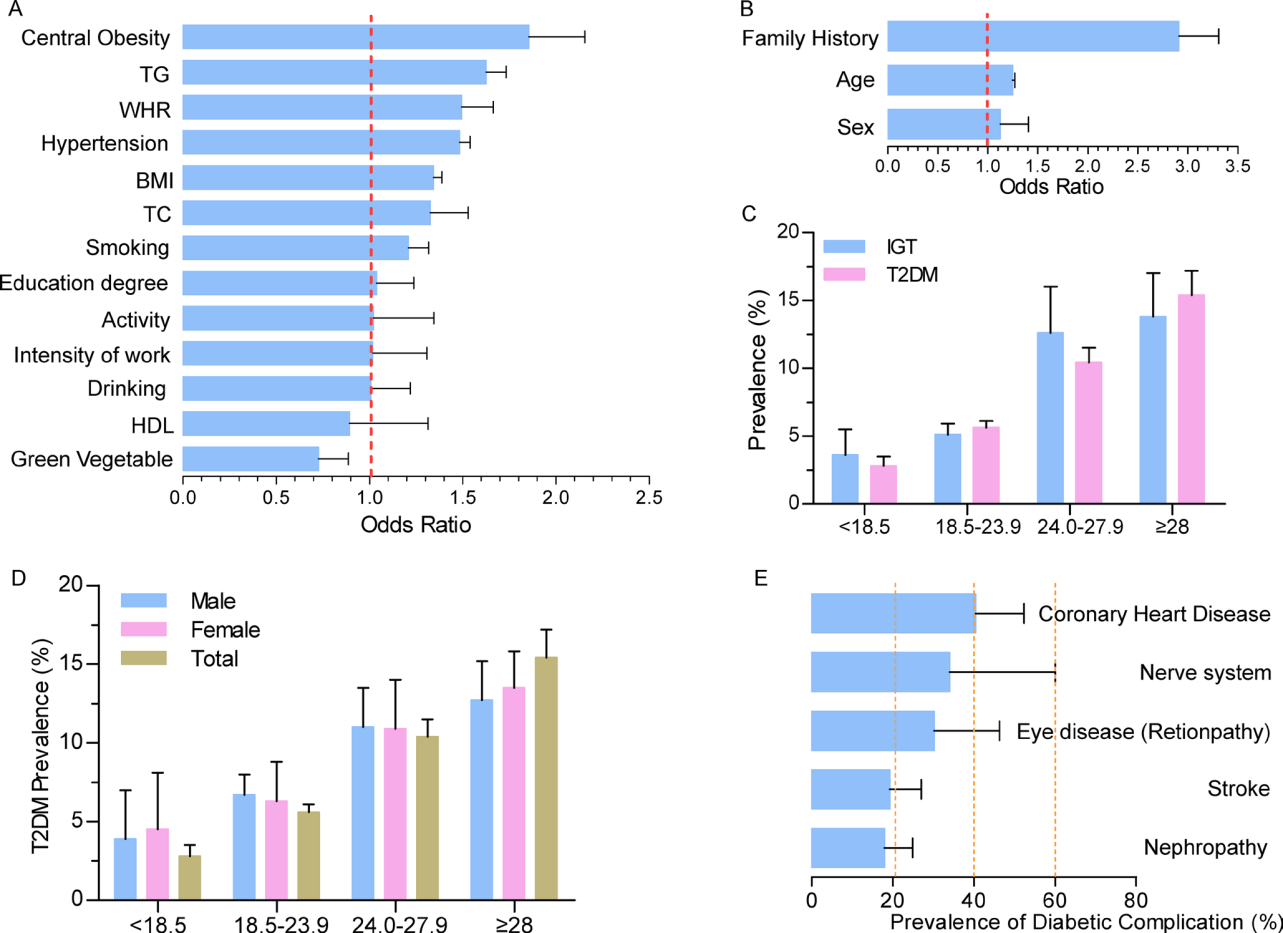
Risk factors for and complications of T2DM (**A**) Potential controllable risk factors contributing to T2DM; (**B**) Potential uncontrollable risk factors contributing to T2DM; (**C**) BMI is positively correlated with T2DM and IGT prevalence; (**D**) BMI is positively correlated with T2DM and IGT prevalence in both men and women. Women with very low and very high BMIs were more likely to develop T2DM; (**E**) Diabetic complications.

In addition to uncontrollable factors, central obesity was the primary potentially modifiable risk factor (OR = 1.85, 95% CI, 1.60–2.16) (Figure 3A). The major uncontrollable driving factor of T2DM is a family history, with a high OR = 2.91 (95% CI, 2.56–3.3) (Figure 3B). Gender and age contributed 12.3% and 24.6% increases in T2DM risk, respectively.

Then, we analyzed how obesity contributed to T2DM and IGT in detail. Compared with normal values (BMI = 18.5–23.9), the prevalence of T2DM nearly doubled among the overweight (BMI = 24.0–27.9) and tripled among the obese (BMI ≥28) (Figure 3C and 3D). Notably, however, a higher prevalence of 5.6% was also seen among people with normal BMIs (95% CI, 5.0%–6.1%). IGT prevalence increased with the BMI increment in the same way as T2DM, except for moderate growth observed from the shift from overweight to obese. Women with very high BMIs (≥28, 12.6%) appeared more likely to develop T2DM than men (13.5% in men and 12.7% in women, respectively, *P* < 0.001) (Figure 3D).

Due to a lack of epidemiological data on T2DM–related complications in China, we next summarized studies that provided data on complications over the past 35 years. Topping the list of complications was coronary heart disease, with a prevalence of 40.4% (95% CI, 28.5%–52.3%). The prevalence of diabetic complications, such as nerve system disease and eye disease reached 30% or more (Figure 3E).

### Chinese characteristics of T2DM

### Comparison with Japan and Korea

Consistent with the patterns characterized for China, T2DM prevalence in Japan increased with aging, and men were more likely to develop T2DM, controlling for advancing age and living area (Supplementary Figure 6). The prevalence of T2DM in Korea showed a similar trend as in China (Supplementary Figure 6).

Except for uncontrollable factors such as age, our results indicate that T2DM in Japan has remained stable overall since 1990, increasing slightly over the 2010–2014 period (Supplementary Figure 6). However, Japan has unique characteristics. First, sustained growth of T2DM prevalence ended by the 1970–1979 period. This was followed by a stable period in 1980–1989 and a decrease in 1990–1999. The prevalence has remained stable for approximately 20 years. Over the 2010–2014 periods, the prevalence of T2DM has started to increase slightly. Second, diabetic patients in Japan were younger than in China, and the prevalence was 7.9% (95% CI, 5.1%–10.6%) among those 30–59 years old. A higher prevalence was observed among the Japanese than among Chinese people of the same age. Third, the prevalence of diabetes in urban and rural areas was 7.7% (95% CI, 6.6%–8.8%) and 12.2% (95% CI, 8.7%–15.6%), respectively, which is the opposite pattern observed in China.

As for relatively developed Korea, the prevalence trend of T2DM is similar to or even higher than that of China (Supplementary Figure 6). Increasing prevalence was observed with increasing age and by year. The prevalence of T2DM in urban areas was higher than that in rural areas (15.4%, 95% CI, 12.3%–18.5% and 11.7, 95% CI, 7.2%–16.2%, respectively). One significant difference was that the T2DM prevalence in Korea was much higher than the level for the corresponding year or age in China. Another significant difference was that there seemed to be no obvious difference between men and women.

In addition, we analyzed the IDF data on diabetes in the U.S. and found that the prevalence of diabetes has remained stable at approximately 11% since 2011. Interestingly, the same situation was also found in Japan. The IDF report showed that with the exception of an unexpected increase in 2011, the prevalence of diabetes in Japan has been approximately 7.5% since 2000. Then we compared the urbanization and GDP (gross domestic product) level between them which correlated closely with T2DM prevalence and found that the proportion of urban population was over 70% from 1980 in Japan and 1960 in U.S., respectively. However, the proportion in China is only 53% in 2013. In addition, economic growth rate was stable in both Japan and U.S. from 2000 while China and Korea were still in the period of rapid economic growth (Supplementary Tables 6, 7). Therefore, urbanization and the level of economic growth have great influence on the prevalence of diabetes.

## DISCUSSION

Our 35–year analyses of diabetes in China indicates that the prevalence of T2DM has increased sharply from 1.3% in 1980–1989 to 8.7% in 2010–2014, and the overall prevalence of IGT doubled approximately once per decade. T2DM prevalence will continue to increase in a linear way and it will be at 12.5% in 2025.

In our study, we also compared across countries, such as Japan and Korea, which have similar racial origins. Then, what have we learned from the study of T2DM prevalence through a comparative lens? First, there is a positive correlation between increased T2DM prevalence and the economy development (GDP/GNI (gross domestic product/gross national income)) and urbanization which are associated with unhealthy lifestyle. Second, prevalence in rural areas is now rising and should not be ignored. Third, T2DM trends in the U.S. and Japan happily reveal that the T2DM growth rate will remain stable. Recent studies from the United States demonstrate that obesity prevalence appears to have stabilized [11], which may contributing to the stabilizing prevalence of metabolic syndrome [12]. With economic development, social progress, medical improvement, and investment in the healthcare system, as well as effective countermeasures to control high risk factors, the future of China can be healthy. Emerging countries would benefit from China's experience to prevent the rapid growth of T2DM.

### Our study had the following noteworthy strengths

First, this is the first longitudinal time series analysis using detailed data on T2DM and IGT in China over the past 35 years. We performed the longitudinal and continuous analyses through the past and present stratified by year period, age, region, and BMI. Then we forecast the T2DM development trends will continue increasing linearly in the next 10 years.

Second, at present, increasing life expectancy, which is leading to China's aging population, contributes the increase in diabetes risk [13]. Therefore, characteristic of T2DM prevalence with the changes in the population structure of age need to be better understood. In addition, the whole course scientific management of life requires close attention to a “life–course strategy” that requires more detailed data about diabetes development with aging. Given the above requirements, our study analyzed data from individuals 15 to over 80 years old stratified by sex and demonstrated that men are more likely to develop T2DM in 30–50 years old, but after the age range of 50–59, the number of diabetic women increased significantly. Being male, contrary to common beliefs, was no longer a sustained risk factor for T2DM with aging. More prone to obesity induced by change of sex hormone levels in postmenopausal women and longer life of women might be the contributing factors which can explain this phenomenon. To achieve “Successful Aging and Active Aging”, we should pay more attention the blood glucose monitoring of middle–aged men and elderly women.

Third, we conducted a lateral analysis at the same time to evaluate the role of China in global diabetes trends. Through the analysis of Japan and Korea, as well as of the status of and trends in the U.S., we could speculate that there might be a plateau in T2DM prevalence with highly developed economies, cultures, societies, and especially, well–established healthcare systems. The present status of diabetes in Korea represents the near future of China, while China will reach the plateau period, as Japan and the U.S. have, far in the future. Although lifestyle interventions have proved effective in Daqing [14], widespread implementation requires the involvement of communities and governments [15]. China should be more active to reach the plateau period smoothly; otherwise, China would walk out the same way as Pima Indian (T2DM prevalence was as high as 50%) [16] has walked in due to the same origin of human race. So, adding the horizontal comparison on the basis of the inter–country vertical comparison of the time dimension (35 years of prevalence trend) in the ultimate prevalence analyzing to get more comprehensive evaluation results.

Fourth, because T2DM is a so–called “modern disease” that closely follows socioeconomic development, it should be prevented and controlled by personalized measures for different areas due to the uneven development of regional economies in China. The GDP map indicates that socioeconomic is higher in eastern cities of China than in other cities. Correspondingly, T2DM prevalence is also higher in coastal cities of China. Notably, the strategy of “One Belt And One Road” (OBAOR) proposed in 2013 will speed up the economic development of western China and will in turn increase east–west exchanges. Therefore, more attention should be paid to the trend of the diabetes epidemic in western China.

Finally and most importantly, our study identified some potentially effective prevention and control strategies to curb the fast growth of T2DM. Caloric restriction, reasonable and healthy diets, and lifestyles changes including increased leisure time, physical activity and reduced time in front of the screen are recommended to reduce the occurrence of central obesity and subsequent T2DM [17]. By learning some Japanese and American lessons, building and improving a sophisticated healthcare system to serve and benefit everyone in China seems particularly important. China should thus promote the reform of its healthcare system quickly and invest in expanding health insurance coverage. For example, a primary health care model should be established to replace the current hospital–centric system [18]. Furthermore, low rates of awareness, treatment, and control (fewer than 50%) and a high rate of under diagnosis (more than 50% in 2014) should not be ignored. By contrast, the rate of undiagnosed diabetes is 20% in the U.S., which reduces treatment delays and subsequent invasive care, higher costs, and worse outcomes [18]. Nationwide propaganda and education about T2DM and routine physical examination for high risk populations should be encouraged and applied first in the underdeveloped areas of western China.

A limitation of our study includes potential non–exhaustive evaluation of T2DM trends in Japan and Korea over the past 35 years. We searched only for full articles written in English for Japan and Korea, which would lead to a lack of data for stratified analyses. For example, data for Korea were missing before 1990, and few studies were available to evaluate the differences between men and women with advancing age.

## METHODS

### Search strategy

We performed a systematic review to identify all relevant studies that reported the prevalence of diabetes. According to PRISMA guidelines, the PubMed, CNKI, WANFANG, International Diabetes Federation (IDF) [19], National Bureau of Statistics of the People's Republic of China (NBSC) [20], the International Monetary Fund (IMF) [21], and Global Finance (GF) [22] databases were searched using the keywords “diabetes” and “prevalence” and “China/Japan/Korea”. We searched PubMed for articles written in English, as well as WANFANG and CNKI for articles written in Chinese, published between January 1, 1980, and December 31, 2014, with the search terms “diabetes”, “Chinese”, and “prevalence”. We also searched PubMed for articles written in English published between January 1, 1980, and December 31, 2014, with the search terms “diabetes”, “Japanese” (“Koreans”), and “prevalence”. Additional studies were retrieved by manually searching the references of selected articles. To avoid repeated studies, we also conducted a manual search (including checking author lists, study design, and publication date, etc.) among the studies from the same area to ensure there were no overlapping participants in the included studies. For overlapping studies, the most recent publication or the largest sample size publication providing more information was selected.

### Inclusion criteria

To include all the relevant studies and reduce deviation, the identified studies should meet the selection criteria: 1) studies investigating the prevalence of T2DM or impaired glucose tolerance (IGT); 2) participants and cases should be ≥15 years of age; 3) studies with clearly reported diagnostic methods and criteria for T2DM; 4) studies providing detailed information about number of participants and T2DM/IGT cases.

### Data extraction

Two reviewers (HY and XL) independently extracted data. We extracted the following data from all identified studies: study location, survey date, diagnostic criteria, age range, number of participants and cases for both T2DM and IGT, number of diabetic complications, and odds ratio (OR) with 95% CIs for risk factors. For data of the same period in the same area, we carefully investigated the author list and their affiliation to avoid the repeated inclusion of the same data. Meta–analyses were conducted to estimate the prevalence of diabetes with 95% CIs stratified by age, gender, period (1980–1989, 1990–1999, 2000–2009, 2010–2014), and region. Data of IDF, NBSC, IMF, and GF were extracted from reports open published by these institutions.

### Statistical analysis

First, we performed a systematic review and meta–analysis of published studies of T2DM and IGT in mainland China, Hong Kong, Taiwan, Japan, and Korea from 1980 to 2014. The prevalence of T2DM with 95% confidence intervals (CIs) was estimated based on polled data from all identified studies. To carry out the meta–analysis for T2DM prevalence, the prevalence proportion of each eligible study was double arcsine transformed to stabilize variances and the inverse of the variance of transformed proportion was used as study weight [23]. Then the polled transformed proportion and its CIs are back transformed to a prevalence value. To predict the T2DM trends in China in the near future, the prevalence of T2DM for each year from 1980 to 2014 was also calculated using double arcsine transformation method as mentioned above. Based on the 35 years of data, we estimated the T2DM prevalence in the next 10 years for trend by using a time series regression model. Then, the linear regression model was used to evaluate the T2DM temporal trends from 1980 to 2025.

The T2DM/IGT prevalence estimates were stratified by year period, age, region, BMI, and diagnostic criteria as follows. For temporal prevalence trends estimates, 35 years were grouped into 4 time periods: 1980–1989, 1990–1999, 2000–2009, and 2010–2014. We also analyzed the T2DM/IGT prevalence stratified by age (15–19, 20–29, 30–39, 40–49, 50–59, 60–69, 70–79, and ≥80), gender (male/female), region (urban/rural), and BMI (<18.5, 18.5–23.9, 24.0–27.9, and ≥28 kg/m^2^). Owing to the different diagnosis criteria for T2DM in the eligible studies, we performed the meta–analysis stratified by diagnosis criteria. 5 subgroups for diagnosis criteria was defined as follows: Chinese Diabetes Society (CDS), American Diabetes Association (ADA), World Health Organization (WHO), International Diabetes Federation (IDF), fasting plasma glucose (FPG ≥ 7.0 mmol/L), and FPG (FPG ≥ 7.0 mmol/L) or/and oral glucose tolerance test (OGTT ≥ 11.1 mmol/L.). FPG and FPG with OGTT were listed separately owing to some studies only described the diagnosis methods for T2DM. Besides that, we collected all studies from China which provided odds ratio (OR) for potential risk factors for T2DM during the past 35 years and studies which provides prevalence of diabetic complication to seek for the key etiological factors for T2DM and its popular complication which we should pay more attention.

The heterogeneity between studies was estimated using a Cochran *Q*-test and the I2 statistic. Random–effects models introduced by DerSimonian and Laird were applied for the incorporation of between–study heterogeneity and to obtain an overall prevalence of T2DM, overall OR for the risk factors of T2DM, and T2DM complication. To find out the possible heterogeneity usually existed in the meta–analysis for the prevalence, meta–regression stratified by different characteristics was performed to find out the potential source of between–study heterogeneity and the proportion it could explained for the heterogeneity. In addition, considering the same diagnosis criteria have been changed several times in the past 35 years, we also performed the meta–regression stratified by different diagnosis criteria in different years.

The sensitivity analyses were also conducted to assess each study and evaluate a polled estimate for the remaining studies. Publication bias was evaluated by Egger's linear regression test. Because of the very different prevalence of T2DM in different category of year, age, region, BMI, etc., the sensitivity analyses and publication bias were calculated stratified by different characteristics.

In addition, we analyzed the present global situation we are facing concerning T2DM and IGT. Data including population, diabetes cases, diabetic prevalence, diabetes–related deaths, and cost per person with diabetes from the global, China mainland, Taiwan, Hong Kong, Japan, and Korea were extracted from 6 open published editions of “diabetes Atlas” since 2000 from IDF website. In addition, we also collected data about economics and demography of corresponding period from NBSC (i.e. 35 years of gross domestic product (GDP) and gross national income (GNI) data in China), IMF (i.e. population, urbanization trends and GDP data in different country), and GF (i.e. GDP data in different country). Analyzing those available data, on the one hand, we tried to elucidate the relationship between T2DM increase and economic development, as well as the burden of T2DM in China; on the other, predict the T2DM trends of China and get some clues by comparing with Japan, Korea, and the U.S in detail. We compared China with Japan and Korea stratified by diabetic prevalence, prevalence trend, percentage increase, diabetes–related deaths, and cost per person with diabetes.

The difference of T2DM prevalence between subgroup was estimated by chi–squared (χ^2^) test. Two–sided tests were used, and *P* < 0.05 was regarded as statistically significant. Statistical analyses were conducted using Stata (version 12.0; StataCorp LP, TX, USA) and SPSS (version 19.0, SPSS Inc., Chicago, IL USA).

## CONCLUSIONS

China has undergone rapid development of T2DM and IGT and is playing a key role in global diabetes trend. From 1980–1989 to 2010–2014, the prevalence of T2DM increased from 1.3% to 8.7%. IGT prevalence doubled every 10 years, reaching a high level of 13.6% over the 2010–2014 periods. We estimated that by 2025, T2DM prevalence will have grown to 12.5%. According to our results, there will be more elderly women patients (from 50–59 years old) and more diabetic women with very high BMIs (BMI ≥ 28 kg/m^2^). Central obesity is the largest preventable cause of T2DM, followed closely by TG (triglyceride), WHR (Waist–to–Hip Ratio) and hypertension. More importantly, to arrive at the plateau period, as Japan and the U.S. have, more smoothly, healthcare system reform, public health policies, awareness campaigns, and prevention programs (i.e., caloric restriction and healthy lifestyle) uniquely designed for modern–day China will be essential.

## SUPPLEMENTARY MATERIALS FIGURES AND TABLES





## Abbreviations

ADA: American Diabetes Association
BMI: Body Mass Index
CIs: Confidence Intervals
CDS: Chinese Diabetes Society
FPG: Fasting Plasma Glucose
GNI: Gross National Income
GDP: Gross Domestic Product
IGT: Impaired Glucose Tolerance
IDF: International Diabetes Federation
NCDs: Non–Communicable Chronic Diseases
OGTT: Oral Glucose Tolerance Test
T2DM: Type 2 Diabetes
TG: Triglyceride
WHR: Waist–to–Hip Ratio
WHO: World Health Organization
