# Supplementary material for: Type 2 diabetes epidemic in East Asia: a 35–year systematic trend analysis
Source: Oncotarget. 2017 Dec 6;9(6):6718–27. doi: 10.18632/oncotarget.22961 (PMC5805508; doi:10.18632/oncotarget.22961)
Supplement: Supplementary file 1 [file oncotarget-09-6718-s001.pdf]

Type 2 diabetes epidemic in East Asia: a 35–year systematic trend analysis

SUPPLEMENTARY MATERIALS

We included data from the 6th edition of the "*IDF Diabetes Atlas*" [1], the National Bureau of Statistics of the People's Republic of China [2], the International Monetary Fund [3], and the Global Finance [4] 6 databases. The prevalence of T2DM and IGT, with 95% CIs, was calculated for each study (Table 1, Supplementary Table 5, and Supplementary Figure 1).

Epidemiological characteristics of T2DM in China over the past 35 years

Overview of the epidemic of T2DM and prediabetes in China over the past 35 years

With continuous growth of gross national income (GNI) (from 4551.6 billion RMB in 1980 to 634367.3 billion RMB in 2014) from 1980–1989 and 2010–2014, overall prevalence of T2DM in China increased markedly (584.6% increase) from 1.30% (95% CI, 1.10%–1.40%) in 1980–1989 to 8.9% (95% CI, 8.4%–9.5%) in 2010–2014 [2] (Supplementary Figure 2). Meanwhile, the overall prevalence of IGT doubled approximately once per decade. During this period, T2DM and IGT prevalence rose with income and age (Supplementary Figure 2). The prevalence of diabetes and prediabetes in urban areas are 8.1% (95% CI, 7.8%–8.4%) and 8.2% (95% CI, 7.5%–8.9%), higher than the 5.6% (95% CI, 5.0%–6.2%) and 6.2% (95% CI, 5.3%–7.1%), respectively, observed in rural areas (Supplementary Figure 2). Among the Han ethnic group, prevalence is twice as high as other minority groups and 11.7 times higher than among the Uygur ethnic group, which may due to differences in genetic backgrounds, lifestyle habits, and diets (Supplementary Figure 2).There was no significant difference in prevalence between the north and the south of mainland China (7.3% and 6.9%, respectively) (Supplementary Figure 3).

With increasing gross domestic product (GDP) and gross national income (GNI), the T2DM prevalence has greatly affected the country and society as well as individuals' lives in mainland China (Supplementary Figure 3). Overall health expenditures increased by 183.7% from 2000–2009 to 2010–2014 regardless of where people lived [2]. Both the government and individuals have had to pay more than ever for healthcare. T2DM-related costs account for the major of these expenditures.

Awareness rate, treatment rate, and control rate of T2DM over the past 35 years

Unfortunately, awareness rate, treatment rate, and control rate of diabetes remained very low at 44.4% (95% CI, 36.4%–52.4%), 41.9% (95% CI, 36.1%–47.6%), and 24.7% (95% CI, 20.3%–29.1%), respectively. In general, from 1990–1999 to 2010–2014, the pooled estimate for awareness of T2DM increased from 33.8% (95% CI, 33.1%–36.5%) to 49.5% (95% CI, 34.3%–64.6%); the treatment rate increased from 26.5% (95% CI, 23.7%–29.4%) to 45.1% (95% CI, 33.6%–56.5%); and the control rate increased from 7.6% (95% CI, 3.5%–11.7%) to 16.2% (95% CI, 2.9%–29.4%). Although the awareness rate increased, the treatment rate stagnated beginning in 2010, and the control rate even declined over the 2010–2014 period. Moreover, the treatment and control rates decreased slightly from 2000–2009 to 2010–2014. Much higher rates of awareness, treatment, and control were found for women than for men. Although the rates of awareness and treatment were higher in urban areas, people living in rural areas had better-controlled T2DM than those in urban areas (25.4%; 95% CI, 12.5%–38.3% and 20.7%; 95% CI, 11.7%–29.7%, respectively) (Supplementary Figure 5).

Chinese characteristics of T2DM

The role of China in the global prevalence of diabetes

Supplementary Table 6 presents global estimates of diabetes and IGT from 2000 to 2035. Health spending on T2DM accounted for the majority of expenditures and reached 421.3USD/person in 2014, nearly 4 times that in 2010.

The number of diabetes cases increased with population (Supplementary Figure 6). From 2000 to 2013, number of people with diabetes has risen from 150.9 million to 381.8 million, with prevalence increasing from 4.6% to 8.3%. Meanwhile, the number of people with IGT remained stable from 2000 (314 million) to 2013 (316

million), and prevalence of IGT decreased by 15.9% (8.2% and 6.9%, respectively). The estimated prevalence of diabetes and IGT will increase to 10.1% and 8.0%, respectively, by 2035. The number of people with diabetes and IGT will reach 591.9 million and 471.0 million, respectively, by 2035.

The diabetic population in China always accounts for one-quarter of the world's total population despite rapid changes in the world. The prevalence of T2DM has risen sharply over the past 30 years (prevalence in 2010 was 17.3 times that of 1980). This "T2DM explosion" happened differently in China than in other countries.

Rapid growth of diabetes has imposed considerable costs in terms of lives and economic burdens on both society and individuals. On the one hand, diabetes has produced enormous public health consequences in China. Diabetes-related deaths have also increased over the last 5 years, accounting for 41.1% of deaths among those less than 60 years old. On the other hand, diabetes has become an economic burden on everyone. Excluding Hong Kong and Taiwan, the mean expenditures on diabetes per person continues to rise over the period between 2010 and 2014. Health expenditures in China increased from 699.56 RMB/person in 2000–2010 to 1925.26 RMB/person in 2010–2014 [2].

Urbanization and increasing GDP are positively correlated with the prevalence of both T2DM and IGT (Supplementary Figure 6 and Table 7). Japan experienced a rapid increase in urbanization over the 1960–1980 periods, which led to rapid growth in T2DM prevalence. China and Korea are undergoing similar urbanization, but the prevalence is growing more rapidly in Korea than in China. The urban population in 2013 accounted for 92%, 82%, and 53% in Japan, Korea, and China, respectively.

As shown in Supplementary Figure 6, the latest data showed that global adult diabetes cases will increase by 53.1% in 2035 compared to 2014, reaching 592 million patients. Although the adult population in China is nearly 10 times as large as Japan's and 27 times as large as Korea's, diabetes-related deaths are nearly 20 times higher in adults in China than in Japan and 40 times higher than in Korea (Supplementary Figure 6 and Table 7).

Fortunately, the population of each country remained stable from 2010 to 2014. In addition, the prevalence of T2DM in highly urbanized U.S. with high GDP also appeared to decrease and stabilize since 2011 and then maintained at approximately 11%. Supplementary Table 6 shows the updated data from IDF reports, 2014. Compared with Japan, Korea, Hong Kong, and Taiwan, China has the highest number of diabetes patients and the highest diabetic prevalence; however, mean expenditures were only 421.3 USD/person in 2014. Although the comparative prevalence of diabetes in Japan is the lowest in this group (5.1%), its medical costs of diabetes per person are the highest (4908 USD/person).

Heterogeneity and publication bias

As we have seen in other meta-analysis for prevalence proportion, the heterogeneity was also found in this study. Almost all of *I* square (I^2) were more than 90% with *P* values of heterogeneity were less than 0.001 which showed the substantial heterogeneity existed in the eligible studies. As showed in Table 1 and Supplementary Table 5, there were different characteristics in each study such as year, age, region, BMI, and diagnostic criteria both in T2DM and IGT. To find out the potential source of heterogeneity, meta-regression analyses were performed. Supplementary Table 8 showed gender was not the main source of heterogeneity while year period, age, region, and BMI were the main factors influencing the heterogeneity (all $P < 0.001$ for meta-regression) and could explain 13.8%, 46.7%, 3.21%, and 40.51% of heterogeneity, respectively. Diagnostic criteria and diagnostic criteria in different years would not bring about heterogeneity (all $P > 0.05$ for meta-regression).

Sensitivity analyses to assess the effect of omitting individual studies on the estimate of prevalence were also performed in this study in each characteristic stratum. The results showed no individual study dramatically influenced the overall prevalence.

Funnel plot asymmetry (data not shown), the Begger's test, and the Egger's test were conducted stratified by subgroups for different characteristics. Publication bias was seen in most of our meta-analysis (Supplementary Table 9). Considering this study is a meta-analysis for prevalence proportion and the publication bias would not be involved in the definition of the causal relationship, we just analyzed the possibility of publication bias.

REFERENCES

1. IDF Diabetes Atlas. http://www.idf.org/diabetesatlas (accessed July, 2015).
2. Ma RC, Lin X, Jia W. Causes of type 2 diabetes in China. Lancet Diabetes Endocrinol. 2014; 2:980–91.
3. Li G, Zhang P, Wang J, Gregg EW, Yang W, Gong Q, Li H, Jiang Y, An Y, Shuai Y, Zhang B, Zhang J, Thompson TJ, et al. The long-term effect of lifestyle interventions to prevent diabetes in the China Da Qing Diabetes Prevention Study: a 20-year follow-up study. Lancet. 2008; 371:1783–9.
4. Herman WH, Zimmet P. Type 2 diabetes: an epidemic requiring global attention and urgent action. Diabetes Care. 2012; 35:943–4.
5. Hu X. Investigation of hypertension and diabetes in Tongcheng, Tianchang. Anhui J Prev Med. 2013; 19:389–90.
6. Ding Y, Yan CR, Wang YM, Ning G, Qiao J, Yu B. Prevalence of diabetes and related risk factors in elderly people in a community of Hefei. Chin J Clin Healthe. 2013; 16:306–8.
7. Ying YC, Yu BF, Mei Z, He QJ, Zhou XH, He F, He J, Wang DN, Wang YM, Ning G. Investigation and analysis on the prevalence of diabetes and impaired glucose regulation in the

residents aged over 40 years in Hefei City. Anhui Medical Journal. 2012; 33:1368–70.

8. Liao YQ, Ni X. Fasting blood glucose test results of the analysis in 3799 cases of the residents of southern Anhui Clinical Laboratory Journal(Electronic Edition). 2013; 2.
9. Xu W, Yuan HM, Cao ZQ, Wang AL, Wu QS, Jin S. Analysis of prevalence and risk factors of diabetes mellitus in some district of Ma'anshan. Anhui J Prev Med. 2012; 18:239–41.
10. Wang W, Xu J, Jiang Q. Analyzing the result of diabetics screening in village clinics of one county in Anhui Province. The Chinese Health Service Management. 2011; 7:548–50.
11. Li YC, Hu CL, Jiang JH, Zhang B, Xiao YK, Cha T. Analysis on prevalence of type 2 diabetes and its risk factors among senior intellectuals from a university. Modern Preventive Medicine. 2006; 33.
12. Hou D. Investigation of Diabetes Mellitus of Senior Intellectuals in Hefei Industry University. Anhui J Prev Med. 2003; 9:230–1.
13. Huang YQ, Zhang XM, Song HD, Shi JH, Mu LP, Su Q, Zhao M. Two epidemiologic surveys of NIDDM in Bengbu. Journal of Bengbu Medical College. 1996; 21:422–4.
14. Pan CY, Tian H, Xu XJ, Lu J. Prevalence and incidence of diabetes in the elderly in Beijing army. Chin J Geriatr. 2003; 22:364–7.
15. An JJ, Xiao GY, Li J, Wang JJ, Feng Y. Current situation of chronic disease of 2010 and comparison with that of 2002, in Fengtai District, Beijing. Modern Preventive Medicine. 2013; 40:4393–400.
16. Li SB, Xu ZX, Pang W. Dynamic monitoring of diabetic patients in Changping District of Beijing City. Chinese Journal of General Practice. 2013; 11:758–9.
17. Wang QH, Li XP, Li XF, Wang L. Prevalence of hypertension and diabetes mellitus of staff in Beijing Normal University. Chin J School Doctor. 2013; 27:193–6.
18. Li L. Analysis of 500 cases with chronic disease and investigation of health mamagement model. Chinese Medicine Guide. 2012; 10:143–5.
19. Qiao L, Yu Z, Wang XH, Yang XJ, Zhang LY, Hu W, Wu YW, Kong LZ, Du SM, Ma G. The Prevalence of Non-communicable Diseases among the Floating Population in Urban Beijng, China. Chin J Prev Contr Chron Dis. 2010; 18:111–4.
20. Liu F, Lan TX, Liu A. The Prevalence of Chronic Non-communicable Diseases among the Inmates in Beijing. Chin J Prev Contr Chron Dis. 2012; 20:277–83.
21. Zhan YQ, Yu JM, Hu DY, Sun YH, Fu YY, Zhang LJ, Li SC, Zhang F. A cross-sectional study: the prevalence of impaired fasting glucose and diabetes mellitus among residents, Beijing. Chin Prev Med. 2010; 11:1218–21.
22. Zhou X, Ji L, Luo Y, Han X, Zhang X, Sun X, Ren Q, Qiao Q. Risk factors associated with the presence of diabetes in Chinese communities in Beijing. Diabetes Res Clin Pract. 2009; 86:233–8.
23. Ding SQ, Yang XJ, Xing LL, Pan J. Study on non-communicable disease prevalence and risk factor in Dongcheng District,Beijing. Chinese Journal of Health Education. 2012; 28:188–90.
24. Su Y, Wang TQ, Du SM, Gao J, Tian Y. Investigat ion of residents'health of Beijing certain community. Clinical Focus. 2010; 25:754–7.
25. Wang H. Prevalence of diabetes and its related risk factors in Shunyi district,Beijing. Capital Journal of Public Health. 2010; 4:58–60.
26. Zhang GX, Chen H. Investigation and study on the knowledge of diabetes mellitus in Shunyi District. Medical Journal of Chinese People's Health. 2006; 18:780–1.
27. Ruan DJ, Yang Z. The prevalence of diabetes in 20-70 years old chinese population living in Huairou District of Beijing. Beijing Medicine. 2010; 32:297–300.
28. Han X. The epidemiologic feature of diabetes mellitus morbidity in a town. Chinese Medicine Guide. 2012; 10:73–4.
29. Heng W, Ren N. Screen and management of 342 high-risk individuals for diabetes at Sanyili community of Xuanwu District Journal of Clinical and Experimental Medicine. 2010; 9:472–3.
30. Wang W, Tian YF, Li W. Epidemiological study of diabetes in Xuanwu district of Beijing city, China. Hainan Medical Journal. 2012; 23:138–9.
31. Jia J. Epidemiological survey for diabetes in Haidian District, Beijing. Modern Preventive Medicine. 2012; 39:817–8.
32. Zhang XG, Li XF, Wang SL, Xu XP, Wu JX, Jiao X. Epidemiologic survey of diabetes in Beijing army retired cadres. Chin J Health Care Med,. 2011; 13:50–1.
33. Gao X, Wu XG, Tang Z, Gao F. The epidemiological investigation of the senior citizens in Xuanwu community of Beijing. Chinese Journal of Health Laboratory Technology. 2011; 21:723–4.
34. Li J, Zhang YJ, Sun Y. Investigate and Analysis on Prevalence of Chronic Diseases in Sunhe Community of Beijing. Chinese Journal of General Practice. 2011; 9:600–1.
35. Wang Y, Zhang Y. Hypertension, diabetes mellitus, and cognition of rural residents in Beijing City. Chin J Public Health. 2011; 27:787–8.
36. Li SS, Ma J, Song Y, Li Y, Zong ST, Xiao F, Chen BW, Song M, Lv YP, Liang W. A cohort study on health status among typical community residents in Beijing. Chin J Public Health. 2008; 24:907–8.
37. Xing W. Study on the prevalence of major chronic disease of residents in Baishan Town of Changping District of Beijing City. Occup and Health. 2008; 24:2041–2.
38. Wu BH, Zhou SF, Nie XQ, Li Y. The prevalence and influcencing factors of chronic non-communicable disease aming rural residents in Daxing Dsitrict of Beijing City. Health Education in China. 2008; 24:825–8.
39. Bao C. A comparative analysis of the current status and changing trend of diabetes mellitus in Xicheng District in 2001 and 2007 in Beijing. Chin J Nat Med. 2008; 10:442–6.
40. Jia J. Analysis of the prevalence of diabetes in Haidian District, Beijing. Modern Preventive Medicine. 2007; 34:3869–71.

41. Chen GR, Qu X. Study on risk factors of diabetes mellitus among staff of town government and village committee in Tongzhou District of Beijing. Chinese Journal of Health Education. 2007; 23:686–8.
42. Yang L, Lu L. Dynamic Analysis on Prevalence of Hypertension, Diabetes and Hyperlipidemia in Xicheng District, Beijing, 2001- 2007. Chin Prev Med. 2009; 10:339–42.
43. Xing JP, Yang JF, Zhen YP, Ma YH, Zhao L, Yang J. Prevalence of Impaired Glucose Regulation in Beijing Rural Population and the Necessity to Carrying out Oral Glucose Tolerance Test. Journal of Capital Medical University. 2009; 30.
44. Yang H. Epidemiological feature on diabetes mellitus of countryside in Huairou District of Beijing. China Medical Herald. 2009; 6:125–6.
45. You K, Li YT, Zhang Y, Zhao H. Surveillance of non-communicable chronic diseases and its major risk factors among adults in Shunyi District , Beijing in 2008. Capital Journal of Public Health. 2009; 3:202–6.
46. Chu LN, Feng CR, Feng SH, Zhou HL, Bao CH, Wang Q. Investigation of hypertension, hyperlipidemia and diabetes prevalence in Xicheng District community residents in Beijing City. 2005; Chin J Prec Contr Chron Non-commun Dis: 183-4.
47. Wei Y. Investigation and Analysis on the hypertension, hyperlipidemia and diabetes in the rural women in Tongzhou District Practical Journal of Cardiovascular, Cerebrovascular, and Pulmonary Vascular Disease. 2011; 19:1677–8.
48. Xing Y. Investigation on current status of chronic diseases prevalence among college staff and related preventive measures. Modern Preventive Medicine. 2009; 36:1115–6.
49. Pan CY, Lu JM, Tian H, Kong XT, Lu XP, Yao C, Jiang CE, Deng XX, Wang SY, Zhang XL, Wang ZS, Cui L. Study of the prevalence of diabetes mellitus in adults in the Shougang Corporation in Beijing. Diabet Med. 1996; 13:663–8.
50. Sun YO, Liu HX, Yang YC, Fu CL, Liang R. Prevalence of diabetes mellitus and risk factors in aged cadres. Chin J Prev Contr Chron Non-commun Dis. 2003; 11:233–4.
51. Bao CH, Feng CR, Zhou H. Study on Prevalence of Chronic Disease in Xicheng District. Modern Preventive Medicine. 2005; 32:234–6.
52. Chen ML, Tian H, Pan CY, Lu JM, Yang S. The prevalence of diabetes mellitus and its impact factors in elder cadres. Acad J PLA Postgrad Med Sch□1999; 20:151–3.
53. Shen SL, Xue H, Jiang J, Zhou M. Analysis of diabetic incidence in the middle and elder age group for ten years. Acad J PLA Postgrad Med Sch□2000; 21:108–10.
54. Yang Z, Yu PL, Fan QY, Wang BZ, Gao FK, Zheng H, Tong ZF, Tang L, Shi XH, Sun Y. Research of diabetes and IGT prevalences in middle and old age population in Beijing Geriatr Health Care. 2001; 7:142–4.
55. Guo QY, Yao HB, Wang YZ, Wang YB, Yin YC, Huang H. Analysis of prevalence rate and relative factors of diabetes in middle and old aged males. Clinical Medicine of China. 2002; 18:706–7.
56. Wang SS, Zhang ZF, Shi XY, Ma XB, Zhang BH, Wang H. A Survey of Diabetes Prevalence in the Government Officers Over 40 Years of Age. Journal of Navy General Hospital. 2002; 15:78–80.
57. Zhang XL, Jiang X, Wang J. Investigation on the prevalence of diabetes in the Qinan community, Beijing. Chinese General Practice. 2001; 4:375–6.
58. Zhou ZQ, Sun XC, Jing XS, Qing Y. A cadre of hypertension and diabetes prevalence and relation analysis. Journal of PLA. 1999; 2:38–9.
59. Liu C. Chronic Diseases and Risk Factors among the Local Residents in Huairou District of Beijing. Occup and Health. 2008; 24:106–9.
60. Kuang MX, Zhang S, Lu XP, Li Y. Investigation on the prevalence of diabetes mellitus in Shougang Machinery Factory. The Medical Journal of Industrial Enterprise. 1999; 12:93–4.
61. Zhen YC, Luo SP, Liu BZ, He Q, Wei YR, Tan LL, Sun H, Qu L, Sun T. Investigation on the prevalence of diabetes mellitus among the elderly in Shijingshan District, Beijing. Chin J Prev Contr Chron Non-commun Dis. 1999; 7:41–2.
62. Liu ZY, Xu XS, Li YQ, Jin SX, Feng JG, Ding X. Non-insulin-dependent Diabetes Mellitus in Rich Farmers. Chinese Journal of Prevention and Control of Chronic Non-Communicable Diseases. 1997; 5:215–7.
63. Li ZJ, Zhang PL, Yang C. A comparative study of the prevalence of elder diabetes mellitus in urban and rural areas in Beijing. Chin J Geriatr. 1996; 15:336–8.
64. Li NH, Yang CY, He HD, Song ZN, Zhang P. Investigation on the prevalence of diabetes in elderly people in Beijing. Chinese Journal of Public Health. 1990; 9:249–50.
65. Chen YN, Han X. The Prevalence and Control of Several Common Chronic Diseases among the Adults in Airport District of Beijing in 2005. Chin J Prev Contr Chron Non-commun Dis. 2008; 16:304–6.
66. Wang R. An Investigate Analysis of the Diabetes and Hypertension in the Group of the Mid-age and Old People in the Hepingli- midstreet Community. Chinese Primary Health Care. 2008; 22:48–50.
67. Jia J. Investigation on the prevalence of chronic diseases in elderly people in Haidian District, Beijing. Journal of Chinese Geriatric Medicine. 2008; 28:381–2.
68. Wang WX, Qu ZJ, Zhu A. Epidemiologic snalysis of diabetes among 10932 common residents in Chongqing communities. Chongqing Medicine. 2013; 42:3149–50.
69. Li XB, Shen Y. Analysis on the risk factors of chronic diseases in Fengdu County in 2011. Lab Med Clin. 2013; 10:615–7.
70. Fu J, Liu GQ, Hu X. The investigation of the elderly chronic disease status and the related risk factors in Xuanhua community of Yongchuan area in Chongqing and the exploration of corresponding social intervention measures. Modern Preventive Medicine. 2013; 40:858–60.
71. Sun BL, Que D, Li YY, Duan F, Zhang L. Analysis on the risk factors of diabetes mellitus in community residents in Chongqing. Journal of Community Medicine. 2011; 9:46–8.
72. Xiang CM, Dai Y, M J. Epidemiological investigation of diabetes in a university employee in 2011. Modern Medicine and Health. 2011; 27:2871–2.

73. Liu DM, Wang YH, Li TY, Zhao Y. Attack rate of diabetes mellitus is significantly high in adults ,born in 196(intermediate stage of famine times). Journal of Chongqing Medical University. 2009; 34:1712–4.
74. Liu XF, Liu DW, Yang XL, Zhang HD, Xiang XZ, Zhao J, Sun C. Distribution and Relative Influencing Factors of Diabetes Mellitus in Chongqing. J Prev Med Inf. 2007; 23:158–60.
75. Liao H. Analysis of fasting blood glucose and urine glucose in different occupation groups of 940 cases. Lab Med Clin. 2007; 4:277–9.
76. Tang XJ, Lu XN, Li G, Zhang SH, Ma GC, Xu X. The study of diabetes and related factors on population of Chongqing certain University. Chongqing Medicine. 2009; 38:1281–2.
77. Tang XJ, Zhang SH, Li G, Lu XN, Ma GC. Prevalence study on type 2 diabetes mellitus in communities of Chongqing. Chinese Journal of Clinical Rehabilitation. 2006; 10:10–2.
78. Luo FS, Ran CR, Chen T, Lu Y. Epidemiological survey of elderly diabetes mellitus in Beibei District of Chongqing City. Sichuan Medical Journal. 2002; 23:980–.
79. Cai LX, Lin MZ, Zhang ZP, He J. Prevalence and risk factors of diabetes mellitus in community residents in Xiamen. Chin J Prev Contr Chron Dis. 2014; 22:710–2.
80. Chen LF, Ma H, Chen XY, Zhang HJ, Lai Q. Investigation of prevalence rates of diabetes mellitus and IFG among elderly residents in Xiamen Kaiyuan Community. J Med Theor & Prac. 2014; 27:2410–3.
81. Xu SY, Ming J, Gao B, Wan Y, Yang CB, Chen G, Ji QH. Comparison of diabetes (pre-daibetes) prevalence and awareness between province Shanxi and Fujian. Chongqing Medicine. 2013; 42:2765–8.
82. Li HS, Qiu XQ, Pan W, Guan YH, Peng Y. Survey on chronic disease and related behavior risk factors among citizens in Yongan city. Chronic Pathemathol J. 2013; 14:584–6.
83. Li H, Mu Y. The Prevalence of Type 2 Diabetes Mellitus and its Risk Factors in the Elderly in Gushan Town, Fuzhou City. Chin J Prev Contr Chron Dis. 2009; 17:169–71.
84. Zhu TB, Jin X. Investigation and analysis of five kinds of diseases in retired old age education workers in Huaqiao University. Fujian Medical Journal. 2002; 24:90–.
85. Wang QH, Chen L, Liu J. Investigation of obesity, hypertension, and diabetes prevalence in the Gulangyu Islet area. Chin Prev Med. 2003; 4:57–8.
86. Provience DCGoF. Comparative report of two investigations for diabetes in Fujian Province. J of Fujian Medical University. 1997; 31:229–31.
87. Yang FQ, Xu WZ, Yang SJ, Wang AD, He P. A survey of 2600 diabetic people in Putian area. Strait Journal of Preventive Medicine. 1996; 2:17–9.
88. Wang W, Wang HB, Hua W, Yao C. Analysis of epidemiological survey of diabetes residents in Kongtong District of Pingliang. Bull Dis Control Prev. 2014; 29:11–5.
89. Zhao LL, Wang YN, Liu X. Epidemiological survey on diabetes among the comunity residents in Lanzhou. Modern Preventive Medicine. 2014; 41:2500–2.
90. Wei ZQ, Zhang R, Zhang S. The analysis on prevalence and awareness rates of hypertension and diabetes in Chengguan Dsitrict of Lanzhou Chinese Primary Health Care. 2013; 27:74–6.
91. Han CM, Zhang JH, Cui Y. Analysis of the prevalence of diabetes mellitus and related risk factors in the health examination of employees in a power supply enterprise. Chin J Lab Diagn. 2014; 18:1200–2.
92. Yang CQ, Liu J, Qiu JW, Xi ZG, Sun J, Liang YH, Wang LY, Cai FY, Zhou XL, Liu Z. Epidemiological investigation of diabetes and impaired glucose regulation of adults in Tianzhu Tibetan Autonomous Country, Gansu province. Chin J Diabetes. 2013; 21:499–501.
93. Zhu SP, Zhang HH, Yu GW, Niu J. The epidemic characteristics of overweight and obesity and relation with prevalence of diabetes in Gansu province. Chin J of Public Health Eng. 2013; 12:98–100.
94. Liu LJ, Ma YP, Bai Y, Zhao F. A survey of morbidity of diabetes mellitus on middle-aged and patients of Yugur Nationality in Gansu province. Journal of Northwest University for Nationalities(Natural Science). 2013; 34:70–3.
95. Yue X, Li JS, Shen XP, Liu XN, Yang Y. The investigation and analysis on the prevalence status of diabetes mellitus among lanzhou residents. Modern Preventive Medicine. 2010; 37:3819–20.
96. Chen YD, Li C. A survey of adult hypertension and diabetes in Qingcheng County of 2012 Bull Dis Control Prev. 2013; 28:65–6.
97. Wang Q. Analysis of the baseline for hypertension and diabetes in Gaolan County, Gansu Province. Bull Dis Control Prev. 2013; 28:67–74.
98. Li XX, Wang X. Analysis on the physical exam ination results of 422 aged people in Yugu Nationality Autonomous County of Gansu Province. Chin J Nat Med. 2010; 12:364–6.
99. Wang X. Investigation of fasting blood glucose in healthy people in Zhangye. Health Vocational Education. 2011; 29:112–3.
100. He YJ, Lin Y. A survey of current status of diabetes in Jiangong Mid-street community of Small West Lake street in Qilihe District, Lanzhou city Health Vocational Education. 2003; 21:122–3.
101. Gao L, Li MX, Fu W, Li T, Li QW, Quan J. Epidemiological investigation of diabetes and impaired glucose tolerance in elderly people in Lanzhou. Journal of Lanzhou Medical College. 1998; 24:41–4.
102. Wang H. Diabetes Prevalence Analysis on Power System Staff. Medical Information. 2013; 26:495–.
103. Cheng K, Long L, Huang JP, Dan S. Investigation and Analysis on the fasting blood glucose level and the high blood glucose detection rate in the population of Tongren City. J Disease Monitor & Control. 2014; 8:164–5.
104. Zhang JP, Wang Y, Liu LY, Zhang W, Wu YD, Li PH, Yan J. Nutrition and health status among residents of Yunyan District, Guiyang city in 2010. Chin J Public Health. 2013; 29:1212–4.
105. Luo KJ, Yin L, Cao Y. Investigation of 3807 cases of diabetes mellitus in physical examination. Guizhou Medicial Journal. 2007; 31:279–80.
106. Province DGoKiG. Investigation report on the prevalence of diabetes in 523 patients with diabetes in Kaili County, Guizhou

Province. Journal of Guiyang Medical University. 1981; 6:78–83.

107. Guo JX, Zhu KX, Zhuang XX, Gan B, Yu HB, Pan JY, Zhang Z, Wang G. Study on the epidemiological characteristics and risk factors of diabetes among residents (≥15 years old) in Haizhu district of Guangzhou in 2010. Chin J Prev Contr Chron Dis. 2014; 22:697–700.

108. Wu QJ, Liang XM, Zhou ZH, Lin CH, Zheng WW, Wang X, Lu XR, Chen X. Survey of status quo of diabetes prevalence of commnuity residents and its influence factors in Guangzhou city. Chinese Nursing Research. 2014; 28:1594–6.

109. Li K, Lin GZ, Pan BY, Zhou Q, Du L. Investigation of hypertension, diabetes mellitus, and related risk factors in the 15-69 years old in Guangzhou city in 2008. Chin J Prev Contr Chron Dis. 2013; 21:720–2.

110. Cao Y, Xue YM, Li CZ, Zhang ML, Gao F, Xie CH, Zeng HY, Luo XR, Li JM, Fu X. Epidemiological investigation of diabetes and prediabetes in community residents in the suburbs of Guangzhou. J SouthMed Univ. 2010; 30:2122–4.

111. Zhang Q. The amalysis of 43384 civil diabetes, dyshipidemia and hypertension's prevalence. Knowledge of prevention and treatment of cardiovascular disease (Academic Edition). 2012; 18–9.

112. Pan BY, Liu WJ, Luo BF, Du L, Wang JH, Chen J, Wei Y. The epidemiological characteristics and evaluation on prevention of diabetes mellitus in Guangzhou city. Chin J Dis Control Prev. 2006; 10:489–92.

113. Liu Y. Investigation on the health status of Whampoa Street Community in Guangzhou City. Journal of Community Medicine. 2008; 6:12–4.

114. Zhu KX, Luan YM, Wu HX, Pan JY, Huang PZ, Liu X. Study on the Prevalence Rates of Diabetes and its Risk Factors among Residents in Haizhu District, Guangzhou. Chin J Prev Contr Chron Non-commun Dis. 2007; 15:561–4.

115. Hong JX, Zhang YF, Zhou XY, Huang MG, Zhong YL, Liu Y. Investigation of main chronic diseases in community residents in Luogang District. Strait Journal of Preventive Medicine. 2007; 13:36–7.

116. Fan JW, Zhang WF, Zhang RD, Yu Y, Luo S. Prevalence of diabetes mellitus and its influencing factors in Liwan District, Guangzhou. South China J Prev Med. 2011; 37:30–2.

117. Chen YL, Wang J. The national survey of diabetes epidemiology in Baini community, Sanshui District (Longevity village) Journal of Gannan Medical University. 2011; 31:587–9.

118. Wu XH, Xiao XW, Ye GL, Kang HX, Lu ZJ, Zhang MN, zhou H. Prevalence of hypertension and diabetes, status of knowledge, attitude and practice among residents in Sshaoguan city. J Trop Med. 2014; 14:1339–42.

119. Guo YF, Liu Z, Zhang Z, Huang BZ, Zhou HB, Lei L, Peng J. Analysis of prevalence and risk factors of type 2 diabetes in Baoan District, Shenzhen. Chin J Prev Contr Chron Dis. 2014; 22:454–6.

120. Zhao Y, Wang HL, Lan Y. Analysis on the leve l of plasma glucose among the public officers in Longgang District. China Modern Medicine. 2013; 20:165–6.

121. Liu SY, Xu S, Chen ZW, Wang CY, Yang J. Survey on the epidemic and related awareness rate of chronic disease of residents in Nanshan district of Shenzhen. China Modern Medicine. 2012; 19:157–8.

122. Chen YF, Shi JP, Wu JZ, He W. Analysis of health examination results and health education of a bank employee. Journal of Qilu Nursing. 2011; 17:26–7.

123. Deng JG, Wang TQ, ZH Li, Fan B, Li YH, Huang Y. A survey of diabetes pr evalence in adults of Gongming community in Shengzhen. Chinese Medical Engineering. 2008; 16:135–7.

124. Chen J. The status of the elderly with chronic illness analysis. Practical Journal of Cardiovascular, Cerebrovascular, and Pulmonary Vascular Disease. 2009; 17:596–7.

125. Chen YH, Zeng YQ, Tan J. Analysis on prevention and control of diabetes mellitus among elderly people in Fengcheng community of Qingyuan city. Occup and Health. 2014; 30:548–50.

126. L CS i, Huang DH, Zhou ZS, Zhu Y. Epidemiological investigation of diabetes in rural area of Qinxin District of Qingyuan City. Occup and Health. 2014; 30:658–60.

127. Hao S. An investigation of the current status of university staff chronic disease and risk factors. Journal of Zhaoqing University. 2013; 34:98–100.

128. Zhong WQ, Lu SY, Lin S. Investigation on the prevalence of diabetes mellitus and related health knowledge of residents in Zhaoqing city in 2012. South China J Prev Med. 2013; 39:75–7.

129. Wang DC, R HuL, Chen WP, Liao YT, Chen QX, Chen JR, Li HY, Li X, Lin Y. Survey on prevalence of the chronic disease among the middle and old aged women in an urban community of Zhanjiang City. Pre Med Trib. 2013; 19:325–7.

130. Tang YX, Zhou LF, Zhao G. An epidemiological survey of diabetes of Zhuang and Yao nationalities in Lianshan area, Guangdong province. China modern Doctor. 2010; 48:84–5.

131. Fen JD g, Lu XC, Guo HQ, Huang W. Prevalence study on health condition of middle and old aged community population in Dongguan city. National Medical Frontiers of China. 2010; 5:92–3.

132. Chen B, Li DY, Liang XD, Li Y. Prevalence of Diabetes and Its Inf luencing Factors Among Residents Aged 15-69 Years in Zhuhai. Pract ical Prevent ive Medicine. 2011; 18:1175–7.

133. Jin LZ, Wu WM, Ma YD, Chen J, Xiao HK, Chen X. Prevalence and risk factor analysis of diabetes in Doumen rural area of Zhuhai City. South China J Prev Med. 2009; 35:9–13.

134. Li J, Li DF, Guo WC, Li X. Survey incidence of chronic disease in retired cardres in Zhongshan City. China Tropical Medicine. 2009; 9:2209–10.

135. Zhao G, Tang YX, Zhou L. Guangdong Province Caroline Zhuang and Yao Nationalities Region Diabetes Epidemiological Investigation. Chinese Primary Health Care. 2008; 22:60–2.

136. Xia BW, Gao MF, Peng WJ, Yao AY, Jin DN, Liu L. Epidemiological study of type 2 diabetes in elderly people in Zengcheng. J Mod Clin Med Bioeng. 2006; 12:199–200.

137. Shao H, Li SG, Qiu XM, Chen XM, Zhang GX, Zhou S. Baseline survey of hypertension and diabetes in elderly patients in Foshan City. Chin J Geriatr. 2005; 24:705–6.

138. Deng HR, He W. Survey on diabetes and its related factors in rural community. Journal of Nurses Training. 2005; 20:1130–1.

139. Chen JH, Li K. Survey of risk factors for diabetes in 4083 communities and community nursing intervention. Chinese Primary Health Care. 2004; 18:57–8.

140. Chen SR, Xu HX, Chen LX, Chen LS, Yang YH, Zheng AY, Chen WF, Ji WY, Ni WF, Hong S. Investigation and analysis to apatients with diabetes mellitus in Shantou special economical zone. Chinese Journal of Clinical Rehabilitation. 2004; 8:1001–3.

141. Pan BY, Qiu XL, Fang F, Luo BF, Liu WJ, Chen J, Wang J. The prevalence and risk factors of diabetes mellitus in residents of dongshan and yuexiu district of guangzhou. Modern Preventive Medicine. 2005; 32:516–7.

142. Sun L, Ren X. Results of physical examination for workers and staff: analysis of the examination results in 4583 persons. Chinese Journal of Health Education. 2005; 21:115–6.

143. Liu WX, Yu FL, Ding CP, Zhou JY, Wang XJ, Li L, Shang Y, Zhang BF, Yu JG, Ouyang D. Investigation on the prevalence of diabetes mellitus in the railway authorities in Guangzhou. Guangdong Medical Journal. 2000; 21:1055–6.

144. Peng J, Zhou H, Cheng JQ, Li YP, Zhou J. Characteristic Comparison Between the Subjects with Diabetes and IGT in Shenzhen Special Economic Zone. Practi cal Preven tive Medicine. 2001; 8:389–90.

145. Han QS, Wang MF, Huang QF, Lin XL. Analysis of the staff's health and related influence factors in a hospital in Shenzhen. Chinese Journal of Health Education. 2008; 24:212–3.

146. province DeicGoG. A cross-sectional study on diabetes mellitus in Guangdong Province. Guangdong Medical Journal. 2011; 22:455–8.

147. Han LM, Zeng YY, Zhang RH, Feng DX, Gu J. Prevalence rate of diabetes mellitus in developed rural areas. Chin J Contr Chron Non-commun Dis. 2002; 10:87–8.

148. Liu ZY, Cheng JQ, Peng J, Zhou H, Luo BR, Chi H. Risk factors of type 2 diabetes in Shenzhen. Chin J Prev Med. 2000; 34:232–4.

149. He JQ, Ye GQ, Peng H, He G. Analysis of diabetes epidemiology in Gaozhou City. Guangdong Journal of Health and Epidemic Prevention. 1999; 25:35–6.

150. Li LJ, Yin Y, Huang QY, Huang X. Investigation of common chronic diseases and related risk factors in the elderly in community. Chinese Journal of Gerontology. 2008; 28:169–70.

151. Yang YH, Chen SR, Lin S. 150 cadres diabetes survey report. Journal of Shantou University Medical College. 1998; 11:24–5.

152. Wang SZ, Zhang SB, Zhang SD, Dong BH, Xie M. The prevalence of diabetes mellitus and islet function in middle-aged and elderly cadres. Guangzhou Medicine. 1988: 15-7.

153. Yan T, Huang BJ, Yu BJ, Fu MC, Li HH, Liu JL, Hu GL, Wang Z. Prevalence of diabetes mellitus in Guangzhou (Canton) District. ACTA Academiae Medicinae Zhong Shan. 1981; 2:658–69.

154. Tan XW, Li L, Huang KM, Zheng ML, Liu J, He XN, Lai Y. The change tendency of prevalences for impaired fasting glucose and diabetes in 106260 population undergoing health examination in Guangzhou during 2002–2005 Chin J Diabetes. 2008; 16:169–71.

155. Xia SL, Zhou H, Wang T, Zhang RH, Wu HD, Wu L. Prevalence and Risk Factors Associated with Non-Communicable Chronic Disease in Zhongshan. Journal of Tropical Medicine. 2008; 8:77–9.

156. Chen YZ, Luo ZB, Deng ZM, Huang YZ, Lin JY, Lu QM, Mo BQ, Chen Z. Monitoring report on health status of residents in Binyang County of Guangxi Province in 2012. Applied Prev Med. 2013; 19:300–2.

157. Deng XM, Tang LL, Lu B. Epidemiological investigation and analysis of diabetes mellitus in health examination in Nanning City. Journal of Guangxi University of Chinese Medicine. 2013; 16:38–40.

158. Xu YF, Li P, Zhou J. Epidemiological survey of chronic diseases in some residents of Nanning City. Applied Prev Med. 2007; 13:85–7.

159. Gan Z. Analysis of diabetes mellitus in 953 elderly residents. Journal of Community Medicine. 2010; 8:71–2.

160. Xu YF, Lin XQ, Li P, Guo LF, Zhou J. Analysis of prevalence status of diabetes and its main influencing factors of urban residents in nanning. Modern Preventive Medicine. 2008; 35:2499–502.

161. Lu JX, Liang Y, Li TZ, Li JD, Zhou LQ, Lu YJ, Huang S. Survey of hypertension, daibetes mellitus and obesity of Zhuang ethnic group in qintang district in Guiyang City. China Medicine and Pharmacy. 2012; 2:11–2.

162. Li HX, Huang ZQ, Li TZ, Lu KX, Pan XS, Wei L. Survey on the Prevalence of Hypertension,Obesity and Diabetes Among Residents in Jingxi County,Guangxi. Guangxi Medical Journal. 2012; 34:393–5.

163. Wang DF, Chen G. Nursing Intervention and Analysis of Risk Factor on Type 2 Diabetes Patients of Workers inWestern Guangdong. Journal of Clinical Nursing. 2010; 9:9–11.

164. Huang Q, Xiao YF, Wang L. Investigation on the health status and health education of the staff in a school. Chin J School Doctor. 2010; 24:138–9.

165. Liu X. Analysis of prevalence rate and related factors of diabetes mellitus. Guide of China Medicine. 2012; 10:562–3.

166. Li SM, Zhang YS, Lv YP, Yang PJ, Huang H, Lu W. Survey on Diabetes, Prevalence of Impaired Glucose Regulation, and Nutrition Research in Qinzhou Practical Preventive Medicine. 2010; 17:1476–8.

167. Pang SH, Huang DT, He C, Wang G, Wang XP, Zhong LX, Zhu YQ, Li CF, Zhou QH, Pang JB, Zeng B. Analysis of diabetes mellitus prevalence rate and risk factors of village people in bobai county of Guangxi. Internal Medicine of China. 2010; 5:353–5.

168. Chen SP, Huang P. Analysis of health examination results of 1746 cases of community population. Journal of Community Medicine. 2010; 8:62–3.

169. Huang MW, Nong MG, Huang HQ, Peng J, Lu XQ, Liang W, Li T. Relationship between risk factors and the attack of diabetes mellitus in the middle-old age men in Bose communities. Journal of Youjiang Medical College For Nationalities. 2003: 22–5.

170. Yao AN, Peng YH, Chen QW, Luo QP, Su FQ, Li SL, Zhou CP, Xie H, Chen Q. Prevalence and Risk Factors Analysis of

Diabetes in Hezhou,Guangxi. Guangxi Medical Journal. 2011; 33:1243–6.

171. Tang ZZ, Chen XL, Han YB, Huang ZY, Huang L, Ruan Q, Chen L, Fang ZF, Yang J, Zhang JH, Chen Q. The study on nutrition and health status of residents in Guangxi, 2002. Guangxi Prev Med. 2005; 11:257–63.
172. Zhou SZ, Xiao CQ, Ning X. Study on the characteristics of diabetes mellitus in different occupational groups in the community. Journal of Guangxi Medical University. 2000; 17:49–50.
173. Feng L. Epidemiological investigation and analysis of diabetes mellitus. Chinese Medicine of Factory and Mine. 1999: 74–5.
174. Lv XZ, Chen GJ, Fu YM, Qu PJ, Chen LF, (Yulin DM Investigation Group G. Epidemiological investigation of diabetes mellitus and impaired glucose tolerance in a population of 10763 persons in Guangxi rural economic reform area. ACTA Academiae Medicinae Guangxi. 1995; 12:317–21.
175. Do GX. Investigation report on the 20295 population of the sugar producing area and the non sugar production in Guangxi. Guangxi Medicine. 1983; 5:140–2.
176. Fu ZW, Wu J, Wang H. Analysis of epidemiological status and risk factors of diabetes mellitus among floating population in Hainan Province. China Tropical Medicine. 2014; 14:1192–6.
177. Zhu CY, Wang LP, Lin YB, Zhou X. Analysis of results of two-year health check-up of functionaries. China Tropical Medicine. 2007; 7:1256,65.
178. Nie D. Results of health check-up of medical workers. China Tropical Medicine. 2009; 9:589,02.
179. An SX, Guo XR, Zhang AY, Chen C. Investigation and nursing intervention on the incidence of diabetes mellitus. Nursing Practice and Research. 2013; 10:148–9.
180. An SX, Guo XR, Chen XQ, Zhang A. Investigation on the related factors of diabetes in 8 townships in Zanhuang County. Journal of Guiyang College of Traditional Chinese Medicine. 2014; 36:30–2.
181. Chen FG, Zhao CA, Feng DY, Gao WL, Li B, Xu BH, Zhou JK, Guo Z. Investigation of diabetes prevalence and related knowledge in Shijiazhuang city in 2010. Heibei Mdeical Journal. 2014; 36:3170–1.
182. Ma R. Prevalence survey of diabetes mellitus in 5678 adult healthy people in Shijiazhuang. Clinical Misdiagnosis & Mistherapy. 2007; 20:14–5.
183. Guo Z. Investigation on the prevalence of diabetes in rural areas of Luancheng County. China J of Clinical Rational Drug Use. 2014; 7:84–5.
184. Liu YQ, Wu SL, Xing AJ, Liu JQ, Jin C, Zhou Y. Trend analysis of the prevalence and awareness rate of diabetes mellitus. Hebei Medical Journal. 2014; 36:2684–5.
185. Zhang QL, Yao L. Prevalence and risk factors of chronic disease among residents over 45 years in Fengnan District. Occup and Health. 2014; 30:252–4.
186. Wang BH, Zhang JW, Tang MG, Bai J, Pu JY, Li Y, Zhou Y, Song G. Prevalence and influencing factors of diabetes in Jidong community of Tangshan City Chin J Evid Based Cardiovasc Med. 2013; 5:247–8.
187. Zhang ZK, Liu D, Zhang XH, Xiang D, He JK, Zhan SWG, Gao QH, Li F. A survey of chronic diseases among urban and rural residents aged from 79 to 30 in Tangshan City. Modern Preventive Medicine. 2012; 39:6278–80.
188. Li WH, Zhang HM, Xin J. Epidemiological survey of diabetes mellitus among Tangshan residents. Modern Preventive Medicine. 2012; 39:4373–.
189. Wu QW, Xing FM, Dong SL, Li J. Analysis of the current situation of the prevalence of chronic disease in older workers in Kailuan Group. Chinese Journal of Gerontology. 2009; 29:870–2.
190. Zhang H, Zhang W, Gao SJ, Jiang N, Li YT, Wang X. Analysis and intervention stragety of blood lipids and glucose levels in 1363 cases og Hebei province's civil servants over 40. Int J Lab Med 2014; 35:1588–92.
191. Yang G. Prevalence and awareness of hypertension and diabetes among peasants in Yutian County of Hebei Province. Occup and Health. 2014; 30:346–8.
192. Zheng X. The prevalence of impaired fasting glucose and diabetes situation of Longhua County cadres and workers. Heibei Medicine. 2010; 16:381–2.
193. Kang KY, Liu XS, Zhang HB, Wan XO g, Sun XY, Ren L, Liu S. Investigation on Relationship between hypertension,diabetes and obesity among residents in Beidaihe District of Qinhuangdao City. Occup and Health. 2012; 28:702–4.
194. Zhu JQ, Li H, Zhang JX, Zhang JY, Sun JX, Xue YF, Wang LN, Ren HY, Fang HY, Yang X. Analysis on Epidemiological Character istics and its Related Risk Factor s of Chronic Diseases among Adult Residents in Hebei Province. Chin J Prev Contr Chron Non-commun Dis. 2007; 15:539–43.
195. Liu YR, Fu ZK, Li HQ, Liu L, An L, Zhang L. Investigation and current status of diabetes mellitus in Baoding City. Medical Journal of Chinese People's Health. 2007; 19:831,3.
196. Li JH, Shi BL, Yu YQ, Qi WJ, Tian L, Shi S. Investigation on the prevalence of type 2 diabetes in people over 40 years old in Zhangjiakou. Journal of Hebei North University(Medical Edition). 2007; 24:48–9.
197. Yang JF, Liu WJ, Yan FH, Xue H. Investigation and Analysis on the prevalence of fasting blood glucose and diabetes in a professional group in Handan. Lab Med Clin. 2009; 6:358–9.
198. Wang YF, Peng H. Epidemiological survey of diabetes mellitus in rural areas of Xianxian County Province, Hebei Province. Modern Journal of Integrated Traditional Chinese and Western Medicine. 2009; 18:1831–2.
199. ZhangCG, Z WuH, Gao H. A Correlation Study of the Abdominal Obesity and Diabetes in 3 753 of Civil Servants in Hebei Province. Chin J Prev Contr Chron Dis. 2011; 19:255–6.
200. Wang ZF, Lu BT, Tian JG, Liu SF, Zhang GX, Wang JG, Tian XQ, Liu JM, Zheng TR, Guo S. Investigation and Analysis on nutrition and health status of residents in Tanghai County. Chinese General Practice. 2004; 7:983–4.
201. Wang SF, Chen C. Investigation on the Department of internal medicine disease prevalence of staff and workers. Lit & Inf Prev Med. 1999; 5:16–7.
202. Jiang X, Zhang XM, Dong J, Pei X. Relationship between obesity and diabetes prevalence in Hebei Province. Chin J Prev Contr Chron Non-commun Dis. 1999; 7:146.
203. Wang X, Gao BP, Zhang JG, Sun TY, Li SZ, Li ST, Gao MG, Jiao XY, Li WC, Pan PP, Gao JH, Zou S. Diabetes survey report

for workers in iron and steel enterprises. Chinese Medical Journal of Metallurgical Industry. 1994; 11:181–2.
204. Cao L, Shao TT, Guo J. Epidemiological investigation of diabetes in Xiping County. Chin J Mod Drug Appl. 2014; 8:241–2.
205. Li YQ, Fen TP g, Zhao JZ, Yin L, Li L. Study on the prevalence and the influencing factors for type 2 diabetes mellitus ampng urban and rural populations in Henan. Modern Preventive Medicine. 2014; 41:2899–901.
206. Gao L, Zhou G, Feng SX, Feng HF, Han B, Wang C. Epidemiological investigation of diabetes among residents in Henan. Journal of Zhengzhou University (Medical Sciences). 2014; 49:123–6.
207. Cai Q, Sh SH i, Wang Z, Wang XC, Huang J, Li W. The status analysis of type 2 diabetes of over 60 years old in Xinzheng regin. Henan Medical Research. 2013; 22:298–301.
208. Liu Y, Huang RP, Han P, J ZhangW, Yin FK, Wang S. Study on risk factors of type 2 diabetes among residents from a community of Zhengzhou city. J Medical Forum. 2012; 33:74–6.
209. Sun YJ, Ban LW, Zhao S, Ya Li, Mingyu Zhu, Pingping Chen, Xie D. Prevalence of diabetes and its related factors among college faculty. Chin J Public Health. 2012; 28:751–3.
210. Hou YQ, Shi S. Investigation on prevalence of diabetes among teachers at acertain university. Health Medicine Research and Practice. 2012; 9:54–8.
211. Li QH, Yuan XJ, Wang H. Incidence investigation of diabetes mellitus in Hui and Han population of Jiaozuo region. Clinical Focus. 2012; 27:1847–9.
212. Wang ML, Zhang B, Zhang J. Physical condition and investigation of health examination in the 800 service staff. Today Nurse. 2012: 167,33.
213. Ma YQ, Dong SZ, Yang L. Prevalence survey of diabetes on humans of Pingdingshanmineral Area of Henan province in 2009. Journal of Medical Forum. 2011; 32:75–9.
214. Liu Y. Analysis of blood glucose levels of faculty and staf f of Henan University. Chin J School Doctor. 2012; 26:122–5.
215. Yang LM, Zhou J, Wang CJ, Pan JJ, Shen MH, Hu D. The secular trend of prevalence and risk factors for type 2 diabetes mellitus in the rural Henan population. Journal of Hygiene Reseach. 2010; 39.
216. Cui YX, Zhang B, Zhang Y. The prevalence of diabetes mellitus and hypertension of rural residents in Chen Village, Mianchi County. Henan J Prev Med. 2011; 22:289–90.
217. Li YY, Hu DS, Li CY, Ping ZG, Fu PY, Zhang WD, Zhang MX, Xi Y. Prevalenee and risk factors of diabetesmellitus in rural residents. China J Public Health. 2008; 24:1273–5.
218. Zhang XF, Liu YH, Cui R, Chen Z. Investigation and analysis of the correlation between the incidence of diabetes and age. Experimental & Lab Med. 2008; 26:686.
219. Yang XY, Ru X. Analysis of health examination results of 655 teaching staff in a University of Engineering. Chin J School Doctor. 2009; 23:340–2.
220. Xu DM, Wei Y, Qu H. Prevalence of diabetes mellitus and its risk factors in rural areas of Henan. Chinese Journal of Health Statistics. 2011; 28:582–4.
221. Qiu XC, Cao JL, Wu XR, Liu QW, Cheng WL, Wang WD, Lv J. Survey on the prevalence of chronic non communicable diseases in Jiefang District community of Jiaozuo City. Henan J Prev Med. 2003; 14:146–7.
222. Zhang L, Zhang SF, Hu DS, Shi XZ, Wang P. Nutrition and health status survey in Henan province. Journal of Zhengzhou University (Medical Sciences). 2010; 45:124–8.
223. Jiang W. A survey on the prevalence of chronic diseases in the financial system of Puyang City. Journal of Medical Forum. 2004; 25:9,14.
224. Tian QF, Feng Z. The logistic regression analysis of diabetes mellitus risk factors in natural population of Henan Province. Journal of Zhengzhou University(Medical Sciences). 2002; 37:238–40.
225. Liu GH, Feng ZJ, Fan J. Investigation on the epidemiological characteristics of diabetes mellitus and impaired glucose tolerance in natural poprlation of Henan~ Province. Journal of Zhengzhou University (Medical Sciences). 2002; 37:499–501.
226. Shao TX, Zhang SY, Yang WB, Tang XM, Wang XT, Ma LY, Kang J. Analysis of Health State in 244 Middle-aged and Old-aged Teachers and Staff. Journal of Luoyang Medical College. 2002; 20:212–4.
227. Liu JM, Chen S. The investigation of prevalence and complications of diabetes in Anyang retired cadres. Henan Medical Information. 1998; 6:39–40.
228. Feng RD, Yao FL, Chu R. Investigation of the prevalence of diabetes in some organs in Zhengzhou. Chinese Journal of Endocrinology and Metabolism. 1994; 10:173.
229. Lu YF, You AG, Guo Y, Liu JW, Zhang WD, Zhang MX, Xi YL, Hu D. Prevalence, awareness and treatment of type 2 diabetes mellitus in rural residents of Henan. Journal of Medical Forum. 2008; 29:23–5.
230. Xu L, Zhang W. Study on influencing factors of rural residents health in three provinces of China. Chinese Journal of Health Education. 2013; 29:126–9.
231. Wang J, Sun B, Zhan DF, Lan L, Cui WX, Xu GH, Sui C. Analysis of the factors affecting the early stage of diabetes and diabetes in Harbin City. China J of PHM. 2012; 28:80–1.
232. Yang YJ, Yang XY, Yu ZZ, Ma L, Gao X. Analysis of physical examination results of 30351 elderly people in Ji'nan City. Shandong Medical Journal. 2014; 54:85–6.
233. Sun H, Yuan CS, Liu Y, Liu MN, Wang B. Investigation on type 2 diabetes and obesity in community adults. Chin J Public Health. 2005; 21:478–9.
234. Fu LY, Wang LM, Fan JQ, Xu YR, Wu JL, Hu H. The study on prevalence rate of diabetesmellitus in Dawor race in Qiqihar. Chinese Journal of Practical Internal Medicine. 2005; 25:308–10.
235. Wu XP, Han LM, Hao X. Interrelating investigation of diabetes and hypertension in middle-aged and senile people. Journal of Disability Medicine. 1997; 7:32–3.
236. Wang JP, Jiang YY, Zhang JL, Cao L, Tai Y. The difference between 1997 and 1985 diagnostic criteria to investigate the prevalence of diabetes mellitus. Chinese Journal of Diabetes. 1999; 7:105–6.
237. province DGoH. Epidemiological survey of diabetes mellitus in Heilongjiang Province. 1980: 1–5.

238. Wei X, Wu XP, Chen ZF, Ye HY, Tian Y, Han LM, Cui Z. Investigation of prevalence rate of 1706 middle aged and senile population with diabetes and its correlation factors. Journal of Harbin Medical University. 1995; 29:480–2.
239. Li GY, Zhang YJ, Liu M, Guo Y. Investigation on the Prevalence of Diabetes among the Residents in Daqing City in 2007. Pre Med Trib. 2008; 14:401–2.
240. He X. Analysis of incidence and epidemiology of type 2 diabetes in large community. Chinese Manipulation & Rehabilitation Medicine. 2011: 243.
241. Liu X, Liu X, Cao J. Analysis of incidence and epidemiology of type 2 diabetes mellitus in County Community. Hainan Medical Journal. 2009; 20:156–8.
242. Huang Y, Zhuan JF g, Li X. Investigation on the prevalence of blood glucose and blood lipids and diabetes in elderly people in Changde City. J Bengbu Mes Coll. 2014; 39:385–6.
243. Lian S. Investigation of rural diabetic population in Yuanling County. Jilin Medical Journal. 2010; 31:4314–6.
244. Li L, Chen XX, Lei WY, Cai Y. Analysis of current situation on diabetes mellitus in Hengyang City. Journal of Hunan Enviroment-Biological Polytechnic. 2010; 16:19–22.
245. Su QT, Zhou JM, Xiao D. Investigation of hypertension, hyperlipidemia and diabetes prevalence in Minority Areas. Modern Preventive Medicine. 2003; 30:347–8.
246. Dai JF, Li ZD, Gao JY, Luo FL, Zheng S. Epidemiological investigation of chronic non-communicable diseases in elderly people in Yongzhou City. Pract ical Preventive Medicine. 2004; 11:1262–3.
247. Sha YH, Chen LL, Zhong F, Tang W. Discussion on the characteristics of blood glucose and blood lipid in the minority nationality regions of Xiangxi. Modern Preventive Medicine. 2008; 35:158–9.
248. Duan Y. The prevalence of hypertension, diabetes mellitus and hyperlipemia among 1369 subjects for physical examinationin Changsha. Bull of Hunan Med Univ. 1997; 22:407–10.
249. Wang JM, Chen SG, Huang JK, Dong C. Investigation on the prevalence of diabetes mellitus in 2005 people aged over 25 years in Hengyang City. Journal of Hengyang Medical College. 1997; 25:25–7.
250. Du HL, Wang XH, Li Q. Epidemiological survey of type 2 diabetes mellitus in the elderly in Wuchang vehicle factory. Chinese Journal of Gerontology. 2005; 25:456–7.
251. Liu X, Zhu D. Monitoring and analysis on chronic disease and related risk factors in residents in Xiangzhou District of Xiangyang City. Occup and Health. 2014; 30:2744–6.
252. Zhang SB, Jin JJ, Ma YF, Peng G. Analysis of risk factors in Lengji town residents over the age of 60 with diabetes. Journal of Modern Clinical Medicine. 2014; 40:356–7.
253. Wang Q. Analysis on the changing trends of diabetes mellitus patients among the faculty of a university. Journal of Mathematical Medicine. 2014; 27:423–4.
254. Guo Y, Li F, Sun HL, Yan YQ, Yang NN, Gong J. Prevalence and control of diabetes mellitus among older residents in Wuhan municipality. China J Public Health. 2014; 30:729–31.
255. Zhang ZY, Zhou ES, Yang X. Statistic analysis for the prevalence tendency of diabetes and pre-diabetes in aniversity community. J of Pub Health and Prev Med. 2013; 24:18–20.
256. Jin J, Xiao HJ, Chen Y, Li HB, Xiao JH, Xu Y, Wang M, Hou M. The prevalence of diabetes among resident in Wuhan Dongxihu distric. Medical Journal of Chinese People's Health. 2010; 22:2523–5.
257. Jing YB, Chen XJ, Li H, Luo Q. BMI distribution and its relationship with chronic disease among residents of a community inW uhan city. Chin J Public Health. 2010; 26:1049–50.
258. Chen MW, Song Y, Yi GQ, Xu SY, Guo B. Diabetes Prevalence and Impaired Fasting Glucose in Urban and Rural Residents of Hubei Province. Acta Med Univ Sci Technol Huazhong. 2010; 39:572–6.
259. Zhang SP, Luo Y. Prevalence and risk factors of diabetes mellitus in Hefeng County. Chin J Prev Contr Chron Dis. 2011; 19:532–3.
260. Wang C. Investigation of precalence and relevant factors of the type 2 diabetes residents in a community of a university in Wuhan. Chinese Journal of Social Medicine. 2012; 29:282–3.
261. Li CF, Zhu CY, Zhang ZF, Duan JJ, Gong J, XIa J, Liu XH, Wan J, Sun HL, Li WP, Li F, Sun HL, Yan YQ, et al. Relationship between blood pressure, lipid and plasma glucose with adult waist line in Wuhan city. J of Pub Health and Prev Med. 2008; 19:28–31.
262. Liu RX, Xie WC, Yang SM, Wang B. Investigation on Diabetes in Residents over 15 Years Old in Caidian District of Wuhan City in 2002. Pre Med Trib. 2007; 13:469–70.
263. Luo XJ, Chen J, Gao SX, Ye CY, Li W. Treatment status of the residents in the Three Gorges reservoir area of diabetes prevalence and medical insurance insured population. China Prac Med. 2012; 7:55–7.
264. Gao XW, Gao XN, Zhang J, Luo CM, Wu YY, Ding ZM, Yang JL, Wang Y. A survey of diabetes and impaired glucose regulation in rural residents of Yiling OF hubei province. China J Diabetes. 2012; 20:913–6.
265. Zhang XF, Hu X. A case-control study of the risk factors of diabetes among the population in coal mine area. Modern Preventive Medicine. 2007; 34:3079–80.
266. Gao Y, Zhou WH, Cao GW, Mu L. Analysis of physical examination results of teaching and administrative staff in Hubei Vocational-Technical College. Journal of Hubei Vocational-Technical College. 2006; 9:99–101.
267. Zhao LS, Chen YS, Xiang GD, Hou J, Le L, Jiang W, Cao HY, Xu L, Zheng M. A survey of prevalences of impaired glucose tolerance and diabetes mellitus in the middle-aged and elderly in areas of Wuhan Distrct. Chin J Diabetes. 2005; 13:448–9.
268. Ma WF, Shen SK, Wang W, Shi P, Zhang Y. An epidemiological study on prevalence of diabetes mellitus and its risk factors in adults of Jianghan Oil Field. J Clin Intern Med. 2004; 21:475–7.
269. Li CX, Xu QH, Wang WL, Zhang YP, Wang JC, Hou XY, Liu SH, Ning WZ, Gou NH, Tian XP, Gao ZG, Wu J. Dynamic observation and comparison of the prevalence of diabetes in Jujube area. hinese Journal of Prevention and Control of Chronic Non-Communicable Diseases. 2002; 10:237.
270. Yu Y. Control the rising trend of diabetes prevalence in our school. Chin J School Doctor. 2002; 16:38.
271. Li J, Gan PZ, Wen C. Prevalence of diabetes in Hubei area. Chinese Journal of Diabetes. 1999; 7:167.

272. Wang ZZ, Huang XZ, Tang SB, Chen YM, Chen LG, Jin ZX, Luan XJ, Zhang J. Relationship between Hypertension, Age and NIDDM, IGT. Chinese Journal of Prevention and Control of Chronic Non-Communicable Diseases. 1997; 5:162–4.

273. Wong MC, Leung MC, Tsang CS, Lo SV, Griffiths SM. The rising tide of diabetes mellitus in a Chinese population: a population-based household survey on 121,895 persons. Int J Public Health. 2013; 58:269–76.

274. Ko GT, Chan JC, Tsang LW, Critchley JA, Cockram CS. Smoking and diabetes in Chinese men. Postgrad Med J. 2001; 77:240–3.

275. Cockram CS, Woo J, Lau E, Chan JC, Chan AY, Lau J, Swaminathan R, Donnan SP. The prevalence of diabetes mellitus and impaired glucose tolerance among Hong Kong Chinese adults of working age. Diabetes Res Clin Pract. 1993; 21:67–73.

276. Lam TH, Liu LJ, Janus ED, Lam KS, Hedley AJ. Fibrinogen, other cardiovascular risk factors and diabetes mellitus in Hong Kong: a community with high prevalence of Type 2 diabetes mellitus and impaired glucose tolerance. Diabet Med. 2000; 17:798–806.

277. Wang G, Liu YJ, Sun CL, Lv Y, Xiao XC, Gao Y, Tian SY, Liu Y, Li YZ, Li M, Cheng J, Gao Y, Sun ZH, et al. Prevalence of diabetes mellitus in Changchun City aged 40 and over in 2011. Chinese Journal of Gerontology. 2014; 34:1072–3.

278. Zhang JB, Zhu YL, DING D, Wang YJ, Zhang D, Liang SJ, Hou Z. Analysis on the prevalent rate of diabetes and its awareness rate among residents from national disease surveillance points in Jilin province. Chin J of Public Health Eng. 2013; 12:332–3.

279. Zhao JM, Yang W. Epidemiological investigation and analysis of adult hypertension in Erdao district of Changchun City. Chin J Public Health Eng. 2013; 12:253–6.

280. Yan Y, Zhang J, Zhang H. Analysis of fasting blood glucose in 27341 patients in the west of Changchun, 2011-2012. Chinese Journal of Clinical Research. 2012; 25:1147.

281. L ZhuY, Guan XL, Ding D, Fu HB, Hou Y, Zhang J. Epidemiological survey of diabetes mellitus in urban and rural communities in Jilin Province. China Tropical Medicine. 2007; 7:806–8.

282. Zhu YL, Li CM, Guan XL, Fu HB, Ding D. Analysis of the risk factor s of chronic diseases among monitored population in Jilin Province. Disease Surveillance. 2007; 22:127–30.

283. Zh YL u, Wang LP, Cai XC, Fu HB, Ding D, Hou Y, Zhang JB, Zhang D, Liu YM, Wang S. Nutrition and Health Status of Residents in Medium Cities and Rural Areas of Jilin Province. Chinese Journal of Public Health Engineering. 2008; 7:12–4.

284. Jiao CW, Zhang JM, Zhang YT, Zheng YF, Liu Y. Analysis of the six levels of diabetes epidemiology in Jilin Province in 1996. Jilin Medical Journal. 1998; 19:264–6.

285. Gu J. Analysis on the status of community diabetes patients. China Health Care Nutrition. 2013: 89.

286. Xu DL, Li Q, Yang T, Zeng S, Tang W. A survey on population's major chronic disease in the community of Nanjing. Acta Universitatis Medicinalis Nanjing. 2014; 34:1392–4.

287. Wang J. Results of diabetes screening of 408 residents aged 40-70 years. Chin J School Doctor. 2014; 28:500, 4.

288. Ge Q, Xia Y. The prevalence of diabetes in residents of Baixia District of Nanjing city in 2012. Jiangsu J Prev Med. 2013; 24:44–5.

289. Yan KG, Chen AZ, Zhou QF, Du Z. Health status of 482 government employees in Pukou District of Nanjing. Chinese Journal of Health Education. 2013; 29:252–4.

290. Feng X. Analysis of health management and chronic diseases in community residents. Chinese Community Doctors. 2012; 14:371–2.

291. Zhu BC, Xie GX, Guo BF, Wang YL, Jiang Y, Feng P. Prevalence of weight related chronic diseases among some community residents in Nanjing City. Occup and Health. 2012; 28:1367–9.

292. Wang J, Tang M, Xiang QY, Gu L. A comprehensive prevention and treatment of chronic diseases in xiaguan district communities of nan jing city. Modern Preventive Medicine. 2009; 36:3560–2.

293. Ni M, Xie Z. Epidemiology And the related factors of diabetes in Nangang workers. Chinese Medicine of Factory and Mine. 2007; 20:486–7.

294. Luo Q, Cong XN, Han L, Tang WQ, Xu B, Chen WH, Liu ZH, Luo D. Prevalence and risk factors of diabetes mellitus of the residents in a community of Nanjing. Prac Geriatr 2010; 24:219–20.

295. Chen YY, Tan YC g, Hong X. The prevalence of type 2 diabetes mellitus and its influence factors in Gaochun County. Jiangsu J Prev Med. 2013; 24:59–61.

296. Li F, Ma Y, Zhang J. Analysis of hypertension and diabetes mellitus in 35 years old people in Xiangcheng District, Suzhou City. J Disease Monitor & Control. 2014; 8:443–4.

297. Ding JS, Song LH, Zhang Q, Peng H, Wang CJ, Fu YL, Zhang Y. Prevalence and Risk Factors of Type 2 Diabetes Mellitus in Residents of a District in Suzhou City J Environ Occup Med. 2014; 31:430–3.

298. Linchi Wang, Hu Y. Investigation on prevalence and risk factors of diabetes in Suzhou. Modern Preventive Medicine. 2012; 39:2145–7.

299. Zhu DD, Tang Y, Pan EC, Hu W, Zhang Q. Analysis of the prevalence and risk factors of diabetes mellitus in Huaiyin District, Huaian City, Jiangsu Province. Chinese Journal of Health Education. 2014; 30:449–51.

300. Qiao CG, Xie Y, Dai GH, Wang ZG, Liu B. Investigation on the prevalence of diabetes in rural residents in Gaoyou city in 2012. Jiangsu J Prev Med. 2014; 25:76–7.

301. Cai B, Lin L, Sun F. Analysis on prevalence and risk factors of hypertension, diabetes, hyperlipidemia of Rugao residents. Jiangsu J Prev Med. 2013; 24:21–2.

302. Zhan YQ g, Su J, Lv SR, Pan XQ, Tao R, Zhang FY, Xiang QY, Wu M. Analysis on diabetes awareness and its influential factors of urban and rural residents in Jiangsu Province. Chinese Journal of Health Education. 2013; 29.

303. Wang DD, Lou PA, Li RG, Zong H, Chen PP, Chen N. Epidemiological characteristics and risk factors of diabetes mellitus aged 15 years and over in Jiawang District of Xuzhou City. China's rural health. 2013: 68-9.

304. J ZhengZ, Dong H, Fang F, Chen Y, Yuan L. A survey on the prevalence of hypertension and health education in Heping District, Xuzhou City. Chin J School Doctor. 2013; 23:723–4.

305. Zhang X, Che L, Gen RJ, Lu M. An Epidemiological Survey on Diabetes Mellitus among Production-line Workers in Xuzhou Coal Mine. Journal of Nursing(China). 2008; 15:15–7.
306. Gu XY, Wang HY, Jiang F. Investigation on chronic disease epidemic status and its relationship with risk factors in Zhenjiang. Chronic Pathemathol J. 2013; 14:208–10.
307. He HS, Liu Q, Kuang Y. The epidemiological investigation and analysis of diabetes mellitus in a community in Yuhang District of Hangzhou city over 50 years. Modern Practical Medicine. 2012; 24:702–4.
308. Zhu XW, Jiang Y, Shen H, Xu X, Zhu B, Cheng HY, Wang YP, Xu L, Jiang YM, Yao Y, Hu ZB, Piao R. Analysis of fasting blood glucose levels and diabetes risk factors in patients aged over 50 years in Wuxi in 2010. Acta Universitatis Medicinalis Nanjing (Natural Science). 2012; 32:505–8.
309. Xu M, Lin YD, Qian Y, Chen F, Bai JL, Yang JY, Gong LF, Zhang T. Cross-sectional investigation of prevalence of type 2 diabetes in urban community residents of Wuxi City. Chinese Modern Medicine. 2010; 17:4–6.
310. Sun N, Zhu Y. Epidemiologic survey on the diabetes mellitus (DM) among the residents in Wuxi. Modern Medicine Journal of China. 2009; 11:50–3.
311. Fan Y. Epidemiological survey analysis of the prevalence of diabetes in the communities of Changzhou. Occup and Health. 2006; 22:1960–1.
312. Zhu HM, Wu JF, Zhou Y. Characteristics of diabetes mellitus in community population in Wujin District of Changzhou City. Jiangsu Health Care. 2008; 10:17–8.
313. Lin JD, Liu J, An X. Investigation on the Prevalent Status and its Influential Factors of Non-communicable Chronic Disease(NCD)among Residents above 35 Years Old in Xuzhou. Pre Med Trib. 2006; 12:13–6.
314. Pan XQ, Yuan BJ, Hang WS, Shi ZM, Dai Y. Study on the characteristics and related factors of diabetes mellitus in urban and rural residents in Jiangsu Province. Jiangsu Prev Med. 2006; 17:6–9.
315. Huang JP, Wu GL, Chen DL, Li G. Investigation and analysis of diabetes mellitus in Nantong City. Med J of Communications. 2006+; 20:469–70.
316. Liu X. The prevalence and risk factors of diabetes in the elderly of Huankou Town, Feng County Chin J School Doctor. 2011; 25:587–9.
317. Zhang YF, Zhou H. Epidemiological Survey of Diabetes among Students' Parents of Taizhou Polytechnic College. Journal of Taizhou Polytechnic College. 2011; 11:86–7.
318. Tang WJ, Lu W. Analysis of blood glucose test results of 1082 community residents in Qidong. Lab Med Clin. 2011; 8:2275–6.
319. Su Y, Wang DY, Shen MZ, Weng W. A survey on Nutrition and Health Status of Residents in Changshu C ity. Occup and Health. 2010; 26:546–9.
320. Ren FL, He Y. Prevalence rate of diabetes mellitus and the awareness rate of diabetes mellitus complications of people in rural communites. Chinese Journal of Clinical Rehabilitation. 2005; 9:26–7.
321. Xia Q, Li L, Tang JL, Zhou C. Epidemiological survey and analysis on prevalence of chronic disease among residents in Haidian County. Jiangsu Health Care. 2001; 3:13–4.
322. Song HF, Xie GQ, Song YY, Gao XZ, Zhu YW, Mu PD, Zhang HY, Xiao BJ, Chen JW, Liu Q. Investigation on the prevalence of diabetes mellitus and its influencing factors in Donghai County. Chinese Primary Health Care. 2001; 15:36–7.
323. Yin JM, Ma JP, Zhang J, Mu W. Investigation on the prevalence of diabetes in rural areas of Suzhou. Suzhou University Journal of Medical Science. 2002; 22:795–6.
324. Wu JN, Yao YH, Lu ML, Qian SF, Tang R. Epidemic features of diabetes in Xishan City, Jiangsu province. Lit & Inf Prev Med. 1999; 5:324–5.
325. Li XS, Xuan YQ, Zhou Y. An explorative study on epidemic features of diabetes in Wujin city. Sh J Prev Med. 1999; 11:307–9.
326. Shen HB, Yu SZ, Xu Y. Risk factors for type 2 diabetes and community prevention. Chinese Primary Health Care. 1999; 13:27–8.
327. Lu MQ, Zhu L, Chen JW, Liu Q. Prevalence and risk factors of diabetes mellitus in 00 cases of coal mine workers in Xuzhou, Jiangsu Province. Journal of Jiangsu Clinical Medicine. 1999; 3:89–91.
328. Hang WS, Pan X. Prevalence, awareness, treatment and control of diabetes mellitus in Jiangsu urban and rural areas. China Prac Med. 2008; 3:141–3.
329. Li JS, Shen J, Liu Y. Prevalence of Diabetes Mellitus in Chinese Among ZhangJing Town , Wuxi County , Jiangsu Province. ACTA Academiae Medicinae Nanjing. 1996; 16:421–4.
330. Tao Y. Survey data analysis of high risk population of diabetes in Nanchang city in 2012. Mod Diag Treat. 2014; 25:1068–9.
331. Wang CX, Huo YN, Wang WQ, Ning G. Investigation on the prevalence of daibetes and analysis on the corresponding treatment and compliance status among elderly in community. Modern Preventive Medicine. 2014; 141:1786–9.
332. Tu P, Shen JS, Xu DB, Duan P, Wu HP, Ding X, Shi L. Prevalence of diabetes mellitus and its risk factors in Xihu Distri t of Nanchang. Shanghai Med J. 2009; 32:720–4.
333. Wu Q, Yang H. Prevalence of diabetes in elderly patients with hypertension in Jiujiang. Practical Journal of Cardiovascular, Cerebrovascular, and Pulmonary Vascular Disease. 2014; 22:56–7.
334. Liu J, Zhu LP, Li A, Luo WQ, Yan W, Ji L, Chen YY, Zhao J, Kong G. Survey on Epidemic Situation and Inf luencing Factors of Diabetes Mellitus Among Urban and Rural Residents in Jiangxi Province. Practical Preventive Medicine. 2011; 18:1637–8.
335. Cai Q LH, Chen T, Zhu MC. Mononucleotide polymorphism at +45 and +276 site of adiponectin gene in aircrew with metabolic syndrome. Clin J Med Officers. 2010; 38:813–5.
336. Zhong GL, Lv WM, Dong MH, CQ Liu, Gao XM, Xu R, Xiao L. A survey of diabetes prevalence in 2908 public officials of ganzhou city. Modern Preventive Medicine. 2008; 35:1338–40.
337. Wang YH, Xu F, Gu N, Wang F, Zhan X. Investigation on the Prevalence of Chronic Diseases among Urban and Rural Residents in Dalian City in 2006. Pre Med Trib. 2010; 16.
338. Zou SF, Shi JP, Gao XH, Li XF, Qiao J, Tang W. Prevalence of hypertension and diabetes complication among people with enterprise medical insurance pension. Chin J Public Health. 2010; 26:284–6.

339. Xu F, Wang YH, Zhan XM, Fan X, Zhen T. Investigation on the Prevalence of Diabetes among Residents over 20 in Dalian City in 2006. Prev Med Trib. 2008; 14:513–4.
340. Han Y. Investigation and analysis of 602 adult residents in Ganjingzi District. Medical Journal of Liaoning. 2008; 22:302–3.
341. Sun Z, Zheng L, Xu C, Zhang X, Li J, Liu S, Hu D, Sun Y. Prevalence of diabetes and impaired fasting glucose in hypertensive adults in rural China. Acta Cardiol. 2009; 64:351–6.
342. Yan Y, Zhang J, Zhu H. Analysis of fasting blood glucose in 27341 patients in the west of Changchun, 2011-2012. Chinese Journal of Clinical Research. 2012; 25:1147.
343. Zhang Y. Investigation on Diet and the Prevalence of Chronic Disease among Rural Middle-aged and OldWomen in Zhuanghe City in 2006. Prev Med Trib. 2009; 15:225–6.
344. Gao ZN, Wang Y, Sun W, Zhang P, Gao XH, Li XF, Zhang CL, Cheng R, Song G. A stratification multi-level cluster sampling survey on relationship between obesity and diabetes mellitus in 2497 citizens from Dalian. Chinese Journal of Clinical Rehabilitation. 2003; 7:2550–1.
345. Dong GH, Liu QN, Teng YZ, Wang SG, Liu ZL, Zhu DQ, Zhang S. Analysis on prevalence and risk factors of chronic disease in residents of Dandong. Chin J Public Health. 2004; 20:467–8.
346. Li SG, Shi YS, Bai S, Luo J, Zhao B. Survey on the awareness, treatment, and control rates as well as the risk factors in patients with diabetes in Bajing community of Shengyang city. Chinese Journal of Clinical Rehabilitation. 2005; 9:6–7.
347. Wang Z, Dong MK, Li DF, Zhao ZZ, Wu D. The prevalence of diabetes in civil aircrew. Chin J Aerospace Med. 1999; 10:243.
348. Liu T. The Analysis of Glucose and Other Metabolic Disorders in Dalian Population. Medicine and Philosophy (clinical decision making Forum). 2006; 27:32–4.
349. Jiang S, She N n, Li H. Investigation on the prevalence of diabetes mellitus in Shenyang aircraft manufacturing company. Chinese Journal of Public Health. 1998; 14:357.
350. Hao L, Su BL, Lin QH, Li CC, Xu ZH, Li P, Wang YN, Zhu P. A survey of the prevalence of diabetes of 3195 people in urban and rural areas of Dalian in 1994. Journal of Dalian Medical University. 1996; 18:229–32.
351. Gao JX, Qin SP, Wang Y. Hohhot diabetes epidemiology investigation and analysis J Disease Monitor & Control. 2014; 8:668–9.
352. Bao MZ, Yu JY, Wang YW, Bai HM, Hao JQ, Hao Y. Analysis on the prevalence of diabetes mellitus and its influencing factors in Xilinhaote City. Chin J Convalescent Med 2014; 23:463–5.
353. Wang RL, Chen G. Epidemiological investigation of chronic non communicable diseases in community residents Huimin District Hohhot City. Inner Mongolia Med J. 2012; 44:1219–20.
354. Su R, Zhuo L, Lang QC, Ding L. Epidemiological study on Hulun Buir City Ewenki diabetes. Journal of Practical Diabetology. 2008; 4:44–5.
355. Wang FQ, Li C. A morbidity rate study on diabetesmellitus in inner mongolia autonomous region. Acta Acad Med Nei Mongol. 1999; 21:71–5.
356. Gao FR, Wu Y, Gao XW, Zhou J. A study on the relationship between living habits and the prevalence of diabetes in the elderly. Inner Mongolia Med J. 2006; 38:1152–4.
357. Mongolia DGoI. Diabetes survey report in the Inner Mongolia Autonomous Region. Inner Mongolia Medical Journal. 1981; 1:45–9.
358. Yao Wang, Bai Y. Analysis of health examination results of 1638 oil workers. Chinese And Foreign Medical Research. 2014; 12:93–4.
359. Fan HY, Ru S, Yang JJ, Wang Z. Epidemic present situation on Community citizens Diabetes in Yinchuan city J Disease Monitor & Control. 2014; 8:611–3.
360. Ma RF, Wang XP, Li J. The prevalence of diabetes in the elderly population in Hui. Journal of Hebei United University (Health Dcience). 2013; 15:623–4.
361. Wen J, Zhang R. The relationship between blood glucose and weight of 1510 cases in Yinchuan City. Ningxia Med J. 2013; 35:843–4.
362. Yang ZG, Liu L, Liu XY, Ma ZM, Li Z. Study on the prevalence of type 2 diabetes and risk factors among residents from partial communities of Y inchuan City and Rural. Ningxia Med J. 2009; 31:399–400.
363. Yin CJ, He JL, Yin XX, Li X. Analysis on the prevalence and risk factors of type 2 diabetes mellitus in 1980 physical examination personnel. Ningxia Med J. 2010; 32:1202–4.
364. Ning YH, Yao L, Li K, Zhang L. Analysis on Diabetes mellitus Prevalence and its Risk Factor of aged people in Yinchuan. Modern Preventive Medicine. 2012; 39:5297–9.
365. Chen X, Yang Y, Xie F, He Y, Wang Q, Li L. Dynamic Monitoring of Chronic Diseases and Risk Factors in a District of Ningxia during 2004 to 2010. Journal of Ningxia Medical University. 2012; 34:357–60.
366. Yang Y, Chen LL, Qiang Y, Liu HR, Wang Y, Song H. Epidemic Situation and Risk Factors of Type 2 Diabetes in Occupational Population of Ningxia. Ind Hlth & Occup Dis. 2011; 37:65–8.
367. Ma H, Li J. The relationship of the different plasma glucose levels and diabetes situation of the adults in Linwu area. Ningxia Med J. 2008; 30:156–7.
368. Li YJ, Wang YK, Bi Y. A survey of health status and main chronic non communicable disease prevention and control in Huangzhong County. Qinghai Journal of Medicine. 2013; 43:64–6.
369. Wei WH, Zhou MR, Sha QY, Zhou SX, Xu ZH, Zhang S, Guo S. Prevalence and risk factors of diabetes mellitus in Qinghai Province. J Med Pest Control. 2013; 29:62–6.
370. Zhang HL, Gao JD, Dai QX, Ma FM, Li J, Wu Z. An Investigation of Diabetes Prevalence in Qinghai Province. Journal of High Altitude Medicine. 2009; 19:11–4.
371. Cui J. Analysis on the prevalence of diabetes mellitus in Taining Garden Community. Qinghai Journal of Medicine. 2003; 33:49–50.
372. Wang YZ, Gao JD, Su WY, Wang RY, Liu XY, Li H. Investigation report of diabetes prevalence in Xining Province. Journal of Qinghai Medicine. 1982: 13-5.
373. Wang YZ, Su WY, Wang RY, Gao JD, Li HL, Liu XY, Xu WN, Zhao YZ, Guo BC, Bao YS, Zhang HM, Gao F. Diabetes survey report in two areas of Qinghai Province (Huangyuan and Gonghe). Journal of Qinghai Medicine. 1981: 1-11.

374. Qiao Y, Gu Y. Hyperlipidemia among the Middle-elderly People in Community. Health Education and Health Promotion. 2011: 152-8.
375. Zhang Y. Analysis of prevalence and risk factors of diabetes mellitus of Dachang Area Combined Committee. China Medical Engineering. 2014; 22:180–1.
376. Gu YP, Wu L. Survey analysis of 308 cases of elderly patients with chronic diseases and health education Modern health. 2014: 276.
377. Villegas R, Xiang YB, Elasy T, Cai Q, Xu W, Li H, Fazio S, Linton MF, Raiford D, Zheng W, Shu XO. Liver enzymes, type 2 diabetes, and metabolic syndrome in middle-aged, urban Chinese men. Metab Syndr Relat Disord. 2011; 9:305–11.
378. Xia ZC, Zhu DZ, Wang L, Liu XY, Zhao S. Investigation and risk factors of type 2 diabetes mellitus in community in Yanji, Shanghai. Contemporary Medicine. 2012; 18:152–4.
379. Xiang F, Shao YQ, Zhang YY, Lu YQ, Zhu L. Analysis of the diabetes epidemic characteristics and influencing factors of residents above 35 years old in Shanghai Jiading district. Chinese Journal of Social Medicine. 2012; 29:367–9.
380. Tian L, Tang J. The Relationship between Body Mass Index and Waist line and t he Prevalence of Diabetes Mellitus in Community Residents. China modern Doctor. 2010; 48:89–90.
381. Xu XF, Zhu XG, Yin YL, Gu ML, Chen DH, Jin ZM, Zhang W. Yexie town residents sugar adjust damaged and type 2 diabetes illness status investigation. Modern Preventive Medicine. 2012; 39:1940–3.
382. Xu J, Liu J, Cha Y, Wang F, Ding HY, Zhao Y. The Prevalence of Elderly Diabetic in Shanghai Minhang Community. Chinese Journal of Clinical Medicine. 2011; 18:198–200.
383. Tang JH, Wang ZP, Lin H. Investigation on the prevalence of elderly diabetes mellitus in a suburb of Shanghai City. Journal of Community Medicine. 2011; 9:46–8.
384. Cao B, Shen L, Liu F, Xu JP, Ma H. Survey on Health State of Residents in Shanghai Suburbs and Related Problems. Chinese Journal of General Practice. 2011; 9:942–4.
385. Zhang YF, Huang Y, Chen XO, Guan YY, Liu K, Su YX, Bi YF, Ying X. Prevalence of diabetes and impaired glucose regulation and risk factors in suburb area of Shanghai. Chinese Journal of Practical Internal Medicine. 2011; 31:74–5.
386. Li R, Lu W, Jiang QW, Li YY, Zhao GM, Shi L, Yang QD, Ruan Y, Jiang J, Zhang SN, Xu WH, Zhong WJ. Increasing prevalence of type 2 diabetes in Chinese adults in Shanghai. Diabetes Care. 2012; 35:1028–30.
387. Chen J. Investigation on the relationship between overweight / obesity and chronic diseases in elderly people in a community in Shanghai. Journal of Chinese Community Doctors. 2011; 13:72–3.
388. Xu RF, Peng LX, Huang YF, Bai H. Prevalence of diabetes among residents in a town of Qingpu District, Shanghai. J Environ Occup Med. 2011; 28:361–3.
389. Jiang GL, Wang YF, Guan JF, Qi XL, Zhou P, Che M, Zhang C, Zhang J. Survey of diabetes mellitus and its risk factors among permanent elderly inhabitants in the countryside of nanxiang area in shanghai. Modern Preventive Medicine. 2008; 35:2620–1.
390. Zhu M. Study on behavioral risk factors of chronic disease in Yueyang community, Songjiang district. J Environ Occup Med. 2008; 25:282–5.
391. Wang LF, Wang Y, Han J, Liu B. Epidemic Status and Analysis on the Risk Factor s of Diabetes Mellitus. Chinese Primary Health Care. 2008; 22:30–1.
392. Tayama J, Li J, Munakata M. Working Long Hours is Associated with Higher Prevalence of Diabetes in Urban Male Chinese Workers: The Rosai Karoshi Study. Stress Health. 2014.
393. Xing HL, Jin J, Qin L, Feng YW, Hu JF, Chen XR, Chen HB, Fang WJ, Dong Y. Investigation of prevalence and related risk factors of diabetes mellitus in Baoshan district of Shanghai. Journal of Shanghai Jiaotong University (Medical Science). 2007; 27:1268–70.
394. Chen J. Prevalence, awareness rate and risk factors of diabetes mellitus in elderly people in the community of down town. Journal of Chinese Community Doctors. 2010; 12:247.
395. Chunxiang Wu, Jianhong Ma, Yang X. Survey on the nutrition and health status of residents in putuo district of shanghai in 2007. Modern Preventive Medicine. 2009; 36:1442–4.
396. Li X, Feng B, Ni YF, Huang YW, Le XK, Fu M, Chen MH, Zhao ZF, Zhou FY, Meng ZY, Wang HL, Yao GL, Zhang R, et al. Influence of differnent diagonstic criteria of diabetes on distribution and patten of carbohydrate metabolism among inhabitants above 40 in Shanghai Pudong New District. Shanghai Med J. 2006; 29:283–5.
397. Li R, Lu W, Jia WP, Li YY, Shi L, Liu MX, Geng GZ, Fu H, Shi R, Shi JH, Shi HL, Zhang S. Cross-sectional investigation of prevalence of type 2 diabetes in Shanghai. Natl Med J China. 2006; 86:1675–80.
398. Huang XY, Feng B, Li X, Ni YF, Zhang R. A Survey of Diabetes Mllitus and Comparison Analysis of Related -risk Factors Among Permanent Inhabitant in Lujiazui Community and Jinyang Community of Shanghai City. Chinese Primary Health Care. 2011; 25:13–4.
399. Zhang XW, Li M, Peng WF, Yan J. Epidemiologic Survey of Diabetes Mellitus in Urban Adults. Journal of Tongji University (Medical Science). 2004; 25:425–7.
400. Mi M, Song J, Chen M, Liu H, Zou SR, Shi AZ, Jiang PZ, Gao WW, Cheng M. Investigation on prevalence of diabetes mellitus among some Shanghai residents. Sh J Prev Med. 2005; 17:208–12.
401. Yang J, Zhou LF, Guo LY, Gao E. Study on prevalence and knowledge of diabetes mellitus in middle-aged and elderly population in a community. Chin J Public Health. 2005; 21:131–3.
402. Xu ZX, Xu LQ, Lu L. Study on the prevalence of diabetes mellitus and its prevention and cure of 1000 population in the Bund City, Shanghai City. Chinese Primary Health Care. 1999; 13:26–7.
403. Sheng ZY, Liu M, Wang YF, Zhang HD, Wang JB, Ge YX, Wang YP, Huang YH, Peng LM, Wang LJ, Zhang AF, Shao AH, Hu Y. A survey of diabetes prevalence in 9376 urbanite adults of Shanghai. Chinese Journal of Diabetes. 2001; 9:214–7.
404. Shi YX, Gu HX, Li Y, Wang MS, Huang Y. Investigation and analysis of the prevalence of diabetes mellitus and

complications of retired elderly retired cadres. Chin J Naut Med. 1999; 6:186–8.
405. Bai XL, Zhang X, Du H. Analysis of the Prevalence of Diabetes Mellitus in Beixinjing Community in ChangNing District. Health Education and Health Promotion. 2006; 1.
406. Zhao MY, Sun HY, Lei LJ, Jin TX, Liu H. Prevalence and risk factors of elderly diabetes in community. Shanghai Journal of Preventive Medicine. 2008; 20:295–8.
407. Jiang ZC, Zou YW, Zhang YG, Li L. Survey on the epidemic situation and risk factors of diabetes mellitus among residents, Rushan City. Prev Med Trib. 2014; 20:820–4.
408. Wang Y, Huang S, Tang JL, Jiang ZC, Zou YW, Ma J. Prevalence of chronic disease and influence factors among middle-aged and senior adults aged over 50 in rural areas of Rushan city, 2009. Prev Med Trib. 2012; 18:252–4.
409. Yang L. Epidemiological study of type 2 diabetes in middle aged and old people. For All Health. 2014; 8:118–9.
410. Liu WL, Li ZH, Zhong CM, Xu GX, Liu JD, Dou W. Epidemiological survey on diabetes mellitus among residents in a community, Shengli Oil Field. Pre Med Trib. 2014; 20:481–3.
411. Lin YJ, Yang XY, Yu ZZ, Ma L, Gao X. Analysis of physical examination results of 30351 elderly people in Ji'nan City. Shandong Medical Journal. 2014; 54:85–6.
412. Xin Q, Zhang CQ, Jin CX, Yang CZ, Wu J. Prevalence of diabetes and its influencing factors in senior college staffs. Chinese Health Service Management. 2013: 556-9.
413. Lu Y, Liang T. Investigation on the prevalence of diabetes and hypertension in military health. Chin J Convalescent Med. 2010; 19:956–7.
414. He P. Survey of prevalence of diabetes mellitus and its relevancy with nutrition intake among rural residents in Junnan County. Pre Med Trib. 2012; 18:261–5.
415. Tao MY, Yu BM, Wan J. Investigation on the Prevalence of "Hyperlipemia , High Blood Pressure and Hyperglycemia"among the Employees of Ji'nan Railway Station from 2006 to 2007. Prev Med Trib. 2009; 15:1005–7.
416. Zheng XL, Xu Y. Survey on the Prevalence of Hypertension, Diabetes and Hyperlipemia among Railway Workers over 45 in Ji'nan. Prev Med Trib. 2009; 15:416–7.
417. Li SP, Li YM, Chen CH, Liu T. A cross-sectional study of impaired fasting glucose and diabetes in Ji'nan railway workers. Chinese Journal of Health Statistics. 2011; 28:420–3.
418. Xu J, Shen WW, Li Y. Analysis on the prevalence of disease in different age groups in 355 retirees. J Prev Med Chin PLA. 2005; 23:358–9.
419. Li JX, Zhou X. Survey on the prevalence of 467 elderly residents in Zhoucun District of Zibo City. Journal of Community Medicine. 2013; 11:60–2.
420. Xue C. Investigation and analysis of diabetes mellitus and impaired glucose regulation in the workers and their families of Qilu Petrochemical Industries Co. Medical Journal of Chinese People's Health. 2006; 18:550,8.
421. Dong Y, Gao W, Nan H, Yu H, Li F, Duan W, Wang Y, Sun B, Qian R, Tuomilehto J, Qiao Q. Prevalence of Type 2 diabetes in urban and rural Chinese populations in Qingdao, China. Diabet Med. 2005; 22:1427–33.
422. Liu JJ, Sun HB, Zhong Q, Liu A. The analysis of abnormal glucose metabolism of Qingdao residents in 2010. Modern Preventive Medicine. 2013; 40:1071–80.
423. Ning F, Pang ZC, Dong YH, Gao WG, Nan HR, Wang SJ, Zhang L, Ren J, Tuomilehto J, Hammar N, Malmberg K, Andersson SW, Qiao Q. Risk factors associated with the dramatic increase in the prevalence of diabetes in the adult Chinese population in Qingdao, China. Diabet Med. 2009; 26:855–63.
424. Zhou X, Pang Z, Gao W, Wang S, Zhang L, Ning F, Xue B, Chen X, Qiao Q. Fresh vegetable intake and prevalence of diabetes in a Chinese population in Qingdao. Diabetes Res Clin Pract. 2011; 92:137–42.
425. Zhai YM, Feng ZC, Wang SJ, Zhang Y, Ren J, Zhang K. Investigation on the Prevalence of Chronic Disease in Qingdao in 2002. Prev Med Trib. 2007; 13:97–9.
426. Wang Y. Analysis of prevalence and risk factors of type 2 diabetes in Chengyang District of Qingdao City. Shandong Medical Journal. 2010; 50:58–60.
427. Li Y, Lin SX, Li XD, Wang YX, Yin F. Investigation on the Prevalence of Diabetes among Rural Population over 35 in Laoshan District of Qingdao in 2006. Prev Med Trib. 2009; 15:28–9.
428. Qingdao DGo. Prevalence of type 2 diabetes in Qingdao. Chin J Diabetes. 2004; 12:344–7.
429. Tian JB, Yue L. Investigation on the prevalence of chronic diseases in elderly cadres in Qingdao City. Lit & Inf Prev Med. 1999; 5:24,79.
430. Zhu C. Prevalence and economic burden of hypertension and diabetes mellitus in community residents. China Health Care & Nutrition. 2013: 1496-7.
431. Liu Z, Jiang Y, Meng Y. Analysis of the incidence rate of diabetes mellitus in teachers. China Health Care & Nutrition. 2013: 398.
432. Liu C. Investigation of chronic non communicable diseases and related risk factors in Ningyang County. Chinese Rural Health Service Administration. 2012; 32:1136–8.
433. Chen X, Guo XL, Ma JX, Tang JL, Lu ZL, Zhang JY, Zhang GH, Xu A. Analysis of anthropometric cut points for screening diabetes mellitus among residents aged 18-69 in Shandong Province. Journal of Shandong University (Health Science). 2012; 50:19–23.
434. Tang X, Tang H. Analysis on the screening of high risk population in Yiyuan County in 2011. Medical Innovation of China. 2012; 9:87–8.
435. Xue AQ, Liu S, Wang W. Investigation and Countermeasure of chronic diseases of workers aged 45 years and over. Chinese Journal of Ethnomedicine and Ethnopharmacy. 2010: 57.
436. Zhong CM, Liu JD, Li HP, Xu GX, Li Z. A Cross Sectional Study On The Prevalence Of Diabetes among Residents In Binnan Community Of Shengli Oil Field,2009-2010. Prev Med Trib. 2011; 17:135–6.
437. Wei YT, Zhang CJ, Gu N, Wang L. Investigation and Analysis on the prevalence of diabetes and impaired fasting blood glucose in physical examination in Yantai. Chin J Convalescent Med. 2011; 20:849–51.

438. Ha QS o, Wang ZY, Deng L. Study on the Correlation of Diabetes Prevalence in Zaozhuang City Residents. Hebei Medicine. 2007; 13:384–7.
439. Liu TJ, Zhang Z, Gao Z. Current status of chronic diseases in Changshan county and analysis on their impact factors. Chin J of PHM. 2009; 25:636–7.
440. Chen JM, Jia C. A Cross-sectional Study on Non-communicable Disease in Lijin County in 2007. Prev Med Trib. 2008; 14:596–8.
441. Zhang Z. Characteristics of diabetes mellitus in community residents in Shengli Oilfield. Journal of Community Medicine. 2008; 6:24–5.
442. Li H, Ma JX, Xia H, Tu SY, Meng Q. Investigation on knowledge of hypertension and diabetes mellitus and related lifestyle in residents of rural Shandong. Chin J Public Health. 2008; 24:1458–60.
443. Yu HB, Pan XJ, Li Z. Survey on the prevalence of diabetes in rural areas of Pingdu city. China modern Doctor. 2007; 45:44.
444. Jiang XY, Yang Y, Liu XC, Shi XZ, Zhao YH, Xie CR, Yang XM, Huang XH, Sun K, Hui RT, Zhang H. Investigation on current status of hypertension and diabetes mellitus in middle-aged population-The health study in Qingdao Harbore. Molecular Cardiology of China. 2007; 7:281–3.
445. Tang Y. Analysis of the prevalence of diabetes mellitus and age in Liaocheng area. Chinese Journal of Ethnomedicine and Ethnopharmacy. 2010: 95.
446. Zhu AX, Wang YT, Li Z. Investigation on Chronic Diseases and Consultation among Zaozhuang Urban and Rural Residents. Prev Med Trib. 2006; 12:8–10.
447. Sun S. The investigation and analysis of prevalence of diabetes mellitus among city residents in dezhou city. Modern Preventive Medicine. 2011; 38:3933–4.
448. Yan J, Zhang SL, Li M, Wang JF, Zhao C. Investigation on the prevalence of diabetes mellitus and complications of retired cadres in Ji'nan. Chinese Journal of Diabetes. 1995; 3:188–9.
449. Zhang FH, Lin H. Analysis on Incidence and Risk Factors of Diabetes Among Adults in Ji'nan. Practical Preventive Medicine. 2008; 15:237–9.
450. Guo YL, Duan ZH, Zhou LN, Quan H. The prevalence of chronic diseases of urban and rural residents in Xinjiang in 2013. Prev Med Trib. 2014; 20:456–8.
451. Li WD, Song C. Investigation and analysis of hypertension and diabetes on residents over the age of 18 in Shouyang County, Shanxi Province. Chinese Journal of Health Education. 2014; 30:471–3.
452. Shen XM, Song X. Investigation and Analysis on Fasting Blood Glucose, Blood Lipids and Serum Uric Acid Levels in Community Groups and Regular Physical Examination Groups of Taiyuan. Chin J Arterioscler. 2011; 19:697–701.
453. Tang YP, Li ML, He JH, Zuo CG, Gao YZ, Huang AL, Yan H. Investigation on prevalence of abnormal glucose metabolism and risk factors of community group in Taiyuan city. Chinese Nursing Research. 2009; 23:1326–8.
454. Ma WP, Li HX, Gong WJ, Zong QQ, Gao LN, Chen L. An epidemiological investigation and analysis of diabetes mellitus aged 40 years and over in Changzhi area. Journal of Practical Diabetology. 2011; 7:59,61.
455. Xing XW, Guo JJ, Hou YY, Wang WP, Liu X, Wei FM, An J. Epidemiological survey and analysis of diabetes among inhabitants in Yuncheng area of Shanxi Province. Chin J Public Health. 2009; 25:3–4.
456. Zhao SF, Hou YY, Rao HX, Zhao MX, Guo JJ, Liu X, Zhang YH, Zhao JY, Yang R. Cross-sectional Study on D iabetesM ellitus among U rban and R ura l Residents in Shanxi Province. Chin Prev Med. 2009; 10:5–9.
457. Yang J, He GF, Zhang JC, Wang C. Investigation of diabetes mellitus, 25 years old and over 11734 in Shanxi Province. Shanxi Medical Journal. 1998; 27:123–5.
458. Li PW, Xu XM, Gao GZ, Zhao R, He JH, Guo ZX, Chen H. Epidemiological survey of diabetes in three units of Taiyuan City, Shanxi Province. Journal of Shanxi Medical College. 1995; 26:223–5.
459. Shānxi DGo. Investigation report on the prevalence of diabetes mellitus in thirty thousand farmers in Shanxi Province. Journal of Shanxi Medical College. 1981; 2.
460. Lei HM, Wang X. Situation of hypertension and diabetes prevalence/control in Beiguan area, Xi'an City Contemporary Medicine. 2010; 16:22–4.
461. Liu CM, Yuan ZM, Luo C, Dong W, Wang YX, Qiao W. Investigation on the prevalence of diabetes in elderly people in Xianyang city of Shhănxi. Modern Traditional Chinese Medicine. 2006; 26:77–8.
462. Feng YL, Meng RY, Fan Z. Investigation and Analysis on the prevalence of diabetes mellitus in Jinzhong City. Shanxi Medical Journal. 2007; 36:803–4.
463. Yang CL, You SL, Yang PR, Wang H, Lan ZC, Tan W. Investigation on prevalence of diabetes mellitus and hypertension in residents of Mei County. Practical Preventive Medicine. 2011; 18:1993–4.
464. Liu YL, Gong MY, Wang HM, Hu MY, Hu HH, Feng B. Epidemiological study on diabetes in workers of Xi'an railway area. Modern Medical Journal. 2002; 30:151–3.
465. Jia XL, Chen S, Zhou XF, Zhang XJ, Sun XC, Chen P, Fang XM, Liu Y, Zhang M. Investigation of the prevalence and characteristics of newly diagnosed diabetes and IGR in the population of disaster areas of Wenchuan earthquake Chin J Diabetes. 2011; 19:892–5.
466. Li H, Liu Y, Fang XM, Liao D, Ye WC, Yang BL, Liu XY, Jiao Y, Qi XL, Hu N. Prevalence of Diabetes Mellitus in Beichuan County after Earthquake. Today Nurse. 2010: 70-1.
467. Cao C. Guanghan City residents population over 18 years, hypertension, diabetes prevalence research. China Health Industry. 2014: 41-5.
468. Zhang HT, Gao L, Liu Y, Pang Y, Zhou Q, Long G. An epidemiological study on diabetes mellitus and cerebral stroke in Ganzi TIbetan State. Sichuan Medical Journal. 2014; 35:645–7.
469. Yang KC, Xu FL, Xu J, Liu B, Qing Y, Su X, Wang H, Fu T. Epidemiological survey and analysis of diabetes mellitus in Taifu County, Luzhou City. Biotech World. 2013: 80.
470. Li XZ, Bai X, Zeng J, Liu F, Wan Q. A city in elderly diabetes, prediabetes prevalence and related factors. Guide of China Medicine. 2012; 10:412–4.
471. Wei L, Li Z. Prevalence and Risk Factors of Type 2 Diabetes Mellitus in Wuhou District of Chengdu. Journal of Occupational Health and Damage. 2012; 27:330–4.

472. Xu XH, Peng HS, Xiao XR, He XY, Xu TY, Liang GJ, Zhong JY, Zhao T, Chen J. Health examination results of military male middle-aged and senile cadres from Chengdu city in 2006 and 2010. Medical Journal of National Defending Forces in Southwest China. 2012; 22:527–9.
473. Li LX, He S, Chen XP, Wan lY, Yang R, Chen JJ, Liu K, Lv Z. Effects of Body Mass Index and Abdominal Obesity on Prevalence of Diabetes Mellitus and Fasting Blood-glucose in Elderly People in Chengdu. West China Medical Journal. 2012; 27:504–7.
474. Wang RL, Xu JB, Zhang TJ, Wu YL, Wang J, Ruan L, Zhang L, Wang WY, Zeng J. The Current Study on Impaired Glucose Metaboli sm and Its Relation to Cardiovascular Risk Factors in Chengdu City. Chin J Prev Contr Chron Non-commun Dis. 2003; 11:56–8.
475. Hu R, Zhang Y. Prevalence of diabetes mellitus in community residents in Shuangliu County, Sichuan Province. J Prev Med Inf. 2006; 22:92–3.
476. Luo XC, Huang XB, Liu Y, Ouyang LY, Liu JX, Hu YM, Yi YJ, Huang B. Prevalent study of diabetes mellitus among elderly people in Chengdu area. Sichuan Medical Journal. 2011; 33:1505–8.
477. Ye XR, Wang PJ, Sun HP, Wang WH, Zhang Z. Analysis of prevalence rate and risk factors of diabetes mellitus in 1677 elderly veterans. Chinese Journal of Clinical Rehabilitation. 2004; 8:4942–3.
478. Zou B. Zizhong Country 600 Residents of Glucose Test Results. China Health Industry. 2012: 5,7.
479. He HJ, Wei DY, Wang CX, Zhang JH, Wang B, Ma MJ, Pan L, Shan G. Type 2 diabetes in cit izens of X ichang c ity, Sichuan province: a cross-sectional study. Chin J Public Health. 2010; 26:1252–4.
480. Pu Y, Zhang M. Adult prevalence rate of diabetes mellitus in Renhe District of Panzhihua City. Journal of North Pharmacy. 2011; 8:94–5.
481. Chen XF, He J, Deng Y, Zhang NM, Ji K, Wang Z. Prevalence o fMajor Chronic Non-communicable Disease sand Control Measures Taken by Patients in Surveillance Areas in Sichuan, 2004. J Prev Med Inf. 2009; 25:820–3.
482. Zhao YZ, Feng SF, Li GM, Liu H, Xie XS, He MH, Liu D. Risk factors of type 2 diabetes in Nanchong community. Med J West China. 2011; 23:2024–7.
483. Lan XY, Li X. Investigation report on the prevalence of diabetes in Luzhou City. Journal of Luzhou Medical College. 2001; 24:239–40.
484. Liu YX, Liu LP, Feng AP, Liu DG, Tang AQ, Liu XL, Tian GF, Lv QX, Li D, Luo Q, Qu H. Study on prevalence of diabetes and its distribution in population of sichuan luzhou chemicalgroup corporation. Modern Preventive Medicine. 2002; 29:476–8.
485. Qin F, Zhan TJ g, Ruan L, Zhu S, Zhang J, Chen M, Fan LM, Zhou LC, Zhang L, Wang W. Diabetes mellitus combined with hypertension and its influence on the analysis of 7288 cases in Chengdu. Chinese Journal of Hypertension. 2002; 10:91–3.
486. Zhang Y, Wang D. An investigation on the prevalence of diabetes millitus among 336 health workers. Chin J Epidemiol. 1999; 20:98–101.
487. He TM, Chen S, Zhang XJ, Lv YC, Li PQ, Li M, Chen P, Wang H. Prevalence of diabetes mellitus in Jiange County, Sichuan Province. Journal of Sichuan Continuing Education College of MS. 1997; 16:235,40.
488. Shen FF, Shu ZZ, Liu X. A survey of diabetes mellitus in 2457 patients over 40 years of age. Med JNDFSC. 1994: 80.
489. Li QF, Li ZF, Zhang H, Zou XH, Liang Y. Prevalence of type 2 diabetes mellitus in Kunming and Chengdu area. Journal of PLA. 2004; 6:218.
490. Sun F, Tao QM, Tao QS, Yang XH, Cao CJ, Zhan S. Type 2 diabetes prevalence and estimated risks on developing type 2 diabetes within 5 years for adults aged 35-74 based on Chinese Taiwan MJ health-checkup database. Chin J Dis Control Prev. 2013; 17:369–73.
491. Chiu TH, Huang HY, Chiu YF, Pan WH, Kao HY, Chiu JP, Lin MN, Lin CL. Taiwanese vegetarians and omnivores: dietary composition, prevalence of diabetes and IFG. PLoS One. 2014; 9:e88547.
492. Chang C, Lu F, Yang YC, Wu JS, Wu TJ, Chen MS, Chuang LM, Tai TY. Epidemiologic study of type 2 diabetes in Taiwan. Diabetes Res Clin Pract. 2000; 50 Suppl 2: S49-59.
493. Chen HD, Shaw CK, Tseng WP, Chen HI, Lee ML. Prevalence of diabetes mellitus and impaired glucose tolerance in Aborigines and Chinese in eastern Taiwan. Diabetes Res Clin Pract. 1997; 38:199–205.
494. Chen KT, Chen CJ, Gregg EW, Engelgau MM, Narayan KM. Prevalence of type 2 diabetes mellitus in Taiwan: ethnic variation and risk factors. Diabetes Res Clin Pract. 2001; 51:59–66.
495. Liu XQ, Song CH, Li CP, Cui Z, Wei FJ, Ke H, Zhu B, Liu YY, Ma J. Epidemiological survey of chronic diseases in community residents in Tianjin City. Chinese Journal of Health Statistics. 2012; 29:667–9.
496. Wang HY, Geng XL, Zhu H, Wang JH, Liu X. Epidemiological Investigation of Type 2 Diabetes in Old Age Population in Tianjin. Tianjin Med J. 2011; 39:112–4.
497. Chang G, Tian Y, Wang DZ, LI W, Jiang G. Analysis on Current Status and Influential Factors of Diabetes among Residents from Different Districts in Tianjin. Chin J Prev Contr Chron Dis. 2011; 19:144–7.
498. Liu HM, Hu B, L BW i, Wang YJ, Ni M, Zhang Y. The Current Status of Diabetes Mellitus Complication and its Awareness in Residents of Tanggu District, Tianjin. Chin J Prev Contr Chron Dis. 2009; 17:372–6.
499. Qian D, Wang Y, Wang GD, Qi X. Prevalence rate of type 2 diabetes in community residents and its related factors: a cross-sectional study. Chin J Public Health. 2009; 25:1441–2.
500. Tian H, Song G, Xie H, Zhang H, Tuomilehto J, Hu G. Prevalence of diabetes and impaired fasting glucose among 769,792 rural Chinese adults. Diabetes Res Clin Pract. 2009; 84:273–8.
501. Geng X, Wang XX, Liu XM, Song GD, Zhu H, Wang FS, Wang J. A Study on Prevalence of Type 2 Diabetes in Tianjin. Tianjin Med J. 2009; 37:101–3.
502. Li W, Jiang GH, Wu TY, Wang Z. The prevalence of diabetes mellitus and its effect factors among rural and urban residents of Tianjin. Occup and Health. 2009; 25:828–30.

503. Yang Y, Pan Y, Li J, Jiang GH, Chang G, Zhang F. Investigation on blood glucose status of rural community residents in tianjin. Modern Preventive Medicine. 2007; 34:2319–21.
504. Zhang X. Prevalence survey of diabetes mellitus in Hebei District, Tianjin. Chin J Contr Chron Non-commun Dis. 2001; 9:185–6.
505. Yang WX, Song G. An Epidemiological Survey and Analysis on Prevalence of Chronic Disease among Residents in Tianjin. Chinese Journal of Public Health. 1999; 15:979–80.
506. Wang ZJ, Ren TS, Wang MS, Chen SH, Wang Z. The Study on Preval ence Rate of Diabetes Mell itus of the Adul t in Urban Areas of Tianjin. Chin J Prev Contr Chron Non- commun Dis. 1999; 7:80–2.
507. Zhang Y, Shen CH, Huang JH, Xie HX, Song G. Health Checkup Results of Rural Residents Aged 15 yrs and over in Tianjin in 2004. Chin J Prev Contr Chron Non-commun Dis. 2006; 14:386–8.
508. Liu Y. Analysis of factors associated with high incidence of diabetes mellitus. Contemporary Medicine. 2010; 16:55–6.
509. Rexidanmu. Wusiman. Effect of dyslipidemia on the morbidity of diabetes and its related risk factors. China Modern Medicine. 2012; 19:188,90.
510. Adi DR, Wang HY, Ma YT, Yang YN, Liu F, Xie X, Gao YL, Cao Y. Epidemiology investigation of prevalence of type 2 diabetes among community residents in Karamay Refinery. Journal of Xinjiang Medical University. 2014; 37:1550–3.
511. Chen LS, Gan H, Wu J. Analysis on Survey on the Life Quality among the Aged in Dushanzi District of Kelamayi City, 2007. Prev Med Trib. 2010; 16:990–5.
512. Wu SL, Zhou C, Yilihamu, Yi FS, Zhang MT, Zhu YQ, Huang L. A study on prevalence of diabetes and metabolic syndrome in Xinjiang Uyghur and Han nationalities. J Intern Med Concepts Pract. 2012; 7:198–200.
513. He YX, Yumusi A., Fu J, Li LZ, Lu Q. Study on the blood pressure, blood glucose, obesity in elderly residents of Karamay City Baijiantan district over the age of 60. Contemporary Medicine. 2010; 16:149–50.
514. Liu Z, Dilimulati, Wang MH, Liu J. Investigation on the prevalence of chronic kidney disease in patients with hypertension and diabetes mellitus in rural areas. China Health Industry. 2014: 145-6.
515. Yang HM, Jian Q g, Daoken Y., Xiong LL, Li M. Analysis of community diagnosis of chronic non communicable diseases in Kuitun city in 2013. Bull Dis Control Prev. 2014; 29:52–3.
516. Yin TP, Li JB u, Qin JM, Chen MX, Guo J. Survey analysis on the main chronic diseases of Xinjiang production and Construction Corps residents,1998 - 2010. Bull Dis Control Prev. 2014; 29:7–9.
517. Li YQ, Zhang RM, Zhao W, Liao J. Survey of prevalence and risk factors of type 2 diabetes mellitus of Uyghur rural residents in seed multiplication farm Aksu city. Journal of Xinjiang Medical University. 2013; 36:1375–8.
518. Jiang T, Zhang LR, Li D. Analysis of physical examination results of people over 50 years old Guangming Road Community in Shawan County. Bull Dis Control Prev. 2013; 28:53–4.
519. Luo WP, LX Liu, Ma JP, Suo FY, Guan YM, Liao PH, Zhe W. Survey and analysis of behavior risk factors related to chronic diseases among residents at surveillance sites of Xinjiang in 2010. Bull Dis Control Prev. 2013; 28:20–3.
520. Sun GF, Xie HF, Zhao E, Song F, Aji Y, Guo GL, Wan L. Survey on diabetes prevalence and knowledge awareness in Urumqi City. Chinese Journal of Health Education. 2013; 29:42–4.
521. Tian XQ, Zhao LM, Zhao FC, Sun N. Investigation on the prevalence of diabetes mellitus in 3293 adult residents in Xinjiang. Xinjiang Medical Journal. 2010; 40:10–4.
522. Abulizi A., Wang L, Ma YT, Yang YN, Huang D, Li XM, Ma X, Liu F. Study on the Prevalence of Impaired Fasting Glucose and Diabetes Mellitus in Uygur Population from Xinjiang. Chinese Journal of General Practice. 2011; 9:1275–7.
523. Zhou P, Duosenbaike Y., Zhang Y, Wang XH, Zhu DL, Chen N. Prevalence of abnormal glucose metabolism in population of different ethnics in Ili Prefecture. Chin J Endocrinol Metab. 2006; 22:351–3.
524. Tao Y, Mao X, Xie Z, Ran X, Liu X, Wang Y, Luo X, Hu M, Gen W, Zhang M, Wang T, Ren J, Wufuer H, et al. The prevalence of type 2 diabetes and hypertension in Uygur and Kazak populations. Cardiovasc Toxicol. 2008; 8:155–9.
525. Sun JG, Lu Y, Qian R, Zhang J, Teng HJ, Li J. Epidemiological survey of diabetes prevalence in the Tarim Petroleum Exploration Area. Xinjiang Medicial Journal. 2007; 37.
526. Yang YN, Xie X, Ma YT, Li XM, Fu ZY, Ma X, Huang D, Chen BD, Liu F, Huang Y, Liu C, Zheng YY, Baituola G, et al. Type 2 diabetes in Xinjiang Uygur autonomous region, China. PLoS One. 2012; 7:e35270.
527. Huang Y. A study on the diabetes epidemiology of community residents in the seven division of Xinjiang Corps. Journal of Bingtuan Medicine. 2009; 20:57–9.
528. Su LQ, Wang FP, Wang X. Analysis on the related factors of community diabetes in the new urban area of Urumqi, Xinjiang. Xinjiang Medicial Journal. 2009; 39:12–3.
529. Mayinuer, Yilamu, Cai M. Preliminary analysis on the risk factors of diabetes mellitus in Xinjiang armed police. Xinjiang Medicial Journal. 2005; 35:1–3.
530. Yi X, Su LQ, Wang ZH, Liu JB, Yang Y. Analysis of risk factors on type 2 diabetes mellitus in some communities of Urumchi. Chinese Journal of Clinical Rehabilitation. 2005; 9:24–5.
531. Xu ZX, Zhang LZ, Xiripu M., Aishan M., Tuohuti K., Cheng Q, Dong YL, Yimiti, Aishanjiang. The prevalence of diabetes in Xinjiang Uygur Moyu County. Chinese Journal of Primary Medicine and Pharmacy. 2002; 9:221.
532. Wu JR, Nie C, Yang ZF, Zhu QL, Tong G. Analysis on the characteristics of elderly diabetes in Urumqi. Lanhou Health. 1992; 13:23–4.
533. Wu SL, Ma J, Li N, Gao H. An Analysis of Risk Factor s for Diabetes in Kar amay, Xinjiang. Chin J Prev Contr Chron Non-commun Dis. 2008; 16:186–8.
534. De J, Ci Y, Da P, Amina. Type 2 diabetes mellitus and impaired fasting blood glucose among Tibet citizens. Chin J Public Health. 2008; 24:1467–8.

535. Shen XG, Wang Z. Analysis of the incidence of hypertension, diabetes and hyperlipidemia in different occupational groups. Chinese Journal of Convalescent Medicine. 2012: 206-8.

536. He R, Cai L, Dong J, Tao J, Zhang S. Analysis of the prevalence and economic burden of diabetes in Guandu District of Kunming. Journal of Kunming Medical University. 2014; 35:12–4.

537. Peng BK, Gao XZ, Gao H, Kong FB, Lin B, Wang X. Prevalence of diabetes mellitus in Kunming, Yunnan, 45 years of age and older people. Journal of Practical Diabetology. 2013; 9:38–42.

538. Yan F, Zhang MR, Yang Z, Shen L, Li ZK, Tian R. Analysis of prevalence and awareness rate of hypertension and diabetes in Kunming City. Chin J Prev Contr Chron Dis. 2012; 20:331–2.

539. Gong HQ, Zhang MR, Tian R. Physical activity and the occurrence rate of type 2 diabetes. Chin Prev Med. 2010; 11:1229–32.

540. Cao G. Analysis of health examination results of retired people. China Medical Herald. 2011; 8:138–9.

541. Zhang MR , Shen L, Li ZK, Yang Z, Yang JQ, Ma Y, Liu Y, Gong H. A cross-sectional study on diabetes mellitus in rural population in Kunming. Chin J Prev Contr Chron Non-commun Dis. 2008; 16:614–6.

542. Yang Z, Zhang MR, Li YT, Yan ZM, Gong H. Study on epidemiology of type 2 diabetes in Kunming. Soft Science of Health. 2004; 18:337–9.

543. Shu ZK, Cai L, Ye YH, Bi WH, Li HD, Feng R, Huang W. Prevelence of diabetes and influencing factors in rural area of Shilin county. Chin J Public Health. 2009; 25:1529–30.

544. Yang LP, Zhao J. Epidemiological survey of 1519 diabetic patients in Ninger County Soft Science of Health. 2013; 27:236–8.

545. Cao AH, Wang KL, Xia ZX, Li RJ, Zhou R, Zhang Z. A screening survey of diabetes mellitus in 5000 peasants of Bai nationality in Dali Autonomous region of Yunnan. Chin J Prev Contr Chron Non-commun Dis. 2005; 13:161–2.

546. Yin ZW, Luo P, Zhang JW, Yang L, Wang XQ, Li L. Investigation report of diet and prevalence rate of diabetes among 446 persons aged 40~49 years. Chinese Journal of Diabetes. 1999; 7:82–4.

547. Kunming DGo. Diabetes survey report of 1500 people in Kunming area. Journal of Yunnan Medicine 1990; 11:173–7.

548. Fang J. Investigation and Analysis on the prevalence of diabetes mellitus in aged over 65 years old in a community. For All Health. 2014; 8:31–2.

549. X HM u, Zhu KJ, Zhang RH, Huang LC, He QF, Meng J, Zhang HX, Che ZY n, Ji XQ, Wang L. Nutrition and health status of residents in Wucheng district of Jinhua. Chinese Rural Health Service Administration. 2010; 30:176–8.

550. Zou WJ, Lai SL, Zhou L. Baseline survey of patients with diabetes in community. J Med Theor & Prac. 2014; 27:1250–1.

551. Ye QY, Xiang XQ, Ni XM, Qiu JH, Xu W. An investigation on prevalence and risk factors of type 2 diabetes mellitus among residents of Qingtian County. Zhejiang Preventie Medicine. 2013; 25:5–7.

552. Xu J, Luo YN, Ye Z, Yu M, Zhan XW g, Wang H, Zhang J. Prevalence investigation and risk factors analysis on type 2 diabetes mellitus in adults in Cixi. Zhejiang Preventie Medicine. 2013; 25:8–10.

553. Ji LM, Zhang CH, Chai JS, Gan CS, Qiu LX, Wu JG, Miao Z. Prevalence and r elated factors of diabetes among middle and old age population in Cixi area. Jiangxi Medical Journal. 2007; 42:402–4.

554. Wang F. Prevalence and health knowledge demand of community chronic disease Empty Nester. Journal of Traditional Chinese Medicine Management. 2013; 21:352–3.

555. Hu CR, Zou H, Huang LC, Jiang X. Prevalence of diabetes and its related risk factors among residents in Jianggan district of Hangzhou city. Chin Prev Med. 2013; 14:53–5.

556. Zhen W g, Wu YQ, Lu XJ, Fei J, Zhang Z. Epidemiological investigation and evaluation of military elderly population with diabetes in Hangzhou. Clin J Med Offic. 2012; 40:127–9.

557. Zhou H, Ding L, Sun JM, Xiong KH, Xu X. Analysis of 13615 cases of retired workers in Gongshu District, Hangzhou. China modern Doctor. 2011; 49:49–50.

558. Lu HC, Lin HB, Ding K, Li X. Investigation on prevalence and risk factors of type 2 diabetes mellitus in Yinzhou District. Zhejiang Preventie Medicine. 2013; 25:31–2.

559. Jiang X. Epidemiological analysis of chronic disease Wang town of Ningbo city in 2011 survey. Modern Practical Medicine. 2012; 24:1153–5.

560. Zhu Q. Analysis of the characteristics of Pine Town community women with diabetes. Clinical Rational Drug Use. 2013; 6:36.

561. Pan XJ, Chen GC, Zheng CJ, Yang Y. Analysis of blood glucose level and risk factors in middle aged and old people in Quzhou City. Chinese Rural Health Service Administration. 2013; 33:98–9.

562. Xu XD, Jiang G. Analysis of the current status of diabetes mellitus and its influencing factors in Nanxun District, Huzhou City. Strait J Prev Med 2012; 18:35–6.

563. Fan HH, Qu YY, Liu D, Zhu A. Analysis on public officer of impaired fasting glucose and diabetes and its risk factors in Lishui City, Zhejiang Province. Chinese Journal of Health Education. 2011; 27:943–5.

564. Xu CH, Chen L, Zhu A. Analysis on the Relationship between Body Mass Index and Metabolic Diseases in Elder People. Zhejiang Preventie Medicine. 2010; 22:9–11.

565. Shen YF, Shen Y. An investigation on the prevalence of fasting blood glucose and diabetes mellitus in Wuxing District. Zhejiang Preventie Medicine. 2012; 24:36–7.

566. Ye HJ, Zhang WQ, Zhang RR, Yi AZ, Zhou H. Epidemiologic survey on the diabetes mellitus(DM) among the community residents in Wenzhou. Chinese Rural Health Service Administration. 2012; 32:395–7.

567. Chen RY, Hu YW, Hu D. Diabetes prevalence and risk factors of diabetes in Yongjia County. Modern Practical Medicine. 2009; 21:476–8.

568. Jin S. Analysis of health examination results of 6706 cases of farmers in the suburbs of Shaoxing City. Chinese Rural Health Service Administration. 2010; 30:879–80.

569. Jin HJ, Hu SL, Lei Y, Jiang X. The prevalence survey on the distribution and the relative risk factors of diabetes in some city of Zhejiang province. Chinese Journal of General Practice. 2011; 9:425–6.

570. Feng W, Cao Y. Analysis of health examination results of 6706 cases of farmers in the suburbs of Shaoxing City. Zhejiang Preventie Medicine. 2011; 23:26–8.

571. Ye Z, Pei GJ, Chen Z. Prevalence and related factors of diabetes mellitus in Xiangshan County, Zhejiang Province. Shanghai Journal of Preventive Medicine. 2011; 23:622–3.

572. Zheng JY, Xie JM, Chen P, Shao S. Analysis of the prevalence of diabetes mellitus and its influencing factors in Ouhai District of Wenzhou City. Shanghai Journal of Preventive Medicine. 2011; 23:626–8.

573. Wang L, Teng Y, Qiu X. Risk factors of type 2 diabetes mellitus in the working intellectuals. Zhejiang clinical medicine. 2006; 8:450–1.

574. Zhang LN, Zhang T, Cui J, Chen J. Study on the Prevalence and Risk Factors of Type 2 Diabetes Mellitus in Ningbo. J Environ Occup Med. 2006; 23:50–1.

575. Xu F, Wang YB, Wan L g, Huang Y. Analysis of the prevalence and risk factors of type 2 diabetes mellitus in Ling'an City. Prevention and Treatment of Cardio- Cerebral- Vascular Disease. 2009; 9:139–40.

576. Huang BD, Li Y. Analysis of the situation of 533 policemen in chronic disease. Strait J Prev Med. 2004; 10:40–1.

577. Pan YP, Zheng QD, Chen W, Luo J. Epidemiological survey of diabetes mellitus in rural areas over 35 years old. Prevention and Treatment of Cardio- Cerebral- Vascular Disease. 2012; 12:318–20.

578. Xu WM, Shi SF, Tian JS, Wu LM, Jiang HR, Ye JJ, Jin HY, Zh XX u, Shen Y, Fang S. Study on the Relationship between BMI, WHR and the Prevalence Rate of Diabetes Mellitus in Hangzhou Residents. Chin Prev Med. 2004; 5:337–9.

579. Zhang YF, Wang JL, Chen QF, Chen E. A study on the prevalence of diabetes risk factors and type 2 diabetes mellitus in Shaoxing City. Chin Prev Med. 2004; 5:387–9.

580. Hu RY, Han XJ, Zhong JM, Yu M. Study on The Morbidity of Type II Diabetes and Risk Factors among Residents from Two Communities of Zhejiang Province. Disease Surveillance. 2005; 20:151–3.

581. Xu J, Liu J, Mao Z. A rural-urban investigation and analysis on residents' physique in Quzhou. Chinese General Practice. 2002; 5:302–3.

582. Xu GZ, Wang RY, Xie YL, Xiang ZH, Yang WN, Xiao G. Study on Risk Factors of Adult Diabetes Mellitus in Ningbo City. Journal of Disease Surveillance. 1999; 14:405–7.

583. Mei YM, Hu X. Analysis of diabetes epidemiology in Yuyao City. Chinese Journal of public health. 1999; 18:223–4.

584. Fan CH, Yu M, Chen YP, Zhao M, He Q. Prevalence and Risk Factors of Type Diabetes Mellitus in Residents of Zhejiang Province. Zhejiang Prev Med. 2008; 20:3–5.

585. Zhejiang DGo. Report on Diabetes Epidemiological Investigation in Zhejiang Province from 1994 to 1995. Bulletin of Science and Technology. 1998; 14:44–9.

586. Zhejiang DGo. Diabetes survey report of 31597 people in Zhejiang Province. Journal of Zhejiang Medical University. 1983; 12:185–8.

587. Zhao JY, Zhou L, Lin W, Bian P. Investigation and analysis of the physical examination results of 3302 aged cadres with cardiovascular diseases. Prevention and Treatment of Cardio-Cerebral- Vascular Disease. 2008; 8:119–20.

588. Xu Y, Wang L, He J, Bi Y, Li M, Wang T, Jiang Y, Dai M, Lu J, Xu M, Li Y, Hu N, Li J, et al. Prevalence and control of diabetes in Chinese adults. JAMA. 2013; 310:948–59.

589. He Y, Zhai F, Ma G, Feskens EJ, Zhang J, Fu P, Van't Veer P, Yang X. Abdominal obesity and the prevalence of diabetes and intermediate hyperglycaemia in Chinese adults. Public Health Nutr. 2009; 12:1078–84.

590. Liu S, Wang W, Zhang J, He Y, Yao C, Zeng Z, Piao J, Howard BV, Fabsitz RR, Best L, Yang X, Lee ET. Prevalence of diabetes and impaired fasting glucose in Chinese adults, China National Nutrition and Health Survey, 2002. Prev Chronic Dis. 2011; 8:A13.

591. Liu W, Hua L, Liu WF, Song HL, Dai XW, Yang JK. The prevalence of glucose metabolism disturbances in Chinese Muslims and possible risk factors: a study from northwest China. Arq Bras Endocrinol Metabol. 2014; 58:715–23.

592. Bragg F, Li L, Smith M, Guo Y, Chen Y, Millwood I, Bian Z, Walters R, Chen J, Yang L, Collins R, Peto R, Lu Y, et al. Associations of blood glucose and prevalent diabetes with risk of cardiovascular disease in 500 000 adult Chinese: the China Kadoorie Biobank. Diabet Med. 2014; 31:540–51.

593. Liu J, Zhao D, Qi Y, Sun J, Wang W. Prevalence of diabetes mellitus in outpatients with essential hypertension in China: a cross-sectional study. BMJ Open. 2013; 3:e003798.

594. Hu D, Sun L, Fu P, Xie J, Lu J, Zhou J, Yu D, Whelton PK, He J, Gu D. Prevalence and risk factors for type 2 diabetes mellitus in the Chinese adult population: the InterASIA Study. Diabetes Res Clin Pract. 2009; 84:288–95.

595. Pan XR, Yang WY, Li GW, Liu J. Prevalence of diabetes and its risk factors in China, 1994. National Diabetes Prevention and Control Cooperative Group. Diabetes Care. 1997; 20:1664–9.

596. Wu YF, Xie GQ, Li Y, Zhao LC, Zhou B. The current status on the prevalence, awareness, treatment and control of diabetes mellitus in several Chinese subpopulations. Chin J Epidemiol. 2005; 26:564–8.

597. Zhang LQ, Liu LS, Li W, Jin S. Sampling survey on hypertension and diabetes in the outpatient visitors. Chin J Gen Pract. 2002; 1:16–8.

598. National DRCGo. A survey of diabetes prevalence in the middle-aged and elderly Chinese from 12 areas of China. Chin J Endocrinol Metab. 2002; 18:280–4.

599. Yang W. A study of multi center prospective treatment on the prevalence of diabetes mellitus and the number of patients with impaired glucose tolerance in China in 1994. Bull Med Res. 2002; 31:26–7.

600. Shen HB, Yu SZ, Xu Y. A stuy on morbidity variance and economic burden of diabetes mellitus in China. Journal of Shanghai Preventive Medicine. 1998; 10:387–90.

601. National DRCGo. Prevalence of diabetes and its risk factors in China 1994. Chin J Intern Med. 1997; 36:384–9.

602. Xie NZ, Chen YQ, Chen LB, Wu XZ, He M, Lei T, Zhang X. Survey on the prevalence of diabetes mellitus in 4081 cases. J Shanghai Tiedao Univ (Med Sci). 1996; 10:207–8.

603. Xiang HD, Wang H, Sun Q, Hao JH, Wang HW, Liu YG, Chen T, Feng Y, Liu RW, Xu JP, Han SM, Chi Z. A survey

of diabetes and impaired glucose tolerance in Shanxi, Beijing and Liaoning, north China 1989. Chinese Journal of Diabetes. 1993; 1:16–20.

604. Hu D, Fu P, Xie J, Chen CS, Yu D, Whelton PK, He J, Gu D. Increasing prevalence and low awareness, treatment and control of diabetes mellitus among Chinese adults: the InterASIA study. Diabetes Res Clin Pract. 2008; 81:250–7.

605. Nagahama K, Inoue T, Kohagura K, Ishihara A, Kinjo K, Ohya Y. Hyperuricemia predicts future metabolic syndrome: a 4-year follow-up study of a large screened cohort in Okinawa, Japan. Hypertens Res. 2014; 37:232–8.

606. Nakano T, Ito H. Epidemiology of diabetes mellitus in old age in Japan. Diabetes Res Clin Pract. 2007; 77 Suppl 1: S76-81.

607. Umemura S, Goto Y, Otaka T, Yamaguchi H, Nakano S, Yoshikawa K. An epidemiological study on diabetes mellitus in the Iwaki area. Tohoku J Exp Med. 1983; 141 Suppl: 655-60.

608. Sekikawa A, Eguchi H, Tominaga M, Igarashi K, Abe T, Manaka H, Sasaki H, Fukuyama H, Kato T, Kiyohara Y, Fujishima M. Prevalence of type 2 diabetes mellitus and impaired glucose tolerance in a rural area of Japan. The Funagata diabetes study. J Diabetes Complications. 2000; 14:78–83.

609. Imamura Y, Uto H, Hiramine Y, Hosoyamada K, Ijuin S, Yoshifuku S, Miyahara H, Maenohara S, Oketani M, Ido A, Tsubouchi H. Increasing prevalence of diabetes mellitus in association with fatty liver in a Japanese population. J Gastroenterol. 2014; 49:1406–13.

610. Uehara A, Kurotani K, Kochi T, Kuwahara K, Eguchi M, Imai T, Nishihara A, Tomita K, Yamamoto M, Kuroda R, Nagata T, Omoto D, Murakami T, et al. Prevalence of diabetes and pre-diabetes among workers: Japan Epidemiology Collaboration on Occupational Health Study. Diabetes Res Clin Pract. 2014; 106:118–27.

611. Ohmura T, Ueda K, Kiyohara Y, Kato I, Iwamoto H, Nakayama K, Nomiyama K, Ohmori S, Yoshitake T, Shinkawu A, et al. Prevalence of type 2 (non-insulin-dependent) diabetes mellitus and impaired glucose tolerance in the Japanese general population: the Hisayama Study. Diabetologia. 1993; 36:1198–203.

612. Takahashi Y, Noda M, Tsugane S, Kuzuya T, Ito C, Kadowaki T. Prevalence of diabetes estimated by plasma glucose criteria combined with standardized measurement of HbA1c among health checkup participants on Miyako Island, Japan. Diabetes Care. 2000; 23:1092–6.

613. Kashima S, Inoue K, Matsumoto M, Akimoto K. Prevalence and characteristics of non-obese diabetes in Japanese men and women: the Yuport Medical Checkup Center Study Yuport. J Diabetes. 2015; 7:523–30.

614. Mukai N, Doi Y, Ninomiya T, Hirakawa Y, Nagata M, Yoshida D, Hata J, Fukuhara M, Nakamura U, Kitazono T, Kiyohara Y. Trends in the prevalence of type 2 diabetes and prediabetes in community-dwelling Japanese subjects: The Hisayama Study. J Diabetes Investig. 2014; 5:162–9.

615. Kim SG, Yang SW, Jang AS, Seo JP, Han SW, Yeom CH, Kim YC, Oh SH, Kim JS, Nam HS, Chung DJ, Chung MY. Prevalence of diabetes mellitus in the elderly of Namwon County, South Korea. Korean J Intern Med. 2002; 17:180–90.

616. Kim SM, Lee JS, Lee J, Na JK, Han JH, Yoon DK, Baik SH, Choi DS, Choi KM. Prevalence of diabetes and impaired fasting glucose in Korea: Korean National Health and Nutrition Survey 2001. Diabetes Care. 2006; 29:226–31.

617. Lee HY, Won JC, Kang YJ, Yoon SH, Choi EO, Bae JY, Sung MH, Kim HR, Yang JH, Oh J, Lee YM, Park NH, Ko KS, et al. Type 2 diabetes in urban and rural districts in Korea: factors associated with prevalence difference. J Korean Med Sci. 2010; 25:1777–83.

618. Koo BK, Kim SW, Yi KH, Park KS, Moon MK. Changing relative contribution of abdominal obesity and a family history of diabetes on prevalence of diabetes mellitus in Korean men and women aged 30-49 years from 2001 to 2010 2001201030-49. J Diabetes. 2015; 7:465–72.

619. Choi YJ, Kim HC, Kim HM, Park SW, Kim J, Kim DJ. Prevalence and management of diabetes in Korean adults: Korea National Health and Nutrition Examination Surveys 1998-2005. Diabetes Care. 2009; 32:2016–20.

620. Lawson P. Homelessness--a home for Tom. Nurs Times. 1991; 87:26–9.

621. Park Ie B, Kim J, Kim DJ, Chung CH, Oh JY, Park SW, Lee J, Choi KM, Min KW, Park JH, Son HS, Ahn CW, Kim H, et al. Diabetes epidemics in Korea: reappraise nationwide survey of diabetes "diabetes in Korea 2007". Diabetes Metab J. 2013; 37:233–9.

622. Lee DS, Kim YJ, Han HR. Sex differences in the association between socio-economic status and type 2 diabetes: data from the 2005 Korean National Health and Nutritional Examination Survey (KNHANES). Public Health. 2013; 127:554–60.

623. Chung HR, Perez-Escamilla R. Risk factors of type 2 diabetes among Korean adults: The 2001 Korean national health and nutrition examination survey. Nutr Res Pract. 2009; 3:286–94.

624. Lee HS, Lee SS, Hwang IY, Park YJ, Yoon SH, Han K, Son JW, Ko SH, Park YG, Yim HW, Lee WC, Park YM. Prevalence, awareness, treatment and control of hypertension in adults with diagnosed diabetes: the Fourth Korea National Health and Nutrition Examination Survey (KNHANES IV). J Hum Hypertens. 2013; 27:381–7.

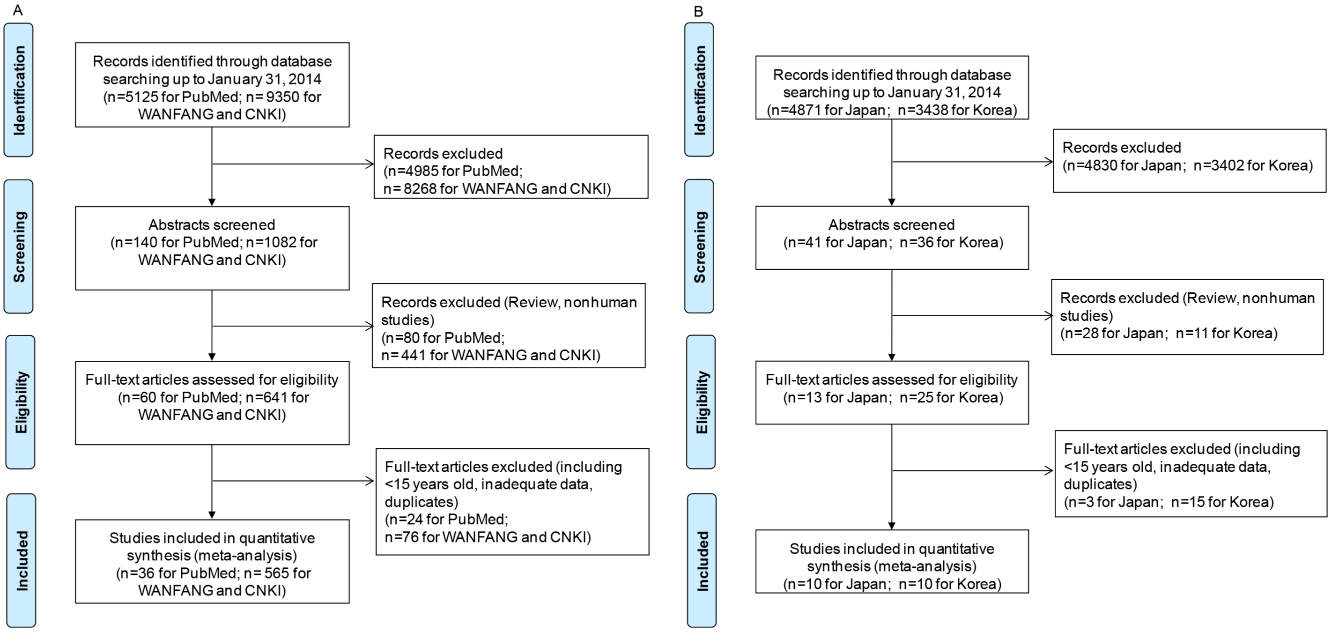

Supplementary Figure 1: Flowchart of study identification. This searching was performed to identify articles up to December 31, 2014 for China (**A**), and Japan and Korea (**B**), respectively.

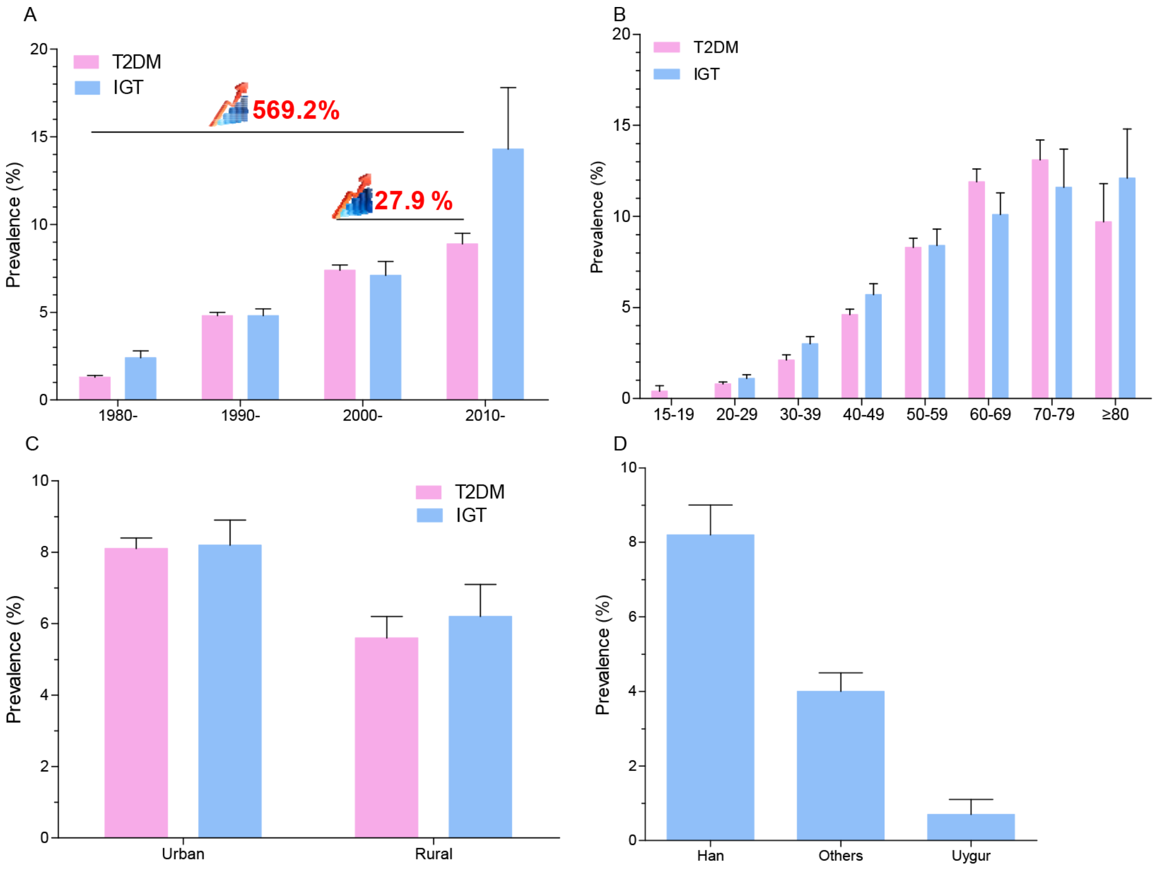

Supplementary Figure 2: Prevalence (%) of T2DM and IGT in the past 35 years by year, age, region and ethnic group. (**A**) T2DM and IGT prevalence increased by year. (**B**) T2DM and IGT prevalence increased with age. (**C**) Prevalence of T2DM and IGT was higher in urban areas than in rural areas. (**D**) T2DM prevalence by ethnic group in China.

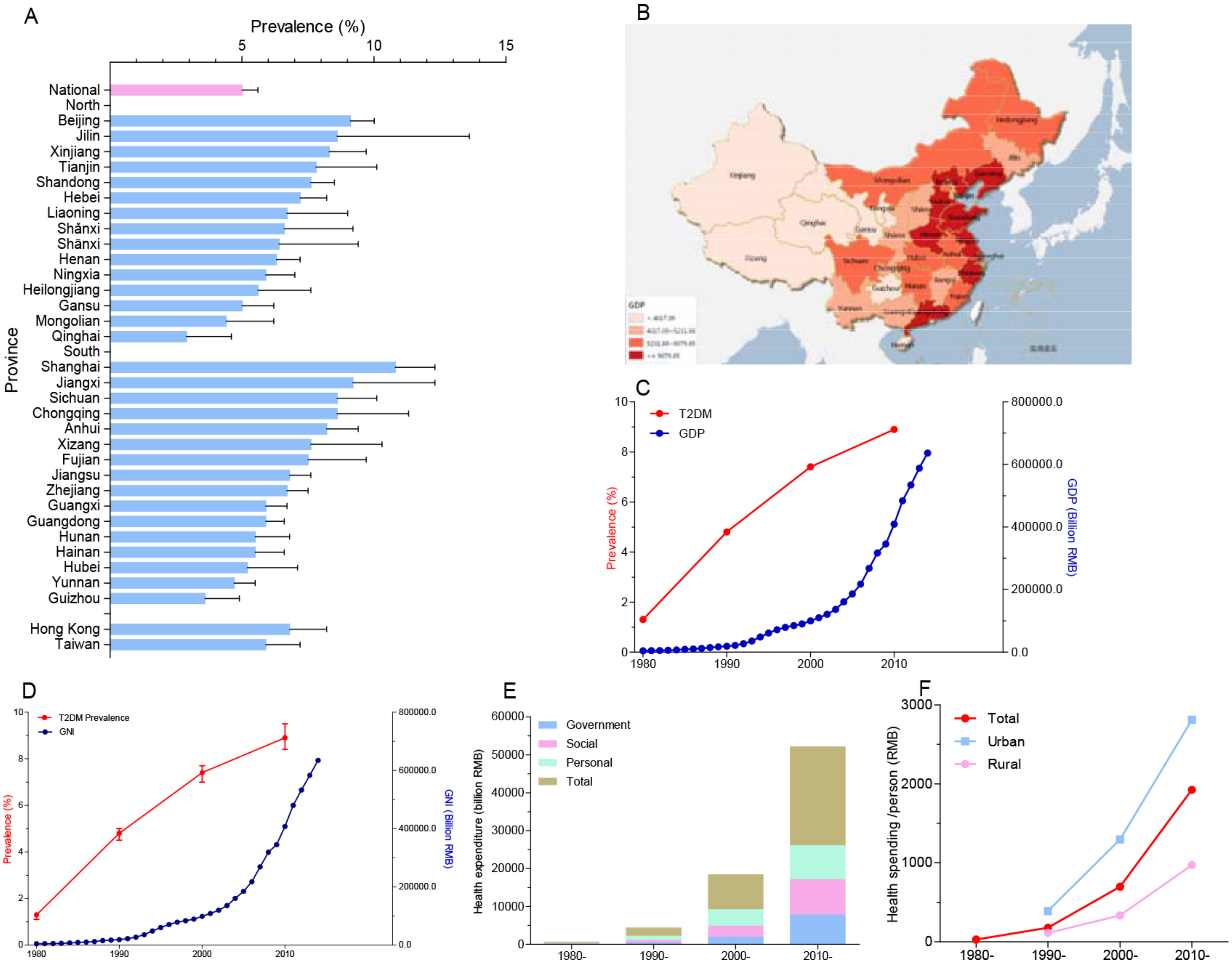

Supplementary Figure 3: Characteristics and burden of T2DM in China. (**A**) The prevalence of T2DM by city in northern and southern mainland China, Hong Kong, and Taiwan; (**B**) Geographic characteristics of GDP in mainland China (Map of GDP was generated using Dituhui (version 2.0; Dituhui Technology Co., Ltd, Chengdu, China; http://www.dituhui.com)); (**C**) Increased T2DM prevalence with increasing GDP from 1980 to 2014; (**D**) Increased T2DM prevalence with increasing GNI from 1980 to 2014; (**E**) Health expenditures from government, society, and individual sources increased by year; (**F**) Health expenditures in urban areas and rural areas

Variable	Coefficient	Std. Error	t-Statistic	Prob.
C	0.002580	0.000154	16.79803	0.0000
AR(4)	-0.362124	0.205633	-1.761024	0.0896
MA(1)	-0.999921	0.060279	-16.58832	0.0000

R-squared	0.522115	Mean dependent var	0.002733
Adjusted R-squared	0.486716	S.D. dependent var	0.020768
S.E. of regression	0.014879	Akaike info criterion	-5.483102
Sum squared resid	0.005977	Schwarz criterion	-5.342982
Log likelihood	85.24653	Hannan-Quinn criter.	-5.438277
F-statistic	14.74945	Durbin-Watson stat	2.082112
Prob(F-statistic)	0.000047		

Inverted AR Roots	.55-.55i	.55-.55i	-.55+.55i	-.55+.55i
Inverted MA Roots	1.00			

Autocorrelation	Partial Correlation		AC	PAC	Q-Stat	Prob
		1	-0.544	-0.544	10.974	0.001
		2	0.210	-0.122	12.661	0.002
		3	-0.129	-0.097	13.320	0.004
		4	-0.138	-0.340	14.100	0.007
		5	0.155	-0.142	15.120	0.010
		6	-0.229	-0.319	17.410	0.008
		7	0.202	-0.243	19.263	0.007
		8	0.072	0.072	19.507	0.012
		9	-0.127	-0.095	20.300	0.016
		10	0.074	-0.189	20.577	0.024
		11	-0.092	-0.095	21.023	0.033
		12	0.103	0.035	21.617	0.042
		13	-0.056	0.018	21.801	0.059
		14	-0.068	-0.104	22.086	0.077
		15	0.198	0.115	24.605	0.056
		16	-0.244	-0.108	28.666	0.026

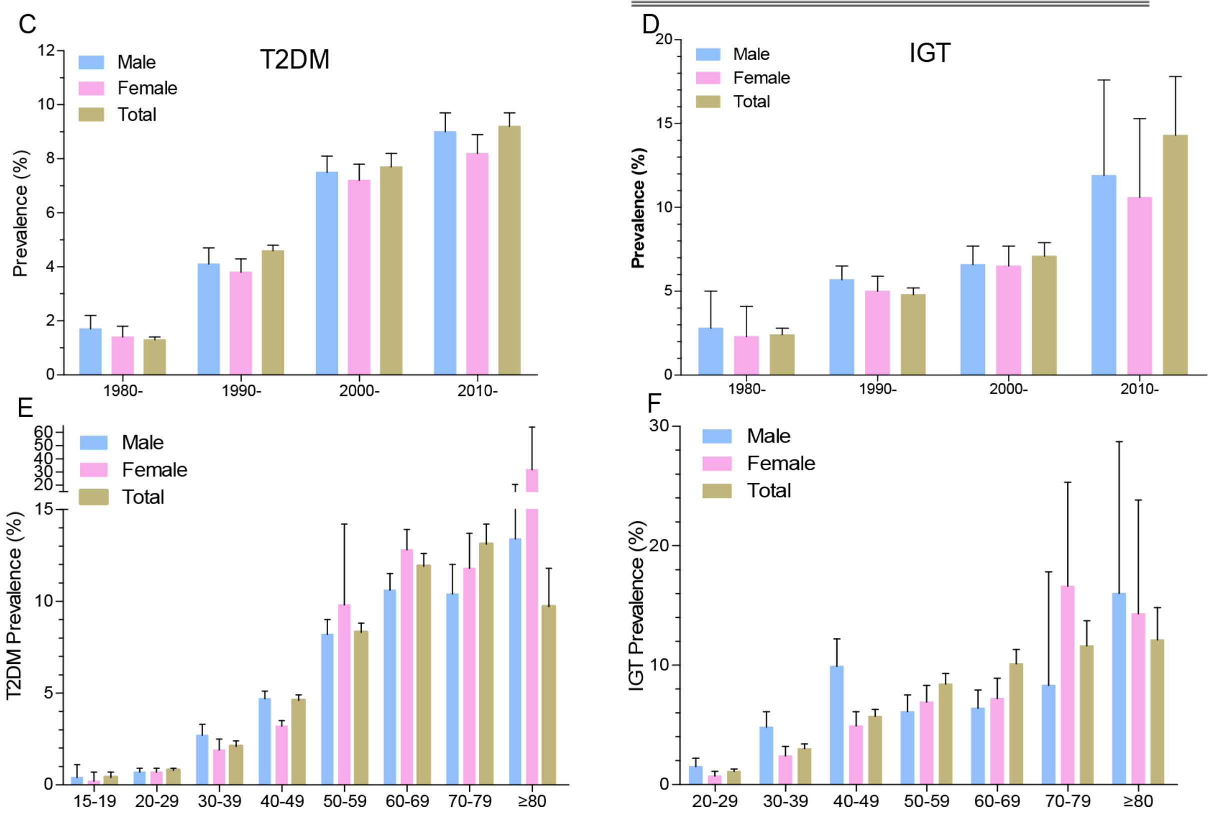

Supplementary Figure 4: Time series analysis for T2DM prevalence and T2DM/IGT prevalence stratified by year, age and gender. (**A**–**B**) Time series analyses of T2DM over the past 35 years and for the next 10 years; (**C**–**D**) T2DM and IGT prevalence stratified by year and gender; (**E**–**F**) T2DM and IGT prevalence stratified by age and gender.

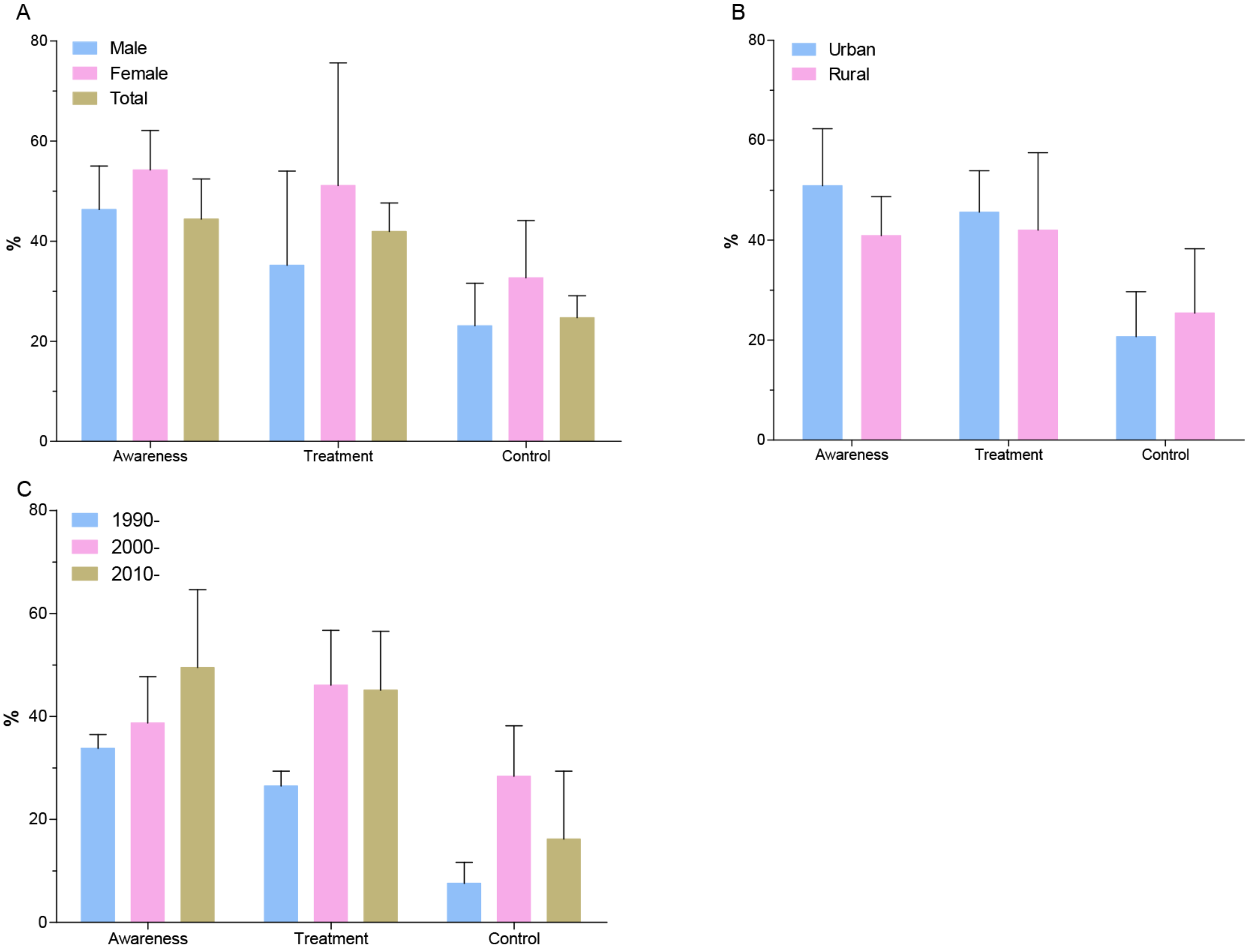

Supplementary Figure 5: Awareness, treatment, and control of T2DM in China. (**A**) The rates of awareness, treatment, and control of T2DM stratified by sex; (**B**) The rates of awareness, treatment, and control of T2DM stratified by region; (**C**) The rates of awareness, treatment, and control of T2DM stratified by year.

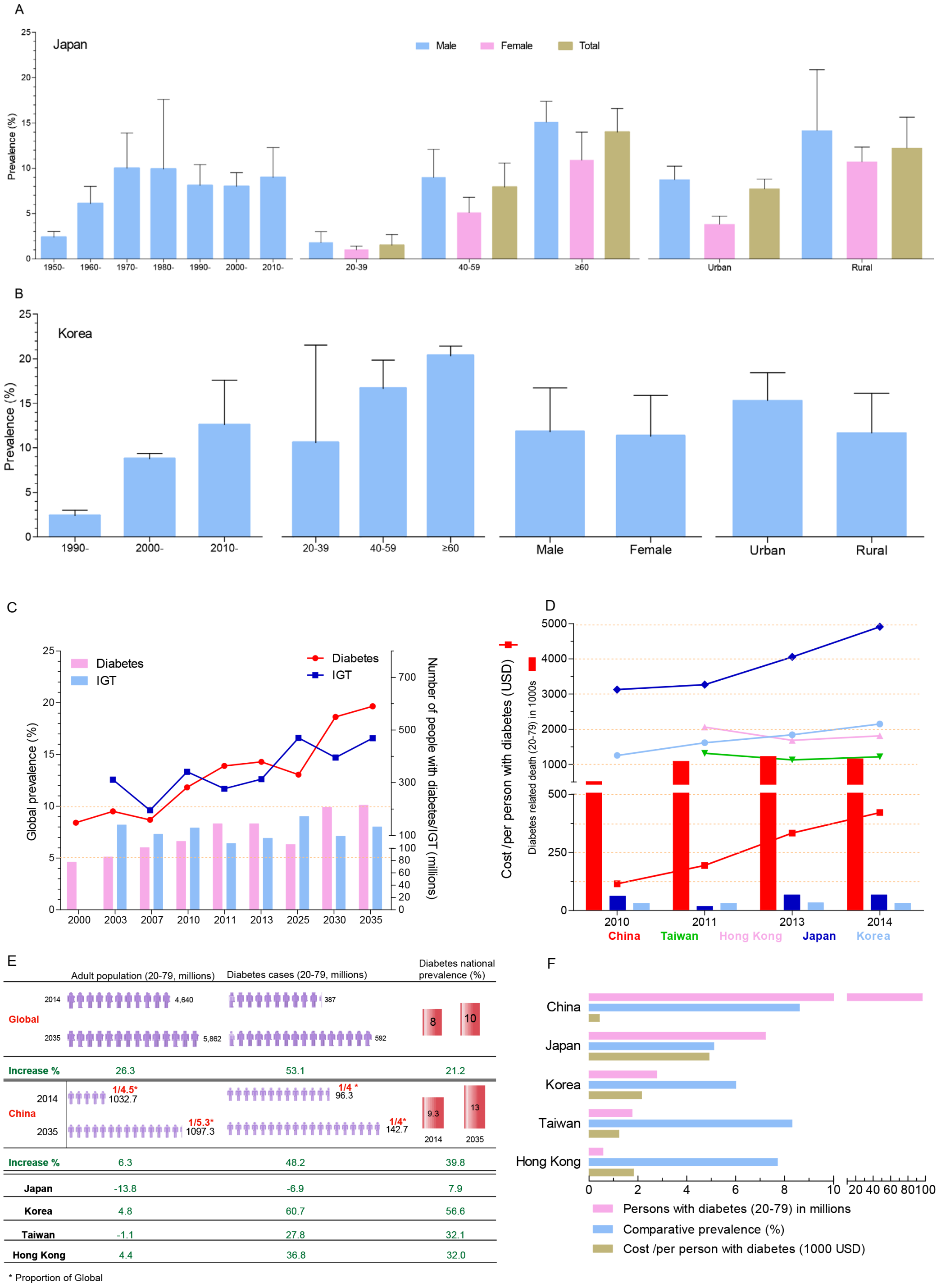

Supplementary Figure 6: Characteristics of T2DM in China over the past 35 years. (**A**) T2DM prevalence in Japan stratified by year, age, sex and region over past 35 years; (**B**) T2DM prevalence in Korea stratified by year, age, sex and region over past 35 years; (**C**) Global prevalence of diabetes/IGT (bars) and number of people with diabetes/IGT (line); (**D**) Health expenditures/person with diabetes (bars) and diabetes-related deaths (line); (**E**) Diabetes in China and the world in 2014 and 2035, as well as comparisons with Japan, Korea, Taiwan, and Hong Kong; (**F**) Comparison of diabetes prevalence, number of people with diabetes, and health expenditures per person with diabetes in China, Japan, Korea, Taiwan, and Hong Kong in 2014.

Supplementary Table 1: Characteristics of the identified studies of type 2 diabetes prevalence in China. See Supplementary_ Table_1

Supplementary Table 2: Characteristics of the identified studies of type 2 diabetes prevalence in Japan

First author	Year	Study location	Survey date	Location	Age (mean ± SD) (years)	Number of participants (male/female)	Number of cases (male/female)	Prevalence (%) total (male/female)
Kazufumi Nagahama [605]	2014	Japan (Okinawa)	2006	Urban	47.5	4812	130	2.70
Tadasumi Nakano [606]	2007	Japan	2002	Urban and Rural		127 million	7.4 million	
			1971–1980			610400	9737	1.60
			1981–1990			820305	11648	1.42
Shuka Umemura [607]	1988	Japan	1957–1980					
			1957	Urban		1204	24 (13/11)	2
			1958	Urban		1299	40 (22/18)	3.1
			1959	Urban		1410	32 (24/8)	2.3
			1960	Urban		1588	29 (17/12)	1.8
			1961	Urban		1777	45 (30/15)	2.5
			1962	Urban		1595	48 (35/13)	3
			1963	Urban		1538	84 (54/30)	5.5
			1964	Urban		1407	87 (61/26)	6.2
			1965	Urban		1459	88 (51/37)	6
			1966	Urban		1920	141 (90/51)	7.3
			1967	Urban		1458	153 (83/70)	10.5
			1968	Urban		1552	154 (87/67)	9.9
			1969	Urban		1549	138 (75/63)	8.9
			1970	Urban		1672	152 (87/65)	9.1
			1971	Urban		1925	168 (96/72)	8.7
			1972	Urban		1776	140 (71/69)	7.9
			1973	Urban		1532	151 (73/78)	9.9
			1974	Urban		1616	140 (76/64)	8.7
			1975	Urban		1905	218 (111/107)	11.4
			1976	Urban		2081	233 (118/115)	11.2
			1977	Urban		2221	271 (123/148)	12.2
			1978	Urban		2221	324 (176/148)	14.6
			1979	Urban		2302	343 (171/172)	14.9
			1980	Urban		2356	350 (212/138)	14.9
Akira Sekikwaa [608]	2000	Japan (Funagata)	1990–1992	Rural		2624 (1146/1478)	264 (104/160)	10.06 (9.1/10.8)
Yasushi Imamura [609]	2014	Japan	1991		30–79	8799 (5467/3332)	438 (328/110)	5.49 (6/3.3)
			1996			9909 (6258/3651)	721 (557/164)	7.28 (8.9/4.5)
			2001			10858 (6804/4054)	850 (680/170)	7.83 (10/4.2)
			2006			11566 (7146/4420)	953 (772/181)	8.24 (10.8/4.1)
			2011			10580 (6882/3705)	1133 (917/216)	10.71 (12/5.1)
Akihiko Uehara [610]	2014	Japan	2008.1–2012.12/ 2008.4–2013.3	Urban	20–69	55452 (47172/8280)	4049 (3776/273)	7.3 (8.0/3.3)
T. Ohmura [611]	1993	Japan (Hisayama)	1988	Rural	40–79	2490 (1077/1413)	272 (143/129)	10.92 (13.3/9.1)
Yoshihiko Takahashi [612]	2000	Japan (Miyako Island)	1998	Urban and Rural	≥45	2621 (1219/1402)	269 (154/115)	10.3 (12.6/8.2)
Saori Kashima [613]	2015	Japan (Tokyo)	1998.4–2006.3	Urban		34297 (17098/17199)	2354 (1619/735)	6.86 (9.5/4.3)
Naoko Mukai [614]	2014	Japan	1988/2002	Rural	40–79			
			1988			2490 (1077/1413)	308 (165/143)	12.4 (15.3/10.1)
			2002			2852 (1257/1595)	516 (302/214)	(24.0/13.4)

Supplementary Table 3: Characteristics of the identified studies of type 2 diabetes prevalence in Korea

First author	Year	Study location	Survey date	Location	Age (mean ± SD) (years)	Number of participants (male/female)	Number of cases (male/female)	Prevalence (%)
Sang Guk Kim [615]	2002	Namwon county, South Korea	1999.3–1999.7	Rural	40–49 (44.6 ± 3.0)	62	8 (5/3)	12.9
S.M.Kim [616]	2006	Korea	2001.11–2001.12	Urban/ Rural		5844 (2513/3331)	508 (241/267)	
Hye Young Lee [617]	2010	Korea	2010-	Urban	(63 ± 10)	520 (189/331)	80 (34/46)	
				Rural	(63 ± 8)	540 (219/321)	63 (34/29)	
Bo Kyung KOO [618]	2015	Korea	2001			4265 (1850/2415)	452 (205/246)	10.6 (11.1/10.2)
			2005			4633 (1979/2654)	385 (178/207)	8.3 (9.0/7.6)
			2010			5123 (2254/2869)	523 (264/252)	10.2 (11.7/8.8)
Yong Jun Choi [619]	2009	Korean	1998			5645	627	11.1
			2001			4154	370	8.9 (7.8/9.0)
			2005			2581716 (1415364/1166352)	234936 (144367/92142)	9.1 (10.2/7.9)
K-H.Song [620]	2007	Korea	1997	Rural	>30	1119 (424/695)	83 (30/53)	
			2003	Rural	>30	814 (316/498)	126 (51/75)	
Ie Byung Park [621]	2013	Korea	2003.1–2003.12		20–79	34989870	2694220	7.7
D.S.Lee [622]	2013	Korea	2005		≥35	3870	425 (225/200)	10.98
Hae-Rang Chung [623]	2009	Korean	2000-		≥20	5132 (2195/2937)	380 (161/219)	7.4 (7.3/7.5)
H-S Lee [624]	2013	Korea	2007–2008		≥30	9146	736 (334/402)	

Supplementary Table 4: T2DM prevalence in China over the last 35 years as well as in the next 10 years stratified by year

1980–1989		1990–1999		2000–2009		2010–2019		2020–2025*	
Year	Prevalence (%) (95% CI)	Year	Prevalence (%) (95% CI)	Year	Prevalence (%) (95% CI)	Year	Prevalence (%) (95% CI)	Year	Prevalence (%) (95% CI)
1980	0.8 (0.6–0.9)	1990	3.7 (2.7–4.7)	2000	6.1 (4.7–7.5)	2010	9.8 (8.6–11.0)	2020*	11.0
1981	0.5 (0.3–0.7)	1991	7.9 (4.9–11.0)	2001	5.8 (4.7–7.0)	2011	11.7 (9.9–13.6)	2021*	11.5
1982	0.7(0.3–1.0)	1992	3.0 (1.7–4.3)	2002	5.8 (5.2–6.5)	2012	6.9 (5.5–8.2)	2022*	11.7
1983	0.9 (0.5–1.2)	1993	4.5 (3.6–5.4)	2003	7.1 (5.4–8.8)	2013	9.5 (7.5–11.6)	2023*	11.9
1984	1.1 (0.7–1.4)	1994	2.8 (2.4–3.3)	2004	6.1 (4.8–7.5)	2014	9.3 (8.5–10.2)	2024*	12.4
1985	1.8 (0.7–2.9)	1995	3.6 (2.8–4.4)	2005	6.4 (5.3–7.5)	2015*	9.0	2025*	12.5
1986	1.1 (0.8–1.4)	1996	7.4 (4.9–9.9)	2006	8.7 (7.5–10.0)	2016*	11.1		
1987	1.4 (0.1–2.6)	1997	6.2 (5.1–7.4)	2007	8.1 (6.6–9.5)	2017*	10.5		
1988	1.7 (1.4–2.1)	1998	7.7 (5.5–9.8)	2008	8.4 (7.5–9.3)	2018*	10.9		
1989	5.0 (0.0–10.0)	1999	7.7 (5.5–10.0)	2009	8.0 (7.2–8.8)	2019*	11.4		

*Forecast using time-series analysis.

Supplementary Table 5: IGT prevalence in China over the last 35 years stratified by year period, age, gender, region, and BMI

Characteristic	IGT Prevalence % (95% CI)[a]			I^2 (%)			*P* Value for Heterogeneity			*P* Value[b]	*P* Value[c]
	Men	Women	Total	Male	Female	Total	Male	Female	Total		
Year period											
1980–1989	2.5 (0.7–5.4)	2.1 (0.6–4.6)	2.1 (1.5–2.8)	99.8	99.7	99.9	<0.001	<0.001	<0.001	<0.001	
1990–1999	5.4 (4.5–6.5)	4.9 (3.8–6.0)	4.6 (4.2–5.1)	98.5	98.7	99.2	<0.001	<0.001	<0.001	<0.001	<0.001
2000–2009	6.4 (5.1–7.8)	6.0 (4.5–7.6)	6.6 (5.7–7.5)	98.4	98.7	99.2	<0.001	<0.001	<0.001	0.040	
2010–2014	11.2 (6.6–16.9)	10.0 (5.4–15.7)	13.6 (10.2–17.3)	98.7	99.3	99.3	<0.001	<0.001	<0.001	0.910	
Age											
15–19											
20–29	4.1 (1.8–7.2)	2.1 (0.8–3.7)	1.7 (1.1–2.4)	96	92.2	95.5	<0.001	<0.001	<0.001	0.140	
30–39	5.0 (3.5–6.8)	2.6 (1.7–3.8)	3.1 (2.5–3.8)	95.9	92.2	96.2	<0.001	<0.001	<0.001	0.940	
40–49	5.0 (3.4–6.9)	4.8 (3.3–6.7)	5.6 (4.8–6.4)	96.7	96.1	97.5	<0.001	<0.001	<0.001	0.090	<0.001
50–59	6.4 (4.4–8.7)	7.1 (5.1–9.3)	8.1 (6.9–9.5)	97.0	95.6	98.2	<0.001	<0.001	<0.001	0.320	
60–69	7.0 (4.2–10.5)	7.7 (5.0–11.0)	9.9 (8.3–11.5)	96	95.3	97.5	<0.001	<0.001	<0.001	0.130	
70–79	7.0 (0.3–21.1)	9.6 (0.3–29.6)	11.2 (9.0–13.6)	98.2	98.8	95.5	<0.001	<0.001	<0.001	0.810	
≥80	13.3 (4.8–25.3)	15.2 (6.1–27.5)	12.1 (9.5–14.8)	73.5	73.1	89.9	<0.001	<0.001	<0.001	0.100	
Region											
Urban	7.9 (6.5–9.5)	6.8 (5.4–8.4)	7.6 (6.6–8.5)	98.7	98.8	99.3	<0.001	<0.001	<0.001	0.220	<0.001
Rural	6.5 (3.7–10.2)	7.4 (3.5–12.5)	5.8 (4.7–7.0)	97.8	99.1	98.6	<0.001	<0.001	<0.001	<0.001	
BMI											
<18.5			3.7 (2.3–5.5)			69.1			0.006		
18.5–23.9			5.1 (4.4–5.9)			82.3			<0.001		<0.001
24–27.9			12.6 (9.6–16.0)			96.9			<0.001		
≥28.0			13.8 (10.9–17.0)			88.0			<0.001		

[a]Pooled estimates of T2DM prevalence within the strata of each study characteristic are indicated. [b, c]Difference between genders in each stratum and the effect of different stratum on the evaluation of prevalence by using chi-squared (χ^2) test, respectively.

Supplementary Table 6: T2DM prevalence reported in the 6th edition of the "IDF DIABETES ATLAS"

	Country/ territory	Adult population (20-79) in 1000s	Diabetes cases (20-79) in 1000s	Undiagnosed diabetes cases (20–79) in 1000s	Diabetes national prevalence (%)	Diabetes comparative prevalence (%)	Diabetes-related deaths (20–79)	Diabetes-related deaths under 60, (%)	Cost /per person with diabetes (USD)	One in X adults has diabetes
2000		847913	22564.8		2.7					
2010		964301.6	43157.2		4.5	4.2	574575		R=2, 115	
2011		968974.93	90045.09	51217.65	9.29	8.8	1133918		194	
2013	China	1023050.4	98407.4	53238.4	9.62	9.02	1271003		333	
2014		1032731	96288	51273.4	9.3	8.6	1205923	41.1	421.3	11
2035		1097309.1	142663.2		13	9.5				
2000		5067	614.4		12.1					
2010		5732.5	586.6		10.2	8.5				
2011		5603.57	525.39	245.41	9.38	7.61			2059	
2013	Hong Kong	5679.9	540	266.8	9.51	7.5			1679	
2014		5729.8	568.4	306.6	9.9	7.7			1811.4	10
2035		5979.7	781.3		13.07	7.5				
2000										
2010		14221.6	815.9		5.7	7.5				

2011		17355.82	1664.54	777.5	9.59	8.28			1314	
2013	Taiwan	17605.4	1721.1	850.2	9.78	8.3			1129	
2014		17717.2	1757.1	947.9	9.92	8.3			1219.3	10
2035		17548.1	2298.9		13.1	8.3				
2000		95849	7113.2		7.4					
2010		96665.9	7089.2		7.3	5	59429		3125	
2011		95340.66	10674.32	4985.98	11.2	7.71	14857		3266	
2013	Japan	95304.4	7203.8	3558.7	7.56	5.1	64680		4054	
2014		94909.2	7212.1	3890.9	7.6	5.1	64715	26.4	4908.1	13
2035		81751.4	6722.5		8.22	5.2				
2000		32565	1986		6.1					
2010		36602.9	3292.4	9	7.9		28808		1255	
2011		36204.09	3186.39	1488.36	8.8	7.53	28912		1615	
2013	Korea	37365.7	3323.9	1642	8.9	7.5	30836		1839	
2014		37736.7	2767.7	1493.2	7.3	6	27805	37.3	2144.4	14
2035		39468.8	4511.2		11.43	7.5				

Supplementary Table 7: Economic data from the international monetary fund

	Total population (million)					Urban population as % of total population				GDP (Current Prices, U.S. dollars) (billion)				
	1990	2000	2010	2014*	2019**	1960	1980	2000	2013	1980	1990	2000	2010	2014
China	1143	1267	1341	1368	1402	16	19	36	53	309	404	1193	5950	10380
Japan	123	127	128	127	128	43.1	76	79	92	1087	3104	4731	5495	4616
Korea	43	47	49	50	52	27.7	57	80	82	65	279	562	1094	1417
United States	250	282	310	319	330	70	74	79	81	2862	5980	10284	14964	17419

*Estimate **Forecast.*

Supplementary Table 8: Analyses of meta-regression for T2DM prevalence

Characteristic	*P* Value for meta-regression[a]		explanation for heterogeneity, %	
	P_g	*P*	Gender	Total[b]
Year period	0.431	<0.001	–0.04	13.8
Age	0.945	<0.001	–0.14	46.7
Region	0.032	<0.001	0.24	3.21
BMI	0.667	<0.001	–0.26	40.51
Diagnostic criteria		0.437		–0.13

[a] Represents the test for significance of the effect across strata; [b] Represents the proportion of between-study variance explained by each characteristic. P_g: *P* value of different gender (male/female); *P*: *P* value of meta-regression for each characteristic.

Supplementary Table 9: Analyses of publication bias for T2DM prevalence

Characteristic	Subgroup	Begger's Test		Eegger's Test
		Z Value	*P* Value	*P* Value
Year period	1980–1989	2.33	0.020	0.005
	1990–1999	2.56	0.010	<0.001
	2000–2009	21.75	<0.001	<0.001
	2010–2014	3.96	<0.001	0.922
Age	15–19	1.36	0.174	0.354
	20–29	2.95	0.003	<0.001
	30–39	5.76	<0.001	<0.001
	40–49	1.51	0.131	<0.001
	50–59	0.58	0.560	<0.001
	60–69	1.74	0.081	<0.001
	70–79	0.34	0.732	<0.001
	≥80	0.38	0.707	0.281
Region	Urban	3.80	<0.001	<0.001
	Rural	10.84	<0.001	0.001
BMI	<18.5	3.76	<0.001	<0.001
	18.5–23.9	1.10	0.273	<0.001
	24–27.9	1.35	0.177	<0.001
	≥28	2.07	0.039	<0.001
Diagnostic criteria	CDS	1.39	0.166	0.588
	ADA	0.75	0.454	0.009
	WHO	3.71	<0.001	0.331
	FPG	2.46	0.014	0.531
	FPG+OGTT	2.35	0.019	0.872

CDS: Chinese Diabetes Society, ADA: American Diabetes Association, WHO: World Health Organization, IDF: International Diabetes Federation, FPG: fasting plasma glucose (FPG ≥ 7.0mmol/L), FPG+OGTT: FPG (FPG ≥ 7.0mmol/L) or/and oral glucose tolerance test (OGTT ≥ 11.1mmol/L).
