# Supplementary material for: Type 2 diabetes epidemic in East Asia: a 35–year systematic trend analysis
Source: Oncotarget. 2017 Dec 6;9(6):6718–27. doi: 10.18632/oncotarget.22961 (PMC5805508; doi:10.18632/oncotarget.22961)
Supplement: Supplementary file 2 [file oncotarget-09-6718-s002.docx]

**Supplementary Table 1: Characteristics of the identified studies of type 2 diabetes prevalence in China.**

| **First author** | **Year** | **Study location** | **Survey date** | **Location** | **Age (mean±SD) (years)** | | **Number of participants (male/female)** | **Number of cases (male/female)** | **Prevalence (%) total (male/female)** |
| --- | --- | --- | --- | --- | --- | --- | --- | --- | --- |
| Xueyun Hu[5] | 2013 | Anhui (Tongcheng) | 2010-2012 | Urban | 20-103 | | 47029 | 1012 | 2.15 |
| Ying Ding[6] | 2013 | Anhui (Hefei) | 2011.7.19-2011.8.31 | Urban | 40-89 (56.4±10.8) | | 2860 (1093/1767) | 400 (172/228) | 14 (15.7/12.9) |
| Lizhuan Yin[7] | 2012 | Anhui(Hefei) | 2011.3-2011.7 | Urban | ≥40 | | 3124(1168/1956) | 472(210/262) | 15.11(17.98/13.39) |
| Yuanquan Liao[8] | 2013 | Anhui (Wannan district) | 2011.1-2011.6 | Urban | ≥20 | | 3799 (2136/1663) | 300 | 7.9 |
| Wei Xu[9] | 2012 | Anhui (Maanshan) |  | Urban and Rural | 18-98 | | 600(204/396) | 79(33/46) | 13.1(16.18/11.62) |
| Wei Wang[10] | 2011 | Anhui | 2006 | Rural | 40-70 | | 1248(438/810) | 112(51/61) | 9.0(11.6/7.5) |
| Yingchun Li[11] | 2006 | Anhui | 2004.6-8 | Urban | ≥28 | | 785(352/433) | 59(34/25) | 7.5(9.7/5.8) |
| Deren Hou[12] | 2003 | Anhui (Hefei) | 2000 and 2002 | Urban |  | |  |  |  |
|  |  |  | 2000 | Urban |  | | 1193(876/317) | 157 | 13.16 |
|  |  |  | 2002 | Urban |  | | 1468(1071/397) | 261(184/77) | 17.78(17.18/19.4) |
| Yongqi Huang[13] | 1996 | Anhui(Bengbu) | 1980 and 1995 | Urban and Rural |  | |  |  |  |
|  |  |  | 1980 |  | ≥15 | | 16844 | 144 | 0.85 |
|  |  |  | 1995 |  | ≥25 | | 2282 | 60 | 2.63 |
| Changyu Pan[14] | 2003 | Beijing | 1996.5-2000.6 | Urban | 70.6±6.0 | |  |  |  |
|  |  |  | 1996 |  |  | | 1828 (1716/112) | 324 (304/20) | 17.1 (17.7/17.8) |
|  |  |  | 1997 |  |  | | 2207 (2065/142) | 532 (504/28) | 24.1 (24.4/19.7) |
|  |  |  | 1998 |  |  | | 2133 (1991/142) | 596 (567/29) | 28.0 (28.4/20.4) |
|  |  |  | 1999 |  |  | | 2077 (1935/142) | 579 (545/34) | 27.9 (28.1/23.9) |
|  |  |  | 2000 |  |  | | 2058 (1917/141) | 589 (560/29) | 28.7 (29.2/20.6) |
| Junjing An[15] | 2013 | Beijing (Fengtai) | 2010.8-2010.10 | Urban | 20-90 (50.18±15.51) | | 853 (384/469) | 113 (54/59) | 13.2 (14.1/12.6) |
|  |  |  | 2002 |  | ≥20 | | 417 | 56 | 13.4 |
| Shubo Li[16] | 2013 | Beijing (Changping) |  | Urban | 18-80 | | 13711 (6006/7705) | 1348 (624/724) | 9.83 (10.39/9.40) |
|  |  |  | 2002 |  |  | | 912(441/471) | 50 (20/30) | 5.48 (4.54/6.37) |
|  |  |  | 2005 |  |  | | 2991 (1169/1822) | 332 (143/189) | 11.10 (12.23/10.37) |
|  |  |  | 2008 |  |  | | 1724 (777/947) | 108 (71/37) | 6.26 (9.14/3.91) |
|  |  |  | 2010 |  |  | | 8084 (3619/4465) | 858 (390/468) | 10.61 (10.78/10.48) |
| Qinghua Wang[17] | 2013 | Beijing | 2012 | Urban | 20-98 | | 3767 (1864/1903) | 266 (158/108) | 7.0 (8.5/5.7) |
| Li Li[18] | 2012 | Beijing |  | Urban | ≥26 | | 1035(730/706) | 123(67/57) | 11.88(9.18/8.07) |
| Lei Qiao[19] | 2010 | Beijing | 2007.10-2007.12 | Urban | ≥18 | | 419(200/219) | 8 | 1.9 |
| Fang Liu[20] | 2012 | Beijing |  | Urban |  | |  |  |  |
|  |  |  | 2007 |  | 35-85 | | 2408(2132/276) | 109(91/18) | 4.5(4.3/6.5) |
|  |  |  | 2009 |  | 35-85 | | 2408(2132/276) | 149(119/30) | 6.2(5.6/10.9) |
| Yiqiang Zhan[21] | 2010 | Beijing | 2007.5-8 | Urban and Rural | ≥20 | | 10054(3687/6367) | 330(124/206) | 16.03(18.70/14.76) |
| Xianghai Zhou[22] | 2009 | Beijing | 2007.10-2008.5 | Urban and Rural | 35-79 | | 2801(763/2038) | 580(185/395) | 20.7(24.2/19.4) |
| Suqin Ding[23] | 2012 | Beijing | 2010 | Urban | >18 | | 21272(9966/11306) | 2142 | 10.1(10.1/10.1) |
| Ying Su[24] | 2010 | Beijing | 2007 | Urban | 24-81(52.0±11.1) | | 525(145/380) | 52(11/41) | 9.9(7.6/10.8) |
| Haixiang Wang[25] | 2010 | Beijing (Shunyi) | 2008 | Urban | ＞18 | | 826(369/357) | 82 | 9.93(11.65/8.53) |
| Guixia Zhang[26] | 2006 | Beijing (Shunyi) | 2006.1-2006.5 | Urban | ＞18 | | 3000(1164/1836) | 166(84/82) | 5.53(7.22/4.47) |
| Danjie Ruan[27] | 2010 | Beijing (Huairou) | 2007.7-2008.8 | Urban and Rural | 20-70 | | 1797(764/1033) | 184(82/102) | 10.2(10.7/9.9) |
| Xiuyun Han[28] | 2012 | Beijing(Huairou) | 2011 | Urban/Rural | ≥25 | | 5017 | 261 | 5.2 |
| Wei Heng[29] | 2010 | Beijing | 2008 | Urban | ＞60 | | 342 | 18 | 5.3 |
| Wei Wang[30] | 2012 | Beijing |  | Urban | ≥15 | | 6950(3380/3570) | 611(284/327) | 8.79(8.4/9.16) |
| Jingyuan Jia[31] | 2012 | Beijing | 2008 | Urban and Rural | 18-69 | | 2773(1237/1536) | 305 | 11.0(12.29/9.96) |
| Xingguang Zhang[32] | 2011 | Beijing | 2010.04-2010.06 | Urban | 60-99(77.6±7.4) | | 2115(1998/117) | 457 | 21.61 |
| Xin Gao[33] | 2011 | Beijing(xuanwu) | 2009.09-2010.01 | Urban | ≥60(69.6±6.3) | | 812(279/515) | 193(70/123) | 23.77(13.59/44.08) |
| Jun Li[34] | 2011 | Beijing | 2009 | Urban | 20-78 | | 4332(1477/2855) | 466 | 10.8 |
| Yu Wang[35] | 2011 | Beijing | 2010.4 | Rural | 13-95 | | 5310(2440/2870) | 277 | 5.2 |
| Shanshan Li[36] | 2008 | Beijing | 2004-2005 | Urban |  | | 1161 | 87 | 7.5 |
| Weidong Xing[37] | 2008 | Beijing (Changping) | 2006 | Urban | ≥18 | | 170(66/104) | 16(4/12) | 9.4(6.1/11.5) |
| Baoheng Wu[38] | 2008 | Beijing (Daxing) | 2007 | Rural | 20-107 | | 4980(1995/2985) | 147(41/106) | 3.0(2.0/3.6) |
| Chenhui Bao[39] | 2008 | Beijing (Xicheng) | 2007 | Urban | ≥25 | | 2169(1068/1101) | 226(110/116) | 10.5(10.4/10.6) |
|  |  |  | 2001 | Urban | ≥25 | | 2084(895/1189) | 145(52/93) | 7.0(5.8/7.8) |
| Jingyuan Jia[40] | 2007 | Beijing(Haidian) | 2005.10-2005.11 | Urban | ≥18 | | 2682(938/1744) | 259(104/155) | 9.66(11.09/8.89) |
| Guiru Chen[41] | 2007 | Beijing(Tongzhou) | 2006.6 | Rural | 40.69 | | 221(133/88) | 21(19/2) | 9.5(14.3/2.3) |
| Liu Yang[42] | 2009 | Beijing | 2001-2007 | Urban | ≥25 | | 6466(2940/3526) | 950 | 14.7 |
|  |  |  | 2001 | Urban | ≥25 | | 2109(904/1205) | 146(52/94) | 6.9(5.8/7.8) |
|  |  |  | 2004 | Urban | ≥25 | | 2188(968/1220) | 173(81/89) | 7.9(8.4/7.3) |
|  |  |  | 2007 | Urban | ≥25 | | 2169(1068/1101) | 226(110/116) | 10.4(10.3/10.5) |
| Jianping Xing[43] | 2009 | Beijing | 2007.7-9 | Rural | 52.5±9.5(53.1±9.5/52.2±9.5) | | 3294(1173/2121) | 499(188/311) | 15.1(16.0/14.7) |
| Heling Yang[44] | 2009 | Beijing | 2006.5-10 | Rural | ≥25 | | 3588 | 186 | 5.2 |
| Kai You[45] | 2009 | Beijing | 2008.11 | Rural | 18-79(44.24±13.28) | | 827(369/458) | 82(43/39) | 9.92(11.65/8.52) |
| Linan Chu[46] | 2005 | Beijing(Xi Cheng) | 2002.11 | Urban | ≥25 | | 2109(898/1199) | 145(52/93) | 6.9(5.8/7.8) |
| Yingxin Wei[47] | 2011 | Beijing (Tongzhou) |  | Rural | ≥25 | | 2306 | 109 | 4.7 |
| Yue Xing[48] | 2009 | Beijing | 2007.9 | Urban | 21-83 | | 1414(696/718) | 61(38/23) | 4.31(5.46/3.20) |
| C.Y.Pan[49] | 1996 | Beijing | 1992.5-1993.2 | Urban | 30-64 | | 29859 | 955 | 3.2 |
| Yaou Sun[50] | 2003 | Beijing | 1998.1-1999.12 | Urban | ≥55 | | 977(902/75) | 101(97/4) | 10.2(10.8/5.3) |
| Chenhui Bao[51] | 2005 | Beijing(Xicheng) | 2003 | Urban | ≥15 | | 1017(494/523) | 80(36/44) | 7.9(6.7/8.8) |
| Meiling Chen[52] | 1999 | Beijing | 1996 | Urban | >50 | | 1954 | 217(206/11) | 11(11.4/7.1) |
| Shulan Shen[53] | 1999 | Beijing | 1990.6-1999.6 | Urban |  | |  |  |  |
|  |  |  | 1990 | Urban |  | | 1722 | 106 | 5.9 |
|  |  |  | 1991 | Urban |  | | 1773 | 110 | 6.2 |
|  |  |  | 1992 | Urban |  | | 2253 | 165 | 7.3 |
|  |  |  | 1993 | Urban |  | | 2260 | 183 | 8.1 |
|  |  |  | 1994 | Urban |  | | 2367 | 227 | 9.6 |
|  |  |  | 1995 | Urban |  | | 2464 | 288 | 11.7 |
|  |  |  | 1996 | Urban |  | | 2820 | 358 | 12.7 |
|  |  |  | 1997 | Urban |  | | 2808 | 309 | 11.0 |
|  |  |  | 1998 | Urban |  | | 2798 | 345 | 12.3 |
|  |  |  | 1999 | Urban |  | | 3041 | 510 | 16.7 |
| Yangze[54] | 2001 | Beijing | 1997 | Urban and Rural | 40-99 (56) | | 2210(864/1346) | 256 | 11.58(9.72/12.78) |
| Guo Qiyu[55] | 2002 | Beijing |  | Urban | ≥40 | | 2050 | 192 | 9.4 |
| Susu Wang[56] | 2002 | Beijing | 2001.5 | Urban | 40~91 (62.62) | | 2619(2297/322) | 307 | 11.72 |
| Xiaolin Zhang[57] | 2001 | Beijing | 1997-2000 |  | ≥35 | | 4144 |  |  |
|  |  |  | 1997 | Urban |  | | 4144 | 405(202/203) | 9.77 |
|  |  |  | 1998 | Urban |  | | 4144 | 443 | 10.69 |
|  |  |  | 1999 | Urban |  | | 4144 | 487 | 11.75 |
|  |  |  | 2000 | Urban |  | | 4144 | 531 | 12.81 |
| Zuqiang Zhou[58] | 1999 | Beijing | 1997.5 | Urban |  | | 2087(1293/794) | 27 | 1.3 |
| Caixia Liu[59] | 2008 | Beijing(Huairou) | 2005.09-2005.10 | Rural | ≥18(44.36 ±13.09) | | 374(163/211) | 39 (22/17) | 10.5(13.5/8.2) |
| Minxu Kuang[60] | 1999 | Beijing | 1999 | Urban | ≥30 | | 2080(1188/892) | 66(51/15) | 3.17（4.29/1.68） |
| Yongcun Zhen[61] | 1999 | Beijing | 1999 | Urban | ≥40 | | 1516 | 103 | 6.79 |
| Zunyong Liu[62] | 1997 | Beijing | 1995.7 | Rural | 20-74 | | 1259 |  |  |
|  |  |  | 1980 |  |  | | 1259 | 8 | 0.67 |
|  |  |  | 1985 |  |  | | 1259 | 11 | 0.88 |
|  |  |  | 1990 |  |  | | 1259 | 41 | 3.26 |
|  |  |  | 1995 |  |  | | 1259 | 51 | 4.04 |
| Zengjin Li[63] | 1996 | Beijing | 1991.5-1991.10 | Urban and Rural | ≥60 | | 960 | 57 | 5.94 |
| Ninghua Li[64] | 1990 | Beijing | 1979 | Urban |  | | 525 | 23 | 4.38 |
|  |  |  | 1988 | Urban |  | | 326 | 26 | 7.98 |
| Yanna Chen[65] | 2008 | Beijing | 2005 | Urban | 18-84 | | 607(325/282) | 32 | 5.3 |
| Rong Wang[66] | 2008 | Beijing |  | Urban | 45-80(62.17±7.08) | | 149(61/88) | 31 | 20.81 |
| Jingyuan Jia[67] | 2008 | Beijing(Haidian) |  | Urban | ≥60 | | 439(154/285) | 92(38/54) | 20.96(24.7/18.9) |
| Wuxi Wang[68] | 2013 | Chongqing | 2011.5-2012.3 | Urban | ≥20 | | 10932 (4985/5947) | 447 | 4.09 |
| Xingbi Li[69] | 2013 | Chongqing (Fengdu) | 2011.11.15-22 | Urban and Rural | 18-91 (46.6±14.6) | | 600 (187/413) | 59 (18/41) | 9.83 (9.63/9.93) |
| Ju Fu[70] | 2013 | Chongqing | 2010.1-2012.6 | Urban | 62-84 (76.1±1.5) | | 692 | 70 | 10.12 |
| Binlu Sun[71] | 2011 | Chongqing | 2011.11 | Urban | ≥20 | | 1544(723/821) | 106 | 6.87 |
| Chuanming Xiang[72] | 2011 | Chongqing | 2011.4-2011.6 | Urban | 20-95 | | 3657(2790/6447) | 820(558/262) | 12.72 |
| Damei Liu[73] | 2009 | Chongqing |  | Urban | 50-55 | | 4640 | 119 |  |
| Xianfeng Liu[74] | 2007 | Chongqing |  | Urban and Rural | ≥18 | | 1415 | 71 | 5 |
| Haiyan Liao[75] | 2007 | Chongqing |  | Urban | ≥18 | | 940(562/378) | 35(19/16) | 3.72(3.38/4.23) |
| Xiaojun Tang[76] | 2009 | Chongqing |  | Urban | ≥20 | | 2966(1720/1246) | 300(183/117) | 10.11(10.64/9.39) |
| Xiaojun Tang[77] | 2006 | Chongqing | 2002.1-7 | Urban | ≥20 | | 3717(2217/1500) | 363(227/136) | 9.77（10.19/9.07） |
| Fashu Luo[78] | 2002 | Chongqing(Beipei) | 1999.1~2000.6 | Urban | ≥60 | | 4893(2318/2575) | 491(204/287) | 10.03(8.800/11.145) |
| Lixin Cai[79] | 2014 | Fujian (Xiamen) | 2013.4-2013.5 | Urban | 34.6±12.2 (34.5±12.1/34.7±12.4) | | 3782 (1922/1860) | 199 (118/81) | 5.26 (6.14/4.35) |
| Lufang Chen[80] | 2014 | Fujian (Xiamen) | 2012.11-2013.1 | Urban | 65-93 (71.09±5.08) | | 908 (370/538) | 249 (95/154) | 27.4 (25.7/28.6) |
| Shaoyong Xu[81] | 2013 | Fujian and Shǎnxi | 2007-2008 | Urban | ≥20 | | 5926 (3254/2672) | 568 | 9.6 |
|  |  | Shǎnxi |  |  | 43.6±13.7 | | 3254 (1366/1888) | 267 | 8.2 |
|  |  | Fujian |  |  | 43.7±14.2 | | 2672 (1056/1616) | 301 | 11.3 |
| Hangsheng Li[82] | 2013 | Fujian (Yongan) |  | Urban | ≥18 | | 3000 (1589/1411) | 201 (101/100) | (6.4/7.1) |
| Hong Li[83] | 2009 | Fujian(Fuzhou) | 2007.7-2007.11 | Urban and Rural | ≥60 | | 4237(1828/2409) | 475(214/261) | 11.21(11.71/10.83) |
| Tongbao Zhu[84] | 2002 | Fujian(Xiamen) | 1995~2000 | Urban | ≥60 | | 572(365/207) | 28(22/6) | 4.8 |
| QingheWang[85] | 2003 | Fujian(Guliangyu) | 2000.5 | Urban | ＞20 | | 983 | 35 | 3.56 |
| Diabetes Group[86] | 1997 | Fujian | 1980 and 1995 | Urban and Rural | ≥15 | |  |  |  |
|  |  |  | 1980 |  | ≥15 | | 30742 | 333 | 1.08 |
|  |  |  | 1995 |  | ≥15 | | 4075 | 83 | 2.04 |
| Faqin Yang[87] | 1996 | Fujian(Putian) | 1994.8-1994.11 | Urban and Rural | ≥25 | | 2419(934/1485) | 69(32/37) | 2.85(3.43/2.49) |
| Wei Wang[88] | 2014 | Gansu (Pingliang) | 2012.9-2012.11 | Urban | ≥18 | | 3252 (1295/1957) | 161 | 4.95 |
| Linglong Zhao[89] | 2014 | Gansu (Lanzhou) | 2012.12.1-2012.12.31 | Urban and Rural | ≥18 | | 16753 (8353/8400) | 587 (291/296) | 3.5 (3.48/3.52) |
| Zhengqin Wei[90] | 2013 | Gansu (Lanzhou) | 2012.3.1-2012.5.30 | Urban | ≥18 | | 5198 (1891/3307) | 403 (145/259) | 7.75 (7.67/7.83) |
| Chunmei Han[91] | 2014 | Gansu (Wuwei) | 2011.11-2012.11 | Urban | 22-79 (50.5±16.9) | | 534 (346/188) | 30 (23/7) | 5.62 (6.65/3.72) |
| Changqing Yang[92] | 2013 | Gansu (Tianzhu) | 2011.9-2012.6 | Urban and Rural | 20-74 | | 846 (402/444) | 42 (21/21) | 4.96 (5.22/4.73) |
| Suping Zhu[93] | 2013 | Gansu | 2007 | Urban and Rural | ≥35 | | 5143 | 357 |  |
| Lijiang Liu[94] | 2013 | Gansu (Lanzhou) | 2007.7-2007.8 | Rural | ≥35 | | 1873 (788/1085) | 94 (45/49) | 5.0 (5.7/4.5) |
| Xiong Yue[95] | 2010 | Gansu (Lanzhou) |  | Urban and Rural | ≥15 | | 2466 (1089/1377) | 45 (25/20) | 1.825 (2.296/1.452) |
| Yuande Chen[96] | 2013 | Gansu (Qingcheng) | 2012.4-2012.5 | Urban and Rural | ≥18 | | 5231 | 243 | 4.65 |
| Qin Wang[97] | 2013 | Gansu (Gaolan) | 2012.6.10-24 | Rural | ≥18 | | 5610 | 72 (45/27) | 1.3 (0.80/0.49) |
| Xingxiang Li[98] | 2010 | Gansu | 2009.6-2010.3 | Urban | 63.84 ±7.77 | | 442 (317/125) | 48 (37/11) | 10.86 (11.67/8.80) |
| Xiuzhen Wang[99] | 2011 | Gansu(zhangye) | 2008.1-2009.6 | Urban | ≥20 | | 12 395(7273/5122) | 994(618/376) | 8.0(8.2/7.8) |
| Yujue He[100] | 2003 | Gansu(Lanzhou) | 2002.3~2002.4 | Urban | ≥18 | | 2179 | 133(64/69) | 6.1 |
| Lin Gao[101] | 1998 | Gansu(Lanzhou) | 1997.9-1997.12 | Urban and Rural | ≥40 | | 2580(1353/1227) | 81(39/42) | 3.14(2.88/3.42) |
| Hongjuan Wang[102] | 2013 | Guizhou (Guiyang) |  | Urban-worker | 20-59 | | 435 | 12 | 2.7 |
|  |  |  |  | Urban-civil servant | | | 395 | 23 | 5.8 |
| Kun Chen[103] | 2014 | Guizhou (Tongren) | 2011.9-2012.12 | Urban | | 20-86 | 4252 (2536/1716) | 173 (138/35) | 4.07 (5.44/2.04) |
| Jiangping Zhang[104] | 2013 | Guizhou (Guiyang) | 2010.10-2010.12 | Urban | | ≥18 | 891 | 57 | 6.4 |
| Kaijian Luo[105] | 2007 | Guizhou(Guiyang) | 2005.8-2006.8 | Urban | |  | 3807(2311/1496) | 261(168/93) | 6.85(7.27/6.22) |
| Diabetes Group[106] | 1981 | Guizhou(Guiyang) |  | Rural | | ≥20 | 1335/1322 | 9/6. | 0.32/2.18 |
| Juxuan Guo[107] | 2014 | Guangdong (Guangzhou) | 2010 | Urban | | 25-34 | 635 (265/370) | 17 (6/11) | 2.68 (2.26/2.97) |
| Qiujiao Wu[108] | 2014 | Guangdong (Guangzhou) |  | Urban | | ≥15 (67.7) | 1915 (905/1007) | 85 (34/51) | 4.4 (3.8/5.1) |
| Ke Li[109] | 2013 | Guangdong (Guangzhou) | 2008-10-2008.12 | Urban and Rural | | 15-69 | 6987 (3264/3723) | 300 (121/187) | 4.3 (3.7/5.0) |
| Ying Cao[110] | 2010 | Guangzhou | 2008.4-2008.5 | Urban | | ≥18 | 1532(697/835) | 130(60/70) | 8.48(8.61/8.38) |
| Qingxiang Zhang[111] | 2012 | Guangdong(Guangzhou) | 2010 | Urban | |  | 43384 | 2958 | 6.82 |
| Bingying Pan[112] | 2006 | Guangdong (Guangzhou) | 2004.4-2004.8 | Urban | | >20 | 6505(2636/3869) | 481(203/278) | 7.4(7.7/7.2) |
| Yongzhou Liu[113] | 2008 | Guangdong (Guangzhou) |  | Urban | |  |  |  | 3.2 |
| Kaixing Zhu[114] | 2007 | Guangdong (Guangzhou) | 2005.1-2005.6 | Urban | |  | 2017(807/1210) |  | 9.0(8.1/9.7) |
| Juexin Hong[115] | 2007 | Guangdong (Guangzhou) | 2005.9-2005.10 | Urban | |  | 535(226/309) | 29(10/19) | 5.1(4.4/6.2) |
| Jianwen Fan[116] | 2011 | Guangdong (Guangzhou) | 2009.8-10 | Urban | | ≥15 | 33300(16157/17143) | 1388(573/815) | 4.2(3.5/4.8) |
| Yuanli Chen[117] | 2011 | Guangdong (Sanshui) |  | Urban and Rural | | ≥20 | 19119(9325/9794) | 1403(802/601) | 7.34(8.60/6.50) |
| Xiaohui Wu[118] | 2014 | Guangdong (Shaoguan) | 2012.9-2013.5 | Urban | | ≥25 | 1794 (924/870) | 174 (95/79) |  |
| Yanfang Guo[119] | 2014 | Guangdong (Shenzhen) | 2009 | Urban | | 18-69 (38.6±11.5) | 3154 (1390/1764) | 152 (79/73) | 4.82 (5.68/4.14) |
| Yan Zhao[120] | 2013 | Guangdong (Shenzhen) | 2009.1-2009.12 | Urban | | 20-60 | 9268 (5572/3696) | 451 | 4.87 |
| Shengyuan Liu[121] | 2012 | Guangdong (Shenzhen) | 2012.3-2012.5 | Urban | | ≥15(42.95±11.08) | 1586(678/908) | 59(33/26) | 3.72(2.08/1.64) |
| Yufang Chen[122] | 2011 | Guangdong (Shenzhen) | 2008 | Urban | | 21-60 | 797(379/418) | 18(14/4) | 3.1(3.7/1.0) |
| Jingang Deng[123] | 2008 | Guangdong (Shenzhen) | 2007 | Urban | | 30-86(45.2±13.4) | 4573(2321/2252) | 144(70/74) | 3.15 |
| Jun Chen[124] | 2009 | Guangdong (Shenzhen) | 2007 | Urban | | 60-89 | 10220(5820/4400) | 2060 | 20.2(20.3/20.1) |
| Yunhu Chen[125] | 2014 | Guangdong (Qingyuan) | 2010.1-2012.12 | Urban | | ≥60 | 7314 (3929/3385) | 1335 (619/716) | 18.25 (15.75/21.15) |
| Chusheng Li[126] | 2014 | Guangdong (Qingyuan) | 2012.2-2012.9 | Rural | | ≥35 | 7382 (3784/3598) | 379 (216/163) | 4.70 (3.94/3.20) |
| Shan Hao[127] | 2013 | Guangdong (Zhaoqing) | 2012.1-2013.1 | Urban | | 40-64 (50.4±2.1) | 1268 (656/612) | 53 (31/22) |  |
| Weiqiang Zhong[128] | 2013 | Guangdong (Zhaoqing) | 2012.9-2012.12 | Urban and Rural | | 15-88 (44.1±19.2) | 1212 (395/817) | 108 (51/57) | 8.91 (12.91/6.98) |
| Danchun Wang[129] | 2013 | Guangdong (Zhanjiang) | 2012.7 | Urban | | ≥40 | 0/137 | 0/7 | 0/5.11 |
| Yaoxian Tang[130] | 2010 | Guangdong (Lianshan) | 2008.6-2009.12 | Urban and Rural | | ≥20 | 32116(16880/15236) | 1103(663/440) | 3.43(3.92/2.89) |
| Jingdong Feng[131] | 2010 | Guangdong (Dongguan) |  | Urban | | >35 | 669(297/372) | 36(17/19) | 5.4(5.7/5.1) |
| Bin Chen[132] | 2011 | Guangdong (Zhuhai) | 2007.9-2007.12 |  | | 44 . 8 ±13 . 5 | 961 | 48(19/29) | 5(4.4/5.5) |
| Lizi Jin[133] | 2009.8 | Guangdong (Zhuhai) | 2007.6-12 | Rural | | 19.38±13.47 | 1134(427/710) | 87(35/50) | 7.7(8.2/7.1) |
| Jing Li[134] | 2009 | Guangdong (zhongshan) | 2008.5 | Urban | | >60 | 613(577/36) | 98 | 15.99 |
|  |  |  | 2004 | Urban | |  | 488(464/24) | 51 | 10.45 |
|  |  |  | 2005 | Urban | |  | 541(514/27) | 76 | 14.05 |
|  |  |  | 2006 | Urban | |  | 557(530/27) | 87 | 15.62 |
|  |  |  | 2007 | Urban | |  | 592(556/36) | 89 | 15.03 |
| Guang Zhao[135] | 2008 | Guangdong | 2007-2008 | Urban and Rural | | ≥20 | 31507(15807/15700) | 1127(648/479) | 3.58(4.10/3.05) |
| Biwen Xia[136] | 2005 | Guangdong (Zengcheng) | 2004-2005.12 | Urban/Rural | | ≥40 | 1531(726/805) | 136(67/69) | 8.9(9.2/8.6) |
| Hong Shao[137] | 2005 | Guangdong (Foshan) | 2002.5-2003.6 | Urban | | ≥60 | 3366(1449/1917) | 619 | 18.4(15.7/20.5) |
| Huiru Deng[138] | 2005 | Guangdong (Foshan) | 2002.9 | Rural | | 16-85(45.65±13.67) | 786(294/792) | 30(15/15) | 3.82(5.10/1.90) |
| Jinghua Chen[139] | 2004 | Guangdong (Foshan) | 1999-2002 | Urban | | 45.3±6.5(45.4±6.6/45.36±6.4) | 4083(1676/2407) | 194(70/124) | 4.75(4.20/5.30) |
| Shenren Chen[140] | 2004 | Guangdong (Shantou) | 1999-2001 | Urban and Rural | | ≥15 | 1662(777/884) | 75(27/48) | 4.51(3.47/5.43) |
| Bingying Pan[141] | 2005 | Guangdong (Guangzhou) | 2002.09-2002.12 | Urban | | ≥20 | 775(334/441) | 51(14/37) | 6.6(4.2/8.4) |
| Lin Sun[142] | 2005 | Guangdong (Shenzhen) | 2002 | Urban | |  | 4583(1656/2928) | 57 | 1.24 |
| Wenxing Liu[143] | 2000 | Guangdong (Guangzhou) | 1999.03-1999.08 | Urban | | 20-64 | 1601(1044/557) | 66(51/15) | 4.12(4.89/2.69) |
| Ji Peng[144] | 2001 | Guangdong (Shenzhen) | 1997 | Urban | | ≥ 20 | 8200(3120/5080) | 347(111/236) | 4.23(3.56/4.65) |
| Quanshui Han[145] | 2008 | Guangdong (Shenzhen) | 2005-2006 |  | |  |  |  |  |
|  |  |  | 2005 | Urban | | ≥20 | 2168(636/1532) | 52 | 2.39(3.77/1.82) |
|  |  |  | 2006 | Urban | | ≥20 | 2144(696/1448) | 54 | 2.52(4.16/1.72) |
| Diabetes Group[146] | 2001 | Guangdong | 1997~1998 | Urban and Rural | |  | 11742(5450/6292) | 414(200/214) | 3.5(3.7/3.4) |
| Limin Han[147] | 2002 | Guangdong (Shenzhen) | 2000 | Rural | | ≥25 | 5793(2191/3602) | 147(57/90) | 2.54(2.60/2.50) |
| Zunyong Liu[148] | 2000 | Guangdong (Shenzhen) | 1997 | Urban and Rural | | 20-75 | 8182(3943/4239) | 409 | 5 |
| Jinqing He[149] | 1999 | Guangdong (Gaozhou) | 1998.3 | Urban and Rural | | 20-74 | 606(274/332) | 14(6/8) | 2.31(2.19/2.41) |
| Lijun Li[150] | 2007 | Guangdong (Guangzhou) | 2006.06-2006.10 | Urban | | 60-97 | 390(162/228) | 49(22/27) | 12.6(5.6/6.9) |
| Yihua Yang[151] | 1998 | Guangdong (Shantou) | 1997.1 | Urban | | 36-76 | 150(130/20) | 15(14/1) | 10(10.8/5) |
| Shunzhen Wang[152] | 1988 | Guangdong (Guangzhou) | 1988 | Urban | |  | 3004(2418/586) | 82(57/25) | 2.7(2.3/4.2) |
| Tang Yan[153] | 1981 | Guangdong (Guanghzhou) | 1981 |  | |  | 42789 | 175 | 0.406 |
| Xiewen Tan[154] | 2008 | Guangdong (Guangzhou) | 2002.01-2005.12 | Urban/Rural | | 42±12/40±11 | 106260 |  |  |
|  |  |  | 2002 |  | |  | 23038 | 454(339/115) | 1.97（2.26/1.43） |
|  |  |  | 2003 |  | |  | 25589 | 601(436/165) | 2.34（2.68/1.77） |
|  |  |  | 2004 |  | |  | 28705 | 644(492/152) | 2.24（2.71/1.44） |
|  |  |  | 2005 |  | |  | 28928 | 944(714/230) | 3.26（3.92/2.15） |
| Shenglin Xia[155] | 2008 | Guangdong (Zhongshan) | 2000- | Urban | | 19-64 | 720 | 15 | 2.07 |
| Yuanzhen Chen[156] | 2013 | Guangxi (Binyang) | 2012.8-2012.11 | Rural | | ≥18 | 964 (435/529) | 83 (45/38) | 8.61 (10.34/7.18) |
| Xiaomin Deng[157] | 2013 | Guangxi (Nanning) | 2010.1-2011.12 | Urban | | 20-79 | 37318 (21101/16217) | 983 (663/320) | 2.63 (3.14/1.97) |
| Yongfang Xu[158] | 2007 | Guangxi(Nanning) | 2002 | Urban | | ≥18 | 412(183/229) | 20(7/13) | 4.85(3.82/5.68) |
| Zhiyuan Gan[159] | 2010 | Guangxi(Nanning) | 2009.3-2009.7 | Urban | | >60 | 953(457/496) | 127(69/58) | 13.33(15.1/11.69) |
| Yongfang Xu[160] | 2008 | Guangxi(Nanning) | 2000- | Urban and Rural | | 35-74 | 1413(663/750) | 111(66/45) | 7.86(9.96/6.00) |
| Jixin Lu[161] | 2012 | Guangxi (Guigang-Qintang) | 2012.3.8-16 | Rural | | 18-89 (51.3±16.7) | 1504 (782/722) | 147 (91/56) | 9.8 (11.6/7.8) |
| Hongxiang Li[162] | 2012 | Guangxi(Jing xi) | 2011.6 | Rural | | 20-92(52.8±14.2) | 561(277/284) | 57(31/26) | 10.2(11.2/9.2) |
| Defeng Wang[163] | 2010 | Guangxi(Zhanjiang) | 2010- | Urban | | ＞30 | 1000(438/562) | 81 | 8.1 |
| Qun Huang[164] | 2010 | Guangxi(Zhanjiang) | 2008.6 | Urban | | 30-82 | 584(340/244) | 24(12/12) | 4.11(3.53/4.92) |
| Xianglu Liu[165] | 2012 | Guangxi(Qingzhou) | 2010- | Urban | | ≥18 | 400(212/188) | 73(38/35) | 18.25(17.92/18.61) |
| Shumin Li[166] | 2010 | Guangxi(Qinzhou) | 2006-2007 | Urban | | 18-94 | 6156(3422/2734) | 626(340/286) | 10.17(9.94/10.46) |
| Shenghang Pang[167] | 2010 | Guangxi (Bobai) | 2008.3.3-2008.12.23 | Urban | | ≥18 | 1000(523/477) | 110(61/49) | 11.00(11.66/10.27) |
| Shaoping Chen[168] | 2010 | Guangxi(Wuzhou) | 2009 | Urban | | ≥49 | 1740(1032/714) | 97 | 5.5 |
| Mengwu Huang[169] | 2003 | Guangxi (Baise) | 2001.12-2002.10 | Urban | | ≥40 | 2012 | 168(87/81) | 8.35 |
| Anning Yao[170] | 2011 | Guangxi(Hezhou) | 2003-2005 | Urban and Rural | | ≥18 | 10692(5298/5394) | 267(138/129) | 2.5(2.6/2.4) |
| Zhenzhu Tang[171] | 2005 | Guangxi | 2002 | Urban and Rural | | ≥18 | 3060(1440/1620) | 66(32/34) | (2.2/2.1) |
| Shuzhen Zhou[172] | 2000 | Guangxi | 2000- | Urban | | ≥20 | 2854 (1835/1019) | 67 (44/24) | 2.35 (2.40/2.36) |
| Liangli Feng[173] | 1999 | Guangxi(Guilin) | 1995.6-8 | Urban | | 20-72 | 2089(1252/837) | 43(30/13) | 2.06 |
| Xiangzhen Lv[174] | 1995 | Guangxi(yulin) | 1990- | Rural | | ≥15 | 10616(5944/4672) | 164(91/73) | 1.545(1.53/1.56) |
| Diabetes Group[175] | 1983 | guangxi(wuming/xingan) | 1983 | Rural | | ≥20 |  |  |  |
|  |  | wuming |  |  | |  | 6878 | 78 | 1.13 |
|  |  | xingan |  |  | |  | 5564 | 97 | 1.74 |
| Zhenwang Fu[176] | 2014 | Hainan | 2014 | Rural | | 18-74 | 600 (291/309) | 50 | 8.3 |
| Chaoyong Zhu[177] | 2007 | Hainan | 2005/2006 | Urban | |  |  |  |  |
|  |  |  | 2005 |  | |  | 1251(825/426) | 60(43/17) | 4.80（5.21/3.99) |
|  |  |  | 2006 |  | |  | 1261(837/424) | 68(54/14) | 5.39(6.45/3.30) |
| Dongli Nie[178] | 2009 | Hainan | 2006 | Urban | | 20-86 | 2552(818/1734) | 125(67/58) | 4.5（6.7/5.8） |
| Shuxia An[179] | 2013 | Hebei (Zanhuang) | 2010- | Rural | | 30-70 | 3990 | 400 | 10.03 |
| Shuxia An[180] | 2014 | Hebei (Zanhuang) | 2010- | Rural | | 30-70 | 1626 (890/736) | 64 (31/33) | 3.94 |
| Fengge Chen[181] | 2014 | Hebei (Shijiazhuang) | 2010.11-2010.12 | Urban | | ≥18 | 908 (392/516) | 120 (55/65) | 13.22 (14.03/12.60) |
| Rongwei Ma[182] | 2007 | Hebei(Shijiazhuang) | 2004.4-2006.5 | Urban | |  | 5678(3957/1721) | 132(91/41) | 2.32(2.30/2.38) |
| Zigeng Guo[183] | 2014 | Hebei (Luancheng) | 2012.3-2012.5 | Rural | | ≥18 | 5379 | 518 | 9.63 |
| Yeqiang Liu[184] | 2014 | Hebei (Tangshan) | 2006 | Urban | | 53±13 | 96865 (77194/19671) | 9062(7457/1605) | 9.66/8.16 |
|  |  |  | 2008 |  | | <40 |  |  | 2.92/1.14 |
| Qiulian Zhang[185] | 2014 | Hebei (Tangshan) | 2012.6-2012.10 | Urban | | 45-93 (62) | 180040 (88220/91820) | 9182 | 5.83 |
| Baohua Wang[186] | 2013 | Hebei (Tangshan) | 2012.1-2012.12 | Urban | | (41.97±12.88) | 8443 (4471/3972) | 485 (318/167) | 5.74 (7.11/4.20) |
| Zhikun Zhang[187] | 2012 | Hebei (Tangshan) | 2009.1 | Urban and Rural | | 30-79 (48.9±11.7) | 252013 (131551/120462) | 7710 (3420/4337) | 3.1 (2.6/3.6) |
| Weihong Li[188] | 2012 | Hebei(Tangshan) | 2009-2011.5 | Urban and Rural | | ≥20 | 2423(1055/1368) | 149(57/92) | 6.15(5.40/6.73) |
| Qingwen Wu[189] | 2009 | Hebei(Tangshan) | 2000- | Urban | | ≥60 | 613(531/82) | 69 | 11.26 |
| Hong Zhang[190] | 2014 | Hebei | 2012.5-2012.10 | Urban | | ≥40 | 1363 (734/629) |  |  |
| Guosong Yang[191] | 2014 | Hebei (Yutian) | 2011.3 | Rural | | 40-69 | 495 (203/292) | 60 (25/35) | 12.1 (12.3/12.0) |
| Xiuyin Zheng[192] | 2010 | Hebei(Longhua) | 2008 | Urban | | ≥21 | 3447(2619/828) | 288(231/57) | 8.36(8.82/6.88) |
| Kaiyan Kang[193] | 2012 | Hebei(Qinhuangdao) | 2010.8-10 | Urban | | 20-92 | 3569(1778/1791) | 512(337/175) | 14.35(18.95/9.77) |
| Junqing Zhu[194] | 2007 | Hebei | 2004.9-2004.10 | Urban and Rural | |  | 4200(1734/2466) | 244 | 5.81 |
| Yurong Liu[195] | 2007 | Hebei(Baoding) | 2000- | Urban | |  | 643(388/255) | 193(89/104) | 30.0(22.9/40.8) |
| Jihong Li[196] | 2007 | Hebei(Zhangjiakou) | 2000- | Urban and Rural | | ≥40 | 2186(1139/1047) | 128(74/54) | 5.86(6.05/5.16) |
| Jianfeng Yang[197] | 2009 | Hebei(Handan) | 2008.01-09 | Urban | | 35-59 | 3244(2590/654) | 114 | 3.5 |
| Yongfeng Wang[198] | 2009 | Hebei | 2007 | Rural | | ≥30 | 5962(3802/2160) | 221(152/69) | 3.71（4.00/3.19) |
| Cuigai Zhang[199] | 2011 | Hebei | 2009.4-7 | Urban | | 25-84 | 3753(1855/1898) | 308(146/162) | 8.21(7.87/8.53) |
| Zhifu Wang[200] | 2004 | Hebei(Tanghai) | 2002.9.6-2002.10.24 | Urban and Rural | | 3-77 | 653(311/342) | 22(10/12) | 3.37(3.22/3.51) |
| Shengfen Wang[201] | 1999 | Hebei | 1996.10 | Urban | | ≥30 | 370(200/170) | 16(9/7) | 4.32(4.50/4.12) |
| Xia Jiang [202] | 1999 | Hebei | 1997 | Urban and Rural | | 20-74 | 2762 | 77 | 2.79 |
| Xin Wang[203] | 1994 | Hebei (tangshan) | 1990- |  | | ≥18 | 4123(2993/1130) | 53(41/12) | 1.29(1.37/1.06) |
| Lei Cao[204] | 2014 | Henan (Xiping) | 2010- | Urban and Rural | | ≥20 | 620 (283/337) | 52 (27/25) | 8.39 (9.54/7.42) |
| Yongqi Li[205] | 2014 | Henan | 2010- | Urban and Rural | |  | 6667 | 687 | 10.3 |
| Li Gao[206] | 2014 | Henan | 2010- | Urban and Rural | | 15-74 | 18772 (8421/10351) | 1729 (804/925) | 9.21 (9.54/8.94) |
| Qing Cai[207] | 2013 | Henan (Xinzheng) | 2010- | Urban and Rural | | ≥60 | 45294 (20880/24414) | 4312 (1783/2529) | 9.52 (8.54/10.36) |
| Chunting Sun[193] | 2012 | Henan (Zhengzhou) | 2011.3-2011.5 | Urban | | 55-89 (68.19±9.4) | 2153 (897/1256) | 179 (83/96) | 8.31 (9.25/7.64) |
| Yan Liu[208] | 2012 | Henan (Zheng zhou) | 2010.10-11 | Urban | | ≥20 | 600(246/354) | 75(34/41) | 12.50(13.82/11.58) |
| Yajuan Sun[209] | 2012 | Henan(Zhengzhou) | 2008 | Urban | | 45.89±12.05 | 3929(2036/1893) | 397 | 10.1 |
| Yuqing Hou[210] | 2012 | Henan (Xinxiang) | 2000-2010 | Urban | | 21-90 | 6798 | 378 | 5.56 |
|  |  |  | 2000 | Urban | |  | 1085 | 33 | 3.04 |
|  |  |  | 2004 | Urban | |  | 1117 | 42 | 3.76 |
|  |  |  | 2006 | Urban | |  | 1247 | 49 | 3.93 |
|  |  |  | 2008 | Urban | |  | 1544 | 84 | 5.44 |
|  |  |  | 2010 | Urban | |  | 1805 | 170 | 9.42 |
| Qinghong Li[211] | 2012 | Henan(Jiaozuo) | 2010-2011 | Urban and Rural | | 30-81 | 663(388/275) | 157 | 23.68 |
| Meili Wang[212] | 2012 | Henan(Jiaozuo) | 2011.1-2011.6 | Urban | | 20-60 | 800(500/300) | 142(37/75) | 21.2(7.4/25) |
| Yanqing Ma[213] | 2011 | Henan(Pingdingshan) | 2009 | Urban | | ≥19 | 1919 | 228 | 11.9 |
| Yiping Liu[214] | 2012 | Henan | 2011.3-2011.6 | Urban | | 21-84 | 2888(1655/1233) | 122(102/20) | 4.22(6.16/1.62) |
| Liming Yang[215] | 2010 | Henan(Xin'an) | 2007.7~2008.8 | Rural | | 35-74 | 16953(6615/10338) |  | 8.79(7.83/9.40) |
|  |  |  | 2000~2001 |  | |  | 7647(3976/3851) |  | 5.23(5.07/5.40) |
| Yuxia Cui[216] | 2011 | Henan(mianchi) | 2010.8-2010.10 |  | | ≥25 (48.5±10.6) | 1109(464/645) | 84(26/58) | 7.6(5.6/8.99) |
| Yanyan Li[217] | 2008 | Henan | 2005-2006 | Rural | | ≥18 (48.26±14.19) | 989(415/574) | 111 | 11.22 |
| Xiaofang Zhang[218] | 2008 | Henan(Luoyang) | 2008 |  | |  | 3015 | 117 | 3.88 |
| Xiuying Yang[219] | 2009 | Henan | 2006.4 | Urban | | ≥21 | 655(344/311) | 30 | 4.6 |
| Dongmei Xu[220] | 2011 | Henan | 2008.8/2009.3 | Rural | | ≥18 | 0/579 | 0/68 | 0/11.74 |
| Xicai Qiu[221] | 2003 | Henan(Jiaozuo) | 2001.10-2001.12 | Urban | | ≥15 | 4298(2089/2209) | 107(50/57) | 2.5(2.4/2.6) |
| Lei Zhang[222] | 2010 | Henan | 2002 | Urban and Rural | | ≥15 | 11236 | 618 | 5.5(5.3/5.6) |
| Weili jiang[223] | 2004 | Henan(Puyang) | 1992 | Urban | | 40-60 | 3476(2086/1390) | 70 | 2.01 |
| Qingfeng Tian[224] | 2002 | Henan | 2000- | Urban and Rural | | ≥20 | 5975 | 287 | 4.8 |
| Guohua Liu[225] | 2002 | Henan | 1997 | Urban and Rural | | 20~74 | 5975(2771/3204) | 287(117/170) | 4.80(4.22/5.31) |
| Tongxian Shao[226] | 2002 | Henan(Luoyang) | 2001.11 | Urban | | 35~84 | 244(156/88) | 21(14/7) | 8.61(8.97/7.95) |
| Jiming Liu[227] | 1998 | Henan(Anyang) | 1996.5-1996.7 | Urban | | 40-91 | 3056(1955/1101) | 154 | 5.04 |
| Rundai Feng[228] | 1992 | Henan(zhengzhou) | 1984-1991 | Urban | |  | 42408(27687/14711) | 1028(670/349) | 2.42(2.45/2/37) |
|  |  |  | 1984 | Urban | |  | 3393 | 36 | 1.06 |
|  |  |  | 1985 | Urban | |  | 4045 | 50 | 1.24 |
|  |  |  | 1986 | Urban | |  | 4224 | 74 | 1.75 |
|  |  |  | 1987 | Urban | |  | 5783 | 115 | 1.99 |
|  |  |  | 1988 | Urban | |  | 6622 | 154 | 2.33 |
|  |  |  | 1989 | Urban | |  | 6554 | 167 | 2.67 |
|  |  |  | 1990 | Urban | |  | 6570 | 223 | 3.39 |
|  |  |  | 1991 | Urban | |  | 5467 | 209 |  |
| Yifeng Lu[229] | 2008 | Henan(Zhengzhou) | 2005.08-2006.03 | Rural | | 18-88 (48.74±13.75) | 987(407/571) | 111(43/68) | 11.25（10.57/11.91） |
| Ling Xu[230] | 2013 | Henan/Jiangsu/Shǎnxi | 2010 | Urban and Rural | | ≥12 | 1535 (743/792) | 17 (6/11) | 1.3 (0.9/1.6) |
| Jian Wang[231] | 2012 | Heilongjiang(Haerbin) | 2008 | Urban | | 49.31±12.33 | 7913(5072/2841) | 1145(682/463) | (13.45/16.30) |
| Yan Feng[232] | 2012 | Heilongjiang  (Mudanjiang) | 2010- | Urban | | 20-79 | 3984 | 441 |  |
| Hong Sun[233] | 2005 | Helongjiang(Haerbin) | 2001.08 | Urban | | ≥20 | 2689(1252/1437) | 170(77/93) | 6.32(6.15/6.47) |
| Linyan Fu[234] | 2005 | Heilongjiang(Qiqihaer) | 2001.11-2003.03 | Urban | | 25-74 | 1468 | 61 | 4.16 |
| Xiuping Wu[235] | 1999 | Heilongjiang | 1990- | Urban and Rural | | ≥40 | 1484 | 85 | 5.73 |
| Jinping Wang[236] | 1999 | Heilongjiang(Daqing) | 1985/1997 | Urban | | 25-74 | 9832(4910/4922) |  |  |
|  |  |  | 1985 | Urban | | 25-74 | 9832(4910/4922) | 234(122/112) | 2.55(2.77/2.33) |
|  |  |  | 1997 | Urban | | 25-74 | 9832(4910/4922) | 293(160/133) | 3.22(3.65/2.79) |
| Diabetes Group[237] |  | Heilongjiang | 1980.3-6 |  | |  | 13295(7479/5816) | 150(84/66) | 1.13(1.12/1.13) |
| Xin Wei[238] | 1995 | Heilongjiang(haerbin&daqin) | 1995 |  | | 40-60 | 1706(906/800) | 101(54/47) | 5.92(5.96/5.87) |
| Guangyong Li[239] | 2008 | Heilongjiang(Daqing) | 2007.02-2007.03 | Urban | | ≥20 | 10158(5086/5072) | 518(262/256) | 5.1(5.15/5.05) |
| Xiaofei He[240] | 2011 | Hunan (Zhuzhou) | 2010- | Urban | | 20-90 | 1250 (650/600) | 150 (80/70) | 12.0 (12.3/11.7) |
| Xian Liu[241] | 2009 | Hunan (Nan county) | 2009 | Urban | | ≥30 (51.5±16.1) | 2160 (1151/1009) | 66 (39/27) | (3.39/2.68) |
| Ying Huang[242] | 2014 | Hunan (Changde) | 2010.1-2011.1 | Urban | | 40-87 | 682 (417/265) | 97 (65/33) | 14.22 (15.59/12.45) |
| Shisong Lian[243] | 2010 | Hunan(Ruanling) | 2008.3~2009.7 | Rural | |  | 26811(8225/18786) | 677(193/484) | 2.53(2.35/2.58) |
| Xian Liu[241] | 2009 | Hunan(Yiyang-Nanxian) | 2000- | Urban | | 30-84(51.5±16.1) | 2160(1151/1009) | 66(39/27) | 3.06(3.39/2.68) |
| Li Li[244] | 2010 | Hunan(Hengyang) | 2008.1-2008.12 | Urban | | ≥20 (44.12 ±12.96) | 5368(3890/1478) | 434(362/72) | 8.08(9.31/4.87) |
| Qingtao Su[245] | 2003 | Hunan(Luxi) | 2002 | Urban and Rural | | ≥35 | 698(525/173) | 14(10/4) | 2.01(1.9/2.3) |
| Jufang Dai[246] | 2004 | Hunan(Yongzhou) | 2000.4-5and2000.9-10 | Urban and Rural | | ≥60 | 9827 | 589 | 5.99 |
| Yonghong Sha[247] | 2008 | Hunan(Xiangxi/Changsha) | 2000- | Urban and Rural | | >20 | 4043 | 172 | 4.25 |
| Yiping Duan[248] | 1997 | Hunan(Changsha) | 1996 | Jishou | | 21-89 | 1369(986/383) | 63(54/9) | 4.6(5.5/2.3) |
| Jimeng Wang[249] | 1997 | Hunan(Hengyang) | 1995.1-1995.3 | Urban and Rural | | ≥25 | 2005(905/1100) | 78(22/22) | 2.19(2.43/2) |
| Hongli Du[250] | 2005 | Hubei (Wuchang) | 2002.8-2004.11 | Urban | | ≥40 | 4651 | 335 | 7.2 |
| Xu Liu[251] | 2014 | Hubei (Xiangyang) | 2010- | Urban | | ≥18 | 4945 (1993/2952) | 442 (128/314) | 8.94 (6.42/10.64) |
| Shaobing Zhang[252] | 2014 | Hubei (Lengji) | 2012.3-2013.8 | Urban | | ≥60 | 2196 (1061/1135) | 171 | 7.78 |
| Qiulin Wang[253] | 2014 | Hubei (Wuhan) | 2008 | Urban | | 20-93 (52.03±3.26) | 4103 (2116/1987) | 182 (103/79) | 4.44 (2.51/1.93) |
|  |  |  | 2010 |  | | 20-95 (52.63±2.56) | 4229 (2197/2302) | 302 (186/116) | 7.14 (4.40/2.74) |
| Yan Guo[254] | 2014 | Hubei (Wuhan) | 2012.5-2012.12 | Urban and Rural | | 65-111 (71.99±5.70) | 357684 (166312/191372) | 44887 (19118/25769) | 12.55 (11.50/13.47) |
| Zhenye Zhang[255] | 2014 | Hubei (Wuhan) | 2010 | Urban | | 20-93 (52.03±3.26) | 4103 (2116/1987) | 182 (103/79) | 4.44 (4.87/3.98) |
|  |  |  | 2012 |  | | 20-95 (52.63±2.56) | 4229 (2197/2032) | 302 (186/116) | 7.14 (8.79/5.04) |
| Jin Jin[256] | 2010 | Hubei(Wuhan) | 2008.10-2009.7 | Urban | | ≥18 | 8140(3848/4292) | 700 (373/326) | 8.6(9.7/7.6) |
| Yabin Jing[257] | 2010 | Hubei(Wuhan) | 2008-2009 | Urban | | ≥20 | 2183(1044/1139) | 109 | 4.99 |
| Maowei Cheng[258] | 2010 | Hubei | 2002 | Urban and Rural | | ≥18 | 2204(1037/1167) | 60(28/32) | 2.7(2.7/2.7) |
| Shaoping Zhang[259] | 2011 | Hubei(Hefeng) | 2008 | Urban | | ≥15 | 5219(3638/1581) | 200(167/35) | 3.87(4.59/2.21) |
| Chunjiao Wang[260] | 2012 | Hubei(Wuhan) | 2010- | Urban | | 35-75 | 2251(1113/1138) | 151(76/75) | 6.70(6.83/6.59) |
| Changfeng Li[261] | 2008 | Hubei(Wuhan) | 2006 | Urban and Rural | |  | 1879(750/1129) | 168 (75/93) | 8.94 (10/8.24) |
| Renxiang Liu[262] | 2007 | Hubei(Wuhan) | 2002 | Urban and Rural | | ≥15 | 356(170/186) | 10(5/5) | 2.81(2.94/2.69) |
| Xingju Luo[263] | 2012 | Hubei(Yichang) | 2008.2- | Rural | | 35-74 | 9871(4075/5796) | 441(182/259) | 4.47(4.47/4.47) |
| Xuewen Gao[264] | 2012 | Hubei (Yichang-Yiling) | 2007.9-2008.3 | Rural | | ≥35 | 9871 (4076/5795) | 441 (182/259) | (4.47/4.48) |
| Xiaofang Zhang[265] | 2007 | Hubei(Jingzhou) | 2000- | Rural | |  | 3156(1652/1504) | 169(76/93) | 5.4(4.6/6.2) |
| Yang Gao[266] | 2006 | Hubei(Xiao Gan) | 2005.4-6 | Urban | |  | 611(293/318) | 11(4/7) | 1.8(1.37/2.21) |
| Linshuang Zhao[267] | 2005 | Hubei(Wu Han) | 2002.1-12 | Urban | | ≥40 | 2600 | 303 | 11.7 |
| Wenfan Ma[268] | 2004 | Hubei(Jianghan) | 1994 / 2003.1 | Urban | | 20-70 |  |  |  |
|  |  |  | 1994 | Urban | |  | 989 | 55 | 5.56 |
|  |  |  | 2003 | Urban | |  | 1087 | 44 | 4.05 |
| Chaoxin Li[269] | 2002 | Hubei(Zaoyang) | 1994 | Urban | | 25-75 | 2326 | 49 | 2.106 |
|  |  |  | 1999 | Urban | | 25-75 | 2154 | 68 | 3.156 |
| Yizhen Yu[270] | 2002 | Hubei(Wuhan) | 1992 | Urban | |  | 2776 | 27 | 0.97 |
|  |  |  | 1994 |  | |  | 2772 | 39 | 1.41 |
|  |  |  | 1997 |  | |  | 2881 | 59 | 2.05 |
|  |  |  | 2000 |  | |  | 2999 | 89 | 2.97 |
| Jing Li[271] | 1999 | Hubei | 1994-1995 | Urban and Rural | | ≥25 | 9836 | 167 | 2.97 |
| Zengzhen Wang[272] | 1997 | Hubei | 1994.7-1995.3 | Urban and Rural | | ≥25 | 9450(4790/5660) | 248(137/111) | 2.62(2.86/2.38) |
| Martin C.S.[273] | 2013 | Hong Kong | 2001.1-2001.5,2002.5-2002.7,2005.11-2006.3,2008.2-2008.5 | Urban and Rural | | ≥15 |  |  |  |
|  |  |  | 2001 |  | |  | 33609(16484/17125) | 1019(462/557) | 3.03（2.80/3.25) |
|  |  |  | 2002 |  | |  | 29561(14663/14898) | 923(421/502) | 3.12 (2.87/3.37) |
|  |  |  | 2005 |  | |  | 29802(14728/15074) | 1057(489/568) | 3.55 (3.32/3.77) |
|  |  |  | 2008 |  | |  | 28923(14189/14734) | 1296(661/635) | 4.48 (4.66/4.31) |
| G T C Ko[274] | 2001 | Hong Kong | 2000- | Urban | |  | 3718(715/3003) | 786(242/544) | 21.1(33.8/18.1) |
| C.S. Cockram[275] | 1993 | Hong Kong | 1990- | Urban | | ≥18 (38.6±9.1/36.7±9.2) | 1513(910/603) | 70 | 4.5(5.1/3.6) |
| T.H.Lam[276] | 2000 | Hongkong | 1994.12-1996.10 | Urban | | 25-74 | 2664(1316/1348) | 253(124/129) | 9.50(9.40/9.60) |
| Gang Wang[277] | 2014 | Jilin (Changchun) | 2011.6-2011.12 | Urban | | ≥40 | 9236 (3041/6195) | 2495 | 27.0 (30.8/25.8) |
| Jingbo Zhang[278] | 2013 | Jilin | 2010.9.2010.12 | Urban and Rural | | ≥18 | 2993 (1425/1586) | 484 (246/238) | 16.17 (17.26/15.18) |
| Jinmei Zhao[279] | 2013 | Jilin (Changchun) | 2012.5-2012.7 | Urban | | 18-92 (47) | 700 (320/380) | 42 (19/23) | 6.0 (5.94/6.05) |
| Yan Yan[280] | 2012 | Jilin (Changchun) | 2011-2012 | Urban | | 20-69 | 27341 (20759/6582) | 1565 (1401/164) | 5.72 (6.75/2.49) |
| Yingli Zhu[281] | 2007 | Jilin | 2002.8-2002.12 | Urban and Rural | |  | 1600(785/815) | 50(24/26) | 3.1(3.0/3.2) |
| Yingli Zhu[282] | 2007 | Jilin | 2004.9-2004.11 | Urban and Rural | |  | 1260(554/706) | 22(10/12) | 1.75(1.8/1.7) |
| Yingli Zhu[283] | 2008 | Jilin(Baicheng+Jiutai) | 2002 | Urban and rual | |  | 859(407/454) | 46(20/26) | 5.4(4.9/5.7) |
| Chengwu Jiao[284] | 1998 | Jilin | 1996 | Urban and Rural | | ≥20 | 3568(1634/1934) | 134(54/80) | 3.76(3.3/4.14) |
| Jianqin Gu[285] | 2013 | Jiangsu (Jiangyin) | 2012 | Rural | | 30-90 | 9012 (3575/5437) | 293 (62/231) |  |
| Donglian Xu[286] | 2014 | Jiangsu (Nanjing) |  | Urban | | ≥20 (56.9±15.6) | 2986 (1830/1156) | 177 (124/53) | 5.9 (6.8/4.6) |
| Jing Wang[287] | 2014 | Jiangsu (Nanjing) | 2011 | Urban | | 40-75 (51±3) | 408 (153/255) | 77 | 18.87 |
| Qing Ge[288] | 2013 | Jiangsu (Nanjing) | 2012.3-2012.6 | Urban | | ≥18 | 8276 (3915/4361) | 476 (331/145) | 5.75 (8.45/3.32) |
| Kegui Yan[289] | 2013 | Jiangsu (Nanjing) | 2006.3-2006.10 | Urban | | 22-86 (45.2) | 482 | 33 | 6.85 |
| Xia Feng[290] | 2012 | Jiangsu(Nanjing) | 2006-2011 | Urban | |  | 37138 | 977 | 2.62 |
| Baichun Zhu[291] | 2012 | Jiangsu(Nanjing) | 2010.1 | Urban | | ≥18 | 812(353/459) | 110(53/57) | 13.6（15.0/12.4） |
| Jing Wang[292] | 2009 | Jiangsu(Nanjing) | 2007.7 | Urban | | 35-98(55±4.2) | 9093(4721/3472) | 973 | 10.7 |
| Min Ni[293] | 2007 | Jiangsu(Nanjing) | 2005 | Urban | | 37.18 | 1749(1110/639) | 17(15/2) | 0.97(1.35/0.31) |
| Qian Luo[294] | 2010 | Jiangsu(Nanjing) | 2007.6-2008.12 | Urban | | 18-82 | 2317(1447/870) | 103(80/23) | 4.4(5.5/2.6) |
| Yingying Chen[295] | 2013 | Jiangsu (Gaochun) | 2010.7-2010.10 | Rural | | ≥15 | 14608 | 175 (7332/7276) | 1.2 (0.9/1.5) |
| Fen Li[296] | 2014 | Jiangsu (Suzhou) | 2012.6-2012.8 | Rural | | 35-92 (56.49) | 2341 (1003/1338) | 252 (121/131) | 10.76 (12.06/9.79) |
| Jiansong Ding[297] | 2014 | Jiangsu (Suzhou) | 2010- | Urban | | 53.16±10.05 | 2727 (1056/1671) | 259 (122/137) | 9.50 (11.55/8.20) |
| Lingchi Wang[298] | 2012 | Jiangsu(Su Zhou) | 2010 | Urban/Rural | | ≥20 | 30578(12799/17779) | 2483(1027/1456) | 8.12 |
| Dandan Zhu[299] | 2014 | Jiangsu (Huaian) | 2013.3-2013.5 | Urban and Rural | | ≥18 | 6410 (3144/3266) | 389 (187/202) | 6.07 (5.95/6.18) |
| Changan Qiao[300] | 2014 | Jiangsu (Gaoyou) | 2012.12 | Rural | | ≥18 | 10308 (4772/5536) | 770 (294/476) | 7.47 (6.16/8.60) |
| Bo Cai[301] | 2013 | Jiangsu (Rugao) | 2010 | Rural | | 18-95 (55.16±14.55) | 600 (220/380) | 46 | 7.7 |
| Yongqing Zhang[302] | 2013 | Jiangsu | 2010 | Urban and Rural | | 18-95 (52.5±14.9) | 8400 (3805/4595) | 714 | 8.5 |
| Dandan Wang[303] | 2013 | Jiangsu (Xuzhou) | 2010- | Urban | | ≥15 | 2858 | 164 () | 5.74 (5.86/5.63) |
| Ziju Zheng[304] | 2009 | Jiangsu(Xuzhou) | 2000- | Urban | | ≥18 | 1028(469/559) | 291(135/156) | 28.31 |
| Xia Zhang[305] | 2008 | Jiangsu(Xuzhou) | 2007-2008 | Urban | |  | 1065(714/351) | 53(45/8) | 4.98(6.30/2.28) |
| Xiaoyong Gu[306] | 2013 | Jiangsu (Zhenjiang) | 2011.5-2011.8 | Urban and Rural | | ≥18 | 3953 (1776/2177) | 404 | 10.2 |
| Haisheng He[307] | 2012 | Jiangsu(Yuhang) | 2011.3-2011.5 | Urban | | ≥50 | 1166 | 123 | 10.55 |
| Xiaowei Zhu[308] | 2012.4 | Jiangsu (Wuxi) | 2010.06-12 | Urban | | ≥50 | 5316(2223/3093) | 656(275/381) | 12.37/12.32 |
| Ming Xu[309] | 2010 | Jiangsu(Wuxi) | 2010- | Urban | | ≥20 | 10810(4389/6421) | 1103(472/631) | 10.20(10.75/9.83) |
| Na Sun[310] | 2009 | Jiangsu(Wuxi) | 2008 | Urban | | ≥40 | 2101(807/1294) | 300(119/181) | 14.28(14.75/13.99) |
| Yaming Fan[311] | 2006 | Jiangsu (Changzhou) | 2004.4-2004.12 | Urban | | >25 | 8578(3708/4870) | 480(190/290) | 5.6(5.12/5.95) |
| Hongming Zhu[312] | 2008 | Jiangsu(Changzhou) | 2005 | Urban and Rural | | ≥20 | 20712 | 1183(477/706) | 5.71(5.43/5.92) |
| Jinde Lin[313] | 2006 | Jiangsu(Xu Zhou) | 2000 | Urban/Rural | | ≥35 | 41786(20622/21164) | 159(53/106) | 0.38(0.26/0.5) |
| Xiaoqun Pan[314] | 2006 | Jiangsu | 2002 | Urban/Rural | | ≥18 | 2935(1348/1587) | 108(47/61) | 3.68(3.49/3.84) |
| Jianping Huang[315] | 2006 | Jiangsu(Nan Tong) | 2005.6-8 | Urban/Rural | | ≥20 | 3849(1721/2128) | 236(110/126) | 6.13(6.37/5.94) |
| Xiaohua Liu[316] | 2011 | Jiangsu(Fengxian) | 2010- | Urban and Rural | | ≥60 | 6688(3052/3636) | 195(96/99) | 2.92(3.15/2.72) |
| Yinfen Zhang[317] | 2011 | Jiangsu(Taizhou) | 2010- | Urban and Rural | | ＞35 | 3442(1735/1707) | 66(35/31) | 1.92 |
| Weiju Tang[318] | 2011 | Jiangsu(Qidong) | 2010- | Urban and Rural | | ＞18 | 1082 | 85 | 7.86 |
| Ye Wu[319] | 2010 | Jiangsu(Changshu) | 2007-2008 | Urban and Rural | |  | 1708(757/949) | 90(40/50) | 5.27(5.2/5.2) |
| Fulin Ren[320] | 2005 | Jiangsu(Taicang) | 2000- | Urban | | ≥15 | 570 | 13 | 2.3 |
| Xia quan[321] | 2001 | Jiangsu(Haian) | 2000.4~2000.7 | Urban | | 35~99 (54) | 5835(2459/3376) | 34 (12/22) | 0.58(0.49/0.66) |
|  |  |  |  |  | |  |  |  |  |
| Huaifang Song[322] | 2001 | Jiangsu(Donghai) | 1996 | Rural | | ≥25 | 2598(1105/1493) | 52(25/27) | 2(2.26/1.81) |
| Jimu Yin[323] | 2002 | Jiangsu(Suzhou) | 2000 | Rural | | ≥35 | 2697(1116/1581) | 69(30/39) | 2.56(2.69/2.47) |
| Jiannan Wu[324] | 1999 | Jiangsu(Xishan) | 1996.11 | Urban and Rural | | ≥20 | 2083(899/1184) | 78(38/40) | 3.74(4.23/3.38) |
| Xiangsheng Li[325] | 1999 | Jiangsu(Wujin) | 1999 | Rural | | ≥25 | 1606(661/945) | 108(32/76) | 6.72(4.84/8.04) |
| Hongbing Shen[326] | 1999 | Jiangsu | 1997 | Urban and Rural | | ＞40 | 5751 | 440 | 7.65 |
| Meiqi Lu[327] | 1999 | Jiangsu(Xuzhou) | 1994.10-1995.1 | Urban | | ≥25 | 5000(3077/1923) | 133(95/38) | 2.66 |
| Wanshuang Hang[328] | 2008 | Jiangsu | 2002 | Urban/Rural | | ≥20 | 2890(1326/1564) | 107 (46/61) | 3.7(3.5/3.9) |
| Jianshu Li[329] | 1996 | Jiangsu(Xishan) | 1994 |  | | ≥20 | 17807(8598/9209) | 97(34/63) | 0.411(0.292/0.526) |
| Yanhua Tao[330] | 2014 | Jiangxi (Nanchang) | 2012 | Urban | | 35-70 | 1406 (392/1014) | 73(29/44) | 5.19 (7.40/4.34) |
| Chenxiu Wang[331] | 2014 | Jiangxi (Nanchang) | 2011.6-2011.10 | Urban | | 40-90 (54.38±9.95) | 10008 (4007/6001) | 1348 (617/731) | 13.47 (15.40/12.18) |
| Ping Tu[332] | 2009 | Jiangxi(nanchang) | 2006.10-2007.9 | Urban | | ≥20 | 1494(603/891) | 174(94/80) | 11.6(15.9/9.0) |
| Qi Wu[333] | 2014 | Jiangxi (Jiujiang) | 2013.1-2013.6 | Urban | | 62-81 (68.3±9.3) | 1507 (786/721) | 248 (128/120) | 16.46 (16.28/16.64) |
| Jie Liu[334] | 2011 | Jiangxi | 2010- | Urban and Rural | | ≥18 | 3000(1435/1565) | 229(119/110) | 7.63(8.15/7.16) |
| Xuewen Liu[335] | 2010 | Jiangxi(Jiujiang) | 2004.4-2004.12 | Urban and Rural | | ≥25 | 8578(3708/4870) | 480(190/290) | 5.60(5.12/5.95) |
| Guolian Zhong[336] | 2008 | Jiangxi(Ganzhou) | 2000- | Urban | | ≥20(45.6) | 2908(1562/1346) | 138 | 4.75 |
| Yuhua Wang[337] | 2010 | Liaoning(Dalian) | 2006.5-2006.8 | Urban and Rural | | 20-74 | 6304(3064/3240) | 515(219/296) | 8.17(7.15/9.14) |
| Safeng Zou[338] | 2010 | Liaoning(Dalian) | 2004 | Urban | | 30-90 | 13691(6651/6972) | 2316 | 16.9 |
| Fang Xu[339] | 2008 | Liaoning(Dalian) | 2006 | Urban and Rural | | 20-74 | 6304 | 515 | 8.17 |
| Yexin Han[340] | 2008 | Liaoning(Dalian) | 2006 | Urban and Rural | | ≥20 | 602(273/329) | 40(16/24) | 6.64(5.86/7.29) |
| Zhaoqing Sun[341] | 2009 | Liaoning(Fu xin) | 2004.10-2006.6 | Rural | | >35(57.77±11.37/25.05±10.88) | 6412(2805/3607) | 641 (273/368) | 10.0(9.7/10.2) |
| Guangwei Li[342] | 1996 | Liaoning(Daqing) | 1986 | Urban and Rural | | 25-74 | 110660(55391/55269) | 820(426/394) | 0.74(0.77/0.71) |
| Yongmei Zhang[343] | 2009 | Liaoning(Zhuanghe) | 2006 | Rural | | ≥40 | 105 | 19 | 18.1 |
| Zhengnan Gao[344] | 2003 | Liaoning(Dalian) | 1997 | Urban and Rural | |  | 2497(1156/1323) | 220 |  |
| Guanghui Dong[345] | 2004 | Liaoning(Dandong) | 1999 | Urban | |  | 5622(2746/2876) | 185(7/108) | 3.29(2.80/3.76) |
| Xiaoguang LI[346] | 2005 | Liaoning(Shenyang) | 2002.4-2002.10 | Urban | | ≥20 | 1439 | 68 | 4.73 |
| Wangzhong[347] | 1999 | Liaoning (Shenyang) | 1999 | Urban | | 25-60 | 736 | 4 | 0.5 |
| Tiejun Liu[348] | 2006 | Liaoning(Dalian) | 2003-2006 | Urban | | 21-60 | 6748(5112/1636) | 533(452/81) | 7.90(8.84/4.95) |
| Shan Jiang[349] | 1998 | Liaoning(Shenyang) | 1996.11-1997.3 | Urban | | 45-75 | 2342(1542/800) | 57 | 2.43 |
| Li Hao[350] | 1996 | Liaoning(Dalian) | 1994.10-1994.11 | Urban and Rural | | ≥25 | 3195(1624/1571) | 55(32/23) | 1.72(2/1.5) |
| Jinxia Gao[351] | 2014 | Mongolian (Hohhot) | 2012 | Urban | | ≥18 | 2791 (1107/1684) | 84 (33/51) | 3.0 (3.0/3.0) |
| Mingzhu Bao[352] | 2014 | Mongolia (Xilinhot City) | 2012.9-2012.12 | Urban | | 18-90 (49.20±14.057) | 2411 (997/1414) | 118 (49/69) | 4.9 (4.81/4.87) |
| Ruiling Wang[353] | 2012 | Mongolian (Hohhot) | 2011- | Urban | | 18-96 | 46407(22932/23475) | 2275(1147/1128) | 4.90（5.00/4.80） |
| Rong Su[354] | 2008 | Mongolian (Hulunbeier) | 2006-2007 | Urban and Rural | | ≥18 | 434 | 16 | 3.68 |
| Fengqi Wang[355] | 1999 | Mongolian | 1996.10-1997.02 | Urban | | 20-74 | 4858(2087/2771) | 87(32/55) | 1.79(1.53/1.98) |
| Fengrong Gao[356] | 2006 | Mongolian (Hohhot) | 2000- | Urban | | 45-89(60.5 ±8.44) | 854(629/225) | 98 | 11.48 |
| Diabetes Group[357] | 1981 | Mongolian | 1980.5-8 |  | | ≥20 | 6900(3649/3251) | 22(12/10) | 0.3188(0.328/0.307) |
| Yao Wang[358] | 2014 | Ningxia | 2013 | Urban | | 22-60 | 1638 (1283/355) | 127 | 7.75 |
| Huiyun Fan[359] | 2014 | Ningxia (Yinchuan) |  | Urban | | 46.8±15.1 | 2437 (1124/1313) | 105 (52/53) | 4.31 (4.63/4.04) |
| Rongfang Ma[360] | 2013 | Ningxia (Lingwu) | 2012.6-2012.8 | Urban and Rural | | ≥40 |  |  |  |
| Jia Wen[361] | 2013 | Ningxia (Yinchuan) | 2012 | Urban | | 40.86±11.32 | 1510 (832/678) | 75 | 4.97 |
| Zhenggui Yang[362] | 2009 | Ningxia(yinchuan) | 2008.05-11 | Urban and Rural | | ≥20 | 1790 | 89 | 5.0(5.2/4.8) |
| Caijun Yin[363] | 2010 | Ningxia | 2007 | Urban | | 42.4±12.6 | 1980(1213/767) | 108(83/25) | 5.5(6.8/3.3) |
| Yanhua Ning[364] | 2012 | Ningxia(Yinchuan) | 2009.6-2009.8 | Urban | | ≥60 | 898(348/550) | 208(79/129) | 23.16(22.70/23.45) |
| Xian Chen[365] | 2012 | Ningxia (Shapotou) |  | Rural | | 15-39 |  |  |  |
|  |  |  | 2004 | Rural | | 40-59 | 419(180/240) | 0 | 0 |
|  |  |  | 2007 | Rural | | 15-39 | 321(154/167) | 0 | 0 |
|  |  |  | 2010 | Rural | | 15-39 | 600(295/305) | 14 | 2.3 |
| Yang Yang[366] | 2011 | Ningxia | 2009.04-2009.10 | Urban | | ≥20 | 3191(1905/1286) | 187(146/41) | 5.86(7.66/3.19) |
| Hui Ma[367] | 2008 | Ningxia(lingwu) | 2000- | Urban/Rural | | ≥18 | 2575(1124/1451) | 83 | 3.18 |
| Yujun Li[368] | 2013 | Qinghai (Huangzhong) | 2012.5 | Urban and Rural | | 15-69 | 500 | 17 | 3.4 |
| Wenhui Wei[369] | 2013 | Qinghai | 2009.6-2010.5 | Urban and Rural | | ≥18 | 1574 | 68 | 4.29 (4.37/4.23) |
| Huili Zhang[370] | 2009 | Qinghai | 2000- | Urban and Rural | | 42.47±13.48 | 4864(2905/1959) | 233 | 4.8 |
| Juhua Cui[371] | 2003 | Qinghai | 2002.1-2002.9 | Urban | | ≥15 | 2384(1185/1199) | 69(33/36) | 2.89(2.78/3.0) |
| Youzhen Wang[372] | 1982 | Qinghai(xining) | 1980- | Urban | |  | 1617 | 28 | 1.732 |
| Youzhen Wang[373] | 1981 | Qinghai(huangyuan/gonghe) | 1980- |  | |  | 10234(5805/4429) | 56(39/17) | 0.547(0.672/0.384) |
| Yun Qiao[374] | 2011 | Shanghai | 2009.2-2009.7 | Urban | | >45 | 600 (204/396) | 63 (25/38) | 10.5 (12.25/9.60) |
| Yiting Zhang[375] | 2014 | Shanghai (Dachang) | 2011.10-2012.9 | Urban | | 40-94 (59.2±12.4) | 694 (344/350) | 64 (23/41) | 9.2 |
| Yaping Gu[376] | 2014 | Shanghai | 2013.6-2013.7 | Urban | | ≥60 | 308 (207/101) | 58 | 18.83 |
| Raquel Villegas[377] | 2011 | Shanghai | 2002-2006 | Urban | | 40-74 | 3978 | 243 | 6.1 |
| Zhengchang Xia[378] | 2012 | Shanghai | 2006.3-12 | Urban | | 20-90  (56.49±16.75) | 9555(4646/4909) | 765(315/450) | 8.01(6.78/9.17) |
| Fang Xiang[379] | 2012 | Shanghai | 2009 | Urban | | 35-74  (53.34±9.95) | 1145(512/633) | 139(79/60) | 12.1(15.4/9..5) |
| Lei Tian[380] | 2010 | Shanghai | 2007-2008 | Urban | | ≥40 | 4752 | 219 | 4.6 |
| Xianfeng Xu[381] | 2012 | Shanghai | 2009.7-8 | Rural | | 20-74 | 1976(838/1138) | 194(70/124) | 9.82(8.35/10.90) |
| Jiong Xu[382] | 2011 | Shanghai | 2008.5-2008.8 | Urban | | >60 | 4566(2155/2411) | 1150 | 25.30 |
| Jinhui Tang[383] | 2011 | Shanghai | 2010.10-2010.11 | Rural | | ≥60 | 3620(1522/2098) | 3620(162/295) | 12.6(10.6/14.1) |
| Bo Cao[384] | 2011 | Shanghai | 2010.6-2010.7 | Rural | | ≥20 | 584 | 37 | 6.3 |
| Yinfei Zhang[385] | 2011 | Shanghai | 2010.3~2010.7 | Urban | | 40~83 | 2447(1021/1426) | 387 | 15.8 |
| Rui Li[386] | 2012 | Shanghai | 2002-2003 |  | | ≥35 | 12329 | 1442 | 11.7 (13.63/10.3) |
|  |  |  | 2009 |  | |  | 7423 | 1158 | 15.6 (17.4/14.1) |
| Jianfeng Chen[387] | 2011 | shanghai | 2010.3-2010.5 | Urban | | 70.0±8.2 | 3127(1376/1751) | 378 | 12.1 |
| Ruifang Xu[388] | 2011 | shanghai | 2010- | Urban | |  | 1000(480/520) | 91(32/59) | 9.1 |
| Guoliang Jiang[389] | 2008 | Shanghai | 2006 | Rural | | ≥60 | 1143(341/802) | 373(119/254) | 8.39(2.68/5.71) |
| Meiying Zhu[390] | 2008 | Shanghai | 2007 | Urban | | 18-69(46.23±11.97) | 1984(928/1056) | 109(49/60) | 5.5(5.3/5.7) |
| Liangfeng Wang[391] | 2008 | Shanghai | 2007 | Urban | | 15-69 | 904(435/469) | 56(24/32) | 5.8(5.2/6.4) |
| Jun Tayama[392] | 2014 | Shanghai | 2010.10-2011.11 | Urban | |  | 2228(1418/810) | 240(208/32) | 10.77(14.67/3.95) |
| Huili Xing[393] | 2007 | Shanghai(Baoshan) |  | Urban | | ≥40 | 2997 | 260 | 8.7 |
| Jianfeng Chen[394] | 2012 | Shanghai(Chuanshatangzhen) | 2009.1-2009.6 | Urban | | ≥40 | 554(104/450) | 71(18/53) | 12.8(17.3/11.8) |
| Chunxiang Wu[395] | 2009 | Shanghai | 2007 | Urban | | ≥15 | 1281 | 88 | 6.87 |
| Xu Li[396] | 2006 | Shanghai | 2002 | Urban | | ≥40(60.6±10.8) | 1716(534/1182) | 229 | 13.3 |
| Rui Li[397] | 2006 | Shanghai | 2003 | Urban/Rural | | ≥15 | 11589(4621/6968) | 1000(412/588) | 8.6（8.9/8.4） |
| Xiya Huang[398] | 2011 | Shanghai | 2010- | Urban | | ≥18 | 994(339/655) | 111 | 11.2 |
| Xinwen Zhang[399] | 2004 | Shanghai | 2002 | Urban | | ≥20 | 3429(1440/1989) | 257(120/137) | 7.49(8.31/7.04) |
| Ming Mi[400] | 2005 | Shanghai | 2002.8-2002.10 | Urban | | ≥18 | 1222(575/647) | 99(45/54) | 8.1(7.8/8.3) |
| Jie Yang[401] | 2005 | Shanghai | 2003 .9～ 2009.10 | Urban | | 50-70 | 2249 | 365 | 16.23 |
| Zaixia Xu[402] | 1999 | Shanghai | 1997 | Urban | | 25-75 | 10000(4642/5358) | 455(132/323) | 4.55（2.84/6.03) |
| Zhengyan Sheng[403] | 2001 | Shanghai | 1998 | Urban | | 30-79 (47.10±9.01) | 9376(4096/4307) | 299 | 3.19 |
| Yunxing Shi[404] | 1999 | Shanghai | 1991.1 | Urban | | 63-90 | 404(404/0) | 76 | 18.8 |
| Xuelin Bai[405] | 2006 | Shanghai(Changning) | 2004 | Urban | | 20-98(51.24) | 2618(1183/1435) | 309(141/168) | 11.8(11.9/11.7) |
| Mingyao Zhao[406] | 2008 | Shanghai | 2005 | Urban | | 50-69 | 3192(1352/1840) | 264 | 8.27 |
| Zechun Jiang[407] | 2014 | Shandong (Rushan) | 2010 | Rural | | ≥30 | 2993 | 170 (72/98) | 5.68 (4.79/6.58) |
| Yan Wang[408] | 2012 | Shandong(Ru Shan) | 2009 | Rural | | >50 | 934(472/462) | 59(20/39) | 6.32(4.24/8.44) |
| Lihui Yang[409] | 2014 | Shandong (Laizhou) | 2010- | Urban | |  | 1000 (500/500) | 91 (49/42) | 9.1 (9.8/8.4) |
| Weiliang Liu[410] | 2014 | Shandong (Jinan) | 2010.9-2010.11 | Urban | | 21-85 (40.09±8.85) | 3444 (1451/1993) | 122 (82/40) | 3.54 (5.62/2.01) |
| Yajie Lin[411] | 2014 | Shandong (Jinan) | 2009-2013 | Urban | | ≥65 | 30351 | 4765 | 15.7 |
| Qing Xin[412] | 2013 | Shandong (Jinan) | 2012.2-2012.9 | Urban | | 60-93 (68.78±5.76) | 1123 (732/391) | 218 (143/75) | 19.4 (19.5/19.2) |
| Yu Lu[413] | 2010 | Shandong(Jinan) | 2008.1.1-2009.12.31 | Urban | | 41-93(64.3±12.6) | 1890(1612/278) | 188 | 9.95 |
| Pishan He[414] | 2012 | Shandong(Jinan) | 2010 | Rural | | ≥18 | 435(214/221) | 12 | 2.76 |
| Mingrui Tao[415] | 2009 | Shandong(Jinan) | 2006.3-2007.7 | Rural | |  | 50039(40869/9170) | 5080(4237/834) | 10.15(10.37/9.19) |
| Xiuling Zheng[416] | 2009 | Shandong(Jinan) | 2005 | Urban | | ≥45 | 4118(3205/913) | 619 | 15.03(15.32/14.02) |
| Shunping Li[417] | 2011 | Shandong(Jinan) | 2008.1-12 | Urban | | ≥20 (41. 40 ±9. 01) | 12094(9982/2112) | 714(652/62) | 5.90(6.53/2.80) |
| Jian Xu[418] | 2005 | Shandong(Jinan) | 2003 .5 -2003. 7 | Urban | | 50-89 | 355(335/20) | 35 | 9.9 |
| Jinxiang Li[419] | 2013 | Shandong (Zibo) | 2012.5,9,12 | Urban | | 60-97 (72.43) | 467 | 111 | 23.7 |
| Chengde Xue[420] | 2006 | Shandong(Zi Bo) | 2003-2005 | Urban | | ≥20 | 27621(18664/8957) | 971(652/319) | 3.52(3.5/3.56) |
| Dong Y.[421] | 2005 | Shandong(Qing Dao) | 2001-2002 | Urban/Rural | | 20-74 | 12240(5347/6893) | 747 | 6.1 |
| Jijun Liu[422] | 2013 | Shandong (Qingdao) | 2010 | Urban | | 20-91 (47±12.1) | 9020 (3676/5344) | 552 (238/314) | 6.12 (6.47/5.88) |
| F. Ning[423] | 2009 | Shandong(Qindao) | 2001-2002 | Urban/Rual | | 35-74 | 9445(3898/5547) | 247 |  |
|  |  |  | 2006 | Urban/Rual | |  | 5253(2019/3234) | 220 |  |
| Xianghai Zhou[424] | 2011 | Shandong (Qingdao) | 2006-2011 | Urban | | 35~74 | 2386(947/1439) | 637(270/367) | 26.7(28.5/25.5) |
| Yaoming Zhai[425] | 2007 | Shandong(Qingdao) | 2002 | Urban and Rural | | ≥18 | 3480 | 246 | 7.1 |
| Yuchun Wang[426] | 2010 | Shandong(Qingdao) | 2009.1-2009.12 | Urban | | 22-82 (51.17±13.76) | 8090(3350/4740) | 615 | 7.6(6.0/9.2) |
| Yan Li[427] | 2009 | Shandong(Qingdao) | 2006.4-6 | Rural | | ≥35 | 1722(742/980) | 268(122/146) | 15.76(16.44/14.89) |
| Yanhu Dong[428] | 2004 | Shandong(Qingdao) | 2002.1-2002.2 | Urban and Rural | | 20~74 | 12240(6893/5347) | 906(441/465) | 7.4(6.4/8.1) |
| Jingbo Tian[429] | 1999 | Shandong(Qingdao) | 1997.04-1997.11 | Urban | | >45 | 1170(761/409) | 70(43/27) | 5.98(5.65/6.60) |
| Chenli Zhu[430] | 2013 | Shandong (Weifang) | 2010- | Urban | | ≥30 | 1753 (643/1110) | 369 (154/215) | 21.05 (23.95/19.37) |
| Zhen Liu[431] | 2013 | Shandong (Jimo) | 2012.6.5-2012.9.20 | Urban and Rural | | 20-90 | 1194 | 81 | 6.87 |
| Chun Liu[432] | 2012 | Shandong (Ningyang) | 2010- | Urban | | 18-79 (44.24±13.28) | 310125 (138375/171750) | 15375 (8063/7313) | 4.96 (5.83/4.26) |
| Xi Chen[433] | 2012 | Shandong | 2011 | Urban and Rural | | 18-69(41.4±14.1) | 15350(7683/7667) | 794(428/366) | 4.59(4.98/4.19) |
| Xia Tang[434] | 2012 | Shandong(Yi Yuan) | 2011.6 | Rural | | ≥25 | 3124(1748/1376) | 69(30/39) | 2.21(1.72/2.83) |
| Aiqin Xue [435] | 2010 | Shandong | 2008 | Urban | | ≥45 | 420 (349/71) | 63 (57/6) | 15(16.3/8.5) |
|  |  |  | 2009 |  | |  | 436 (361/75) | 56 (51/5) | 12.8(14.1/6.7) |
| Chuanmao Zhong[436] | 2011 | Shandong(Binzhou) | 2009.03-2010.03 | Urban | | ≥16 | 9230(4283/4947) | 745 | 8.07 |
| Yongtang Wei[437] | 2011 | Shandong(Yantai) | 2008-2009 | Urban | | 39.70±11.97 | 3740(2286/1454) |  | 5.78(6.26/5.02) |
| Qingshun Hao[438] | 2007 | Shandong(Zaozhuang) | 2003.12-2007 |  | |  | 3952(1977/1975) | 249 | 6.3 |
| Tianji Liu[439] | 2009 | Shandong(Cangshan) | 2007 | Urban | |  | 1979(843/1136) | 57(22/35) | 2.88(2.61/3.08) |
| Jinming Chen[440] | 2008 | Shandong(Lijin) | 2007 | Rural | |  | 15210(7130/8080) | 110(46/67) | 0.72(0.65/0.83) |
| Zuomin Zhang[441] | 2008 | Shandong(Dongying) | 2003 | Urban | | ≥20 | 30034 | 1631 | 5.43 |
| Hui Li[442] | 2008 | Shandong | 2007 | Rural | |  | 20087(9127/10960) | 864(374/482) | 4.3(4.1/4.4) |
| Hongbo Yu[443] | 2007 | Shandong(Pingdu) |  | Rural | |  | 4298(1847/2451) | 258(96/162) | 6.00(5.20/6.61) |
| Xianyan Jiang[444] | 2007 | Shandong(Qingdaogang) | 1999.6-1999.10 | Urban | | ≥18 | 9004 | 153 | 1.7 |
| Yingchun[445] Tang | 2010 | Shandong(Liaocheng) | 2008.10-2009.10 | Urban | | ＞15 | 1818(1009/809) | 72 | 3.96 |
| Aixia Zhu[446] | 2006 | Shandong(Zao Zhuang) | 2004.5-6 | Urban/Rural | |  | 4828(2470/2368) | 23(16/7) | 0.48(0.65/0.3) |
| Shijie Sun[447] | 2011 | Shandong(Dezhou) | 2008.8-12 | Urban | | ≥20 | 4015(1927/2088) | 226 | 5.63 |
| Hui Yan[448] | 1995 | Shandong(Jinan) | 1993.3-1993.5) | Urban | | 60-81 | 1056 | 49 | 4.6 |
| Fenghuan Zhang[449] | 2008 | Shandong(Jinan) | 2003.01.01-2005.12.31 | Urban | | ≥18 | 49825(31166/18525/134) | 1566(1115/450) | 3.14（3.59/2.43） |
| Yali Guo[450] | 2014 | Shānxi (Xinjiang) | 2013.8-2013.10 | Urban and Rural | |  | 3400 (1614/1786) | 123 (38/85) | 3.62 (2.35/4.76) |
| Weidong Li[451] | 2014 | Shānxi (Shouyang) | 2010-2012 | Urban and Rural | | ≥18 | 184337 (94699/89638) | 16056 (8722/7334) | 8.71 (9.21/8.18) |
| Xiumin Shen[452] | 2011 | Shānxi (Taiyuan) | 2008.6-2008.11 | Urban | | ≥30 | 4895(1497/3398) | 604(230/374) | 12.34(15.36/11.01) |
| Yuping Tang[453] | 2009 | Shānxi(Taiyuan) | 2008.6-10 | Urban | | 35-75 | 4105(1292/2813) | 693(251/442) | 16.88(6.11/10.77) |
| Weiping Ma[454] | 2011 | Shānxi(Changzhi) | 2009-2010 | Urban and Rural | | ≥40 | 8493(4249/4244) | 606(298/308) | 7.14(7.03/7.26) |
| Xuewu Xing[455] | 2009 | Shānxi(Yuncheng) | 2005.7 | Rural | | 45.88±15.45 | 4381(1977/2404) | 131(65/66) | 3.0(3.3/2.7) |
| Shufen Zhao[456] | 2009 | Shānxi | 2005.8/2006.8 | Urban and Rural | | 47.84± 15.90 | 12111(5324/6787) | 638(309/329) | 5.27(5.80/4.85) |
| Jing Yang[457] | 1998 | Shānxi | 1994.8 | Urban and Rural | | ≥25 | 11550(5796/5754) | 240.07(152.82/86.86) | 2.08(2.64/1.51) |
| Peiwu Li[458] | 1995 | shānxi(taiyuan) | 1990- | Urban | | ≥25 | 1818(660/1158) | 46(22/24) | 2.53(3.333/2.073) |
| Diabetes Group[459] | 1981 | Shānxi | 1980.3-6 | Rural | |  | 32904(15975/16929) | 101(38/63) | 0.307(0.2379/0.3721 |
| Hongmei Lei[460] | 2010 | Shǎnxi(Xi'an) | 2009.10-2009.12 | Urban | |  | 1836(508/1328) | 291(68/223) | 15.85(13.4/16.8) |
| Chunmei Liu[461] | 2006 | Shǎnxi(Xianyang) | 2000- | Urban | | ≥40 | 5490 | 432 | 7.9 |
| Yanling Feng[462] | 2007 | Shǎnxi(Jinzhong) | 2003-2004 | Urban and Rural | | ≥18 | 2797(1710/1087) | 122 | 4.36 |
| Cailing Yang[463] | 2011 | Shǎnxi(Meixian) | 2010.1 |  | | ≥18 | 600(272/328) | 29(13/16) | 4.83(4.78/4.88) |
| Yaling Liu[464] | 2002 | Shǎnxi(xian) | 2000.10～2001.4 | Urban | | 30-60 | 1701(1114/587) | 63 | 3.7 |
| Xiaoli Jia[465] | 2011 | Sichuan (Wenchuan) | 2009.1-2009.4 | Urban | | 20-88 (58) | 3230 (1144/2086) | 326 | 10.09 (biao4.63) (13.46-6.30/8.25-3.75) |
| Hong Li[466] | 2010 | Sichuan(Beichuan) | 2009.3-2009.5 | Urban and Rural | | 15-69 | 601(211/390) | 34(11/24) | 5.6（5.21/5.89) |
| Changhua Xiao[467] | 2014 | Sichuan (Guanghan) | 2014.3-2014.5 | Urban | | ≥18 | 3835 (1929/1906) | 364 (168/196) | 9.49 (6.15/10.28) |
| Haitao Zhang[468] | 2014 | Sichuan (Ganzi) | 2010.9-2012.7 | Urban | | 18-90 (41.24±15.41) | 4898 | 471 | 9.62 (10.6/8.7) |
| Kaichao Yang[469] | 2013 | Sichuan (Luzhou) | 2010- | Urban | | 40-80 (59.33±12.28) | 500 | 3 |  |
| Xiaozhou Li[470] | 2012 | Sichuan(Luzhou) | 2011.4-2011.11 | Urban | | ≥40 | 10012 | 1122(1839) | 16.9 |
| Li Wei[471] | 2012 | Sichuan (Chengdu-Wuhou) | 2010.10-2011.6 | Urban | | ≥18 | 24675 (11722/12953) | 1202 (520/682) | 4.9 (4.4/5.3) |
| Xianhua Xu[472] | 2012 | Si Chuan(Cheng Du) | 2006 and 2010 | Urban and Rural | | 45-98 |  |  |  |
|  |  |  | 2006 |  | | 45-98 | 2669 | 374 | 14.0 |
|  |  |  | 2010 |  | | 45-98 | 2529 | 534 | 21.1 |
| Longxin Li[473] | 2012 | Sichuan(Chengdu) | 2007.05 | Urban | | 50-80(64.22±6.11/61.87±6.67) | 685(394/291) | 113(72/41) | 18.3/14.1 |
| Rulian Wang[474] | 2003 | Sichuan(Chengdu) | 1999- | Urban | | 18-80(46.87±14.15) | 7288 | 225 | 3.09 |
| Rong Hu[475] | 2006 | Sichuan(Chengdu) | 2004.1 | Urban | | 20-74 | 1445(598/847) | 66(26/40) | 4.57(4.35/4.72) |
| Xiance Luo[476] | 2011 | Sichuan(Chengdu) | 2008.4-11 | Urban and Rural | | 60-79(67. 01±5. 6) | 1951(880/1071) | 532(244/288) | 27.27(27.73/26.89) |
| Xingrong Ye[477] | 2004 | Sichuan(Chengdu) | 2000- | Urban | | 60~96 | 1677(1608/69) | 400 | 23.85 |
| Bin Zou[478] | 2012 | Sichuan (Zizhong) | 2011 | Rural | | ≥18 | 600 | 61(32/29) | 10.17(11.14/9.26) |
| Huijing He[479] | 2010 | Sichuan(Xichang) | 2008 | Urban | | ≥20 ( 44.34±13.20) | 2037(1038/999) | 149(74/75) | 7.31(7.13/7.51) |
| Yan Pu[480] | 2011 | Sichuan(Panzhihua) | 2010.11 | Rural | | ≥18 | 600 | 54 | 9 |
| Xiaofang Chen[481] | 2004 | Sichuan | 2004 | Urban and Rural | | 18-69 | 1676(775/901) | 10(3/7) | 0.60(0.39/0.78) |
| Yuzhang Zhao[482] | 2011 | Sichuan(Nanchong) | 2010- | Urban | | 58.51±8.26 | 200(92/108) | 24 | 12 |
| Xiaoyu Lan[483] | 2001 | Sichuan(Luzhou) | 1999 | Urban | | ≥40 | 1626(792/843) | 64(31/33) | 3.94(3.91/3.96) |
| Yuxiu Liu[484] | 2002 | Sichuan | 2000.1-2000.12 | Urban | | ≥20 | 6387(3668/2719) | 213(110/103) | 3.33(3.79/3.00) |
| Fang Qin[485] | 2002 | Sichuan(Chengdu) | 1999.1-2000.4 | Urban | | 18~80 (46.87 ±14.15) | 5179(3060/2119) | 160 | 3.09 |
| Ying Zhang[486] | 1999 | Sichuan | 1999 | Urban | | 20-79 | 336(152/184) | 31(17/14) | 9.23（11.18/7.61) |
| Tianmin He[487] | 1997 | Sichuan | 1995.2 | Urban and Rural | | ≥25 | 1170(493/677) | 20 |  |
| Feifei Shen[488] | 1994 | Sichuan(Chengdu) | 1990- |  | |  | 2457(2189/268) | 49(43/6) | 1.99(1.99/2/23) |
| Qingfu Li[489] | 2003 | Sichuan(Chengdu) | 2000- | Urban | | 30-90 | 2916 | 361 | 12.48 |
| Feng Sun[490] | 2013 | Taiwan | 1997.1-2006.12 | Urban | | 35-74 | 24220 | 1040 | 4.29 |
| Tina H. T. Chiu[491] | 2014 | Taiwan | 2007-2009 |  | |  | 4384(2746/1638) | 257(138/119) | 5.86(5.03/7.26) |
| Chih-Jen Chang[492] | 2000 | Taiwan | 1985.11-1996.6 | Urban and Rural | | ≥20 | 5800 | 447 | 7.7 |
| Hsin-Dean Chen[493] | 1997 | Taiwan | 1994.10-1995.12 |  | | ≥40 | 1013(460/553) | 60 | 5.92 |
| Kow-Tong Chen[494] | 2000 | Taiwan(Shonaun) | 1996-1997 |  | | 50-79 | 1293(540/753) | 182(58/109) | 14.08(10.74/14.48) |
| Xiaoqian Liu[495] | 2012 | Tianjin | 2010- | Urban | | ≥18 | 54832(26245/28587) | 4935(2333/2601) | 9.00(8.89/9.10) |
| Hongyue Wang[496] | 2011 | Tianjin | 2010- | Urban/Rural | | ≥60 | 4120(1959/2161) | 677(279/398) | 16.43(14.24/18.42) |
| Gai Chang[497] | 2011 | Tianjin | 2006 |  | | ≥15 | 2886(1367/1519) | 235(119/116) | 8.14(8.71/7.64) |
| Hongmei Liu[498] | 2009 | Tianjin | 2007.11-2007.12 | Urban | | >18 | 11317 | 831 | 7.34 |
| Dan Qian[499] | 2009 | Tianjin | 2008.3-2008.4 | Urban | | ≥50 | 3572 (1568/2004) | 629(263/366) | 17.6(16.8/18.3) |
| Huiguang Tian[500] | 2009 | Tianjin | 2004 | Rural | | ≥35 | 769792(364781/405011) | 73130(30277/42853) | 9.5(8.3/10.5) |
| Xinle Geng[501] | 2009 | Tianjin | 2005.6-9 | Urban and Rural | | 15-74 | 21454(9986/10755) | 1259(1042/1381) | 6.07(5.12/6.95) |
| Wei Li[502] | 2009 | Tianjin | 2006 | Urban and Rural | | ≥15 | 2885(1367/1518) | 235(119/116) | 8.1(8.7/7.64) |
| Yi Yang[503] | 2007 | Tianjin | 2002 | Rural | | ≥18 | 456 | 44 | 9.65 |
| Xiukun Zhang[504] | 2001 | Tianjin(Hebei) | 2000- | Urban | |  | 23970(11550/12420) | 282(99/183) | 11.76(8.57/14.73) |
| Wenxiu Yang[505] | 1999 | Tianjin | 1997 | Urban and Rural | | ≥15 | 35019(17590/17429) | 185 | 0.528 |
| Zhijing Wang[506] | 1999 | Tianjin | 1991-1992 | Urban | | ≥30 | 30921(14365/16556) | 718(293/425) | 2.32(2.04/2.57) |
| Yu Zhang[507] | 2006 | Tianjin | 2004.08--2004.12 | Rural | | ≥15 | 2643859(1260236/1383623) | 229223 (99433/129369) | 8.67(7.89/9.35) |
| Yan Liu[508] | 2010 | Xinjiang | 2009 | Urban | | 24-83 (53.5) | 1286 (898/388) | 78 | 6.06 |
| Rexidanmu.wusiman[509] | 2012 | Xinjiang (Kezhou) | 2009.1-2012.1 | Urban | | 52±7.23 | 291 (168/123) | 19 | 6.53 |
|  |  |  |  |  | | 51±6.87 | 277 (141/136) | 78 | 28.16 |
| Dilare.Adi[510] | 2014 | Xinjiang (Kelamayi) | 2007.10-2010.3 | Urban | | >35 |  |  | 7.5 (9.5/5.8) |
| Leiseng Chen[511] | 2010 | Xinjiang(Kelamayi) | 2007.9-2007.12 | Urban and Rural | | ≥60 | 240 | 55 | 22.92 |
| Shenli Wu[512] | 2012 | Xinjiang(Kelamayi) | 2011.4-5 | Urban | | 40-75(53.39±8.6) | 1214 | 403 | 33.19 |
|  |  |  |  |  | | 40-75(53.94±8.65) | 777 | 335 | 43.11 |
| Yuxia He[513] | 2010 | Xinjiang (Kelamayi) | 2009.6-2009.9 | Urban | | ≥60 | 688 (280/408) | 22(8/14) | 3.2 (2.4/3.4) |
| Zhen Liu[514] | 2014 | Xinjiang (Moyu) | 2007.3-2008.4 | Rural | | 40-65 (51.07±7.05) | (0/274) | (0/16) | (0/5.84) |
| Haimei Yang[515] | 2014 | Xinjiang (Kuitun) | 2013.3-2013.5 | Urban | | ≥18 | 1822 (875/947) | 95 (42/53) | 5.2 (4.8/5.6) |
| Taiping Yin[516] | 2014 | Xinjiang | 1998 | Urban and Rural | |  | 13559 | 100 | 0.74 (0.74/0.73) |
|  |  |  | 2004 |  | |  | 15957 | 270 | 1.69 (1.53/1.86) |
|  |  |  | 2010 |  | |  | 14514 | 396 | 2.73 (2.60/2.86) |
| Yingqin Li[517] | 2013 | Xinjiang (Aksu) | 2012.2-2012.3 | Rural | | 18-79 | 1087 (491/596) | 55 (24/31) | 5.06 (4.89/5.02) |
| Tao Jiang[518] | 2013 | Xinjiang (Shawan) | 2012.6.11-2012.7.6 | Urban | | ≥50 | 201 (90/111) | 28 | 13.93 |
| Weiping Luo[519] | 2013 | Xinjiang | 2010 | Urban and Rural | | ≥18 | 2999 | 368 | 12.28 |
| Gaofeng Sun[520] | 2013 | Xinjiang (Urumqi) | 2010.5 | Urban | | (62.71±11.80) | 505 (225/280) | 93 (43/50) | 18.42 (19.11/17.68) |
| Xiaoqin Tian[521] | 2006 | Xinjiang | 2007.9-2007.12 | Urban | | 20-74 | 3293(1346/1947) | 261 | 7.93 |
| Anniwaer·abulizi [522] | 2011 | Xinjiang(hetian) | 2010.6-2010.7 | Rural | |  | 4619(1949/2670) | 262(117/145) | 5.67(6/5.43) |
| Peng Zhou[523] | 2006 | Xinjiang (Yili) | 2003.4-2003.10 | Urban and Rural | |  | 2260(1118/1142) | 127(77/50) | 5.62 (6.89/4.38) |
| Yicun Tao[524] | 2008 | Xinjiang | 2000- | Urban and Rural | | 30-80 | 1571 | 128 | 8.17(8.42/7.70) |
| Jianguo Sun[525] | 2007 | Xinjiang(Talimu) | 2005.4-2006.4 | Urban and Rural | | 39.381±8.456 | 4917(3442/1475) | 425(264/161) | 8.64(7.66/10.91) |
| Yining Yang[526] | 2012 | Xinjiang | 2007.10-2010.3 |  | |  | 14122 | 1896 |  |
| Yuqiong Huang[527] | 2009 | Xinjiang | 2008 | Urban | | ≥30 | 5188(2713/2475) | 513(291/222) | 9.8(10.7/8.9) |
| Lianqun Su[528] | 2009 | Xinjiang | 2000- | Urban | | 30-81 | 609(259/350) | 75(29/46) | 12.31 |
| Mayinuer[529] | 2005 | Xingjiang(Urumqi) | 2000.8-2001.5 | Urban | | ≥25 | 725(584/141) | 46 | 6.3(6.7/5.0) |
| Xing Yi[530] | 2005 | Xinjiang(Urumqi) | 2002.05-2002.09 | Urban | | 26-86 | 3075(1843/1232) | 292(188/104) | 9.50(10.20/8.44) |
| Zhongxing Xu[531] | 2002 | Xinjiang(Moyu) | 2000- | Urban | | ≥30 | 6000(3250/2750) | 241 | 4.02 |
| Junru Xu[532] | 1992 | Xinjiang(Urumqi) | 1989.9-10 |  | | 63.4 | 861(440/421) | 66(48/18) | 7.67(10.9/4.27) |
| Shengli Wu[533] | 2008 | Xinjiang(Kelamayi) | 2003.03-2003.12 | Rural | | 20-75 | 1536 | 150 | 9.77 |
| Ji De[534] | 2008 | Xizang(Lasa) | 2006 | Urban | | 30-70 | 370(138/232) | 28(27/1) | 7.5(3.6/9.7) |
| Xuegeng Shen[535] | 2012 | Yunnan (Anning) | 2010- | Urban-worker | | 20-59 | 612 | 8 | 1.3 |
|  |  |  |  | Urban-civil servant | | | 598 | 49 | 3.2 |
| Rong He[536] | 2014 | Yunnan (Kunming) | 2011 | Rural | | ≥18 | 4595 (2167/2428) | 286 (122/164) | 6.2 (5.6/6.8) |
| Baokun Peng[537] | 2013 | Yunnan (Kunming) | 2010- | Urban and Rural | | ≥45 | 45163 (19262/25901) | 1551 (664/877) | 3.43 (3.45/3.42) |
| Fang Yan[538] | 2012 | Yunnan(Kunming) | 2009.1-2009.4 |  | | ≥20 | 5195 | 299 | 5.76 |
| Heqin Gong[539] | 2010 | Yunnan(Kuiming) | 2008.1-4 | Urban and Rural | | ≥20 | 5191(1889/3302) | 295(117/178) | 5.7(6.2/5.4) |
| Guangqiong Cao[540] | 2011 | Yunnan(Kunming) | 2008 | Urban | | 55-85 | 2732 | 143 | 5.23 |
| Maorong Zhang[541] | 2008 | Yunnan(Kunming) | 2008 | Rural | | 20-74 | 3440(1101/2339) | 138(50/88) | 4.01(4.54/3.76) |
| Zhao Yang[542] | 2004 | Yunnan(Kunming) | 2003 | Urban | | ≥20 | 5626(2401/3225) | 152(52/100) | 2.70(2.17/3.10) |
| Qingfu Li[489] | 2003 | Yunnan(Kunming) | 2000- | Urban | | 30-90 | 2785 | 241 | 8.65 |
| Zhankun Shu[543] | 2009 | Yunnan(Shilin) | 2000- | Urban | | ≥45 | 611(309/302) | 43(24/19) | 7.0(7.8/6.3) |
| Liping Yang[544] | 2013 | Yunnan (Ninger) | 2009-2012.6 | Urban and Rural | | ≥35 | 127349 | 1519 | 1.19 |
| Aihua Cao[545] | 2005 | Yunnan(Da Li) | 2002-2003 | Rural | | ≥20 | 5000(2346/2654) | 199 | 3.98 |
| Zhiwen Yin[546] | 1999 | Yunnan(Kunming) | 1990- | Urban and Rural | | 40-49 | 446(235/211) | 14 | 3.14 |
| Diabetes Group[547] | 1990 | Yunnan(kunming) | 1990- |  | |  | 12031(8071/3960) | 165(107/58) | 13.57(13/19/14.32) |
| Jianjun Fang[548] | 2014 | Zhejiang (Jinghua) | 2010- | Urban | | ≥65 | 1230 (540/690) | 204 (83/121) | 16.59 (15.37/17.54) |
| Huimin Xu[549] | 2010 | Zhejiang(Jinhua) | 2009 | Urban | | ＞15 | 1015(483/532) | 37(24/13) | 3.68(5.06/2.61) |
| Weijun Zou[550] | 2014 | Zhejiang (Qingtian) | 2013.12 | Urban | | ≥18 | 450 (226/224) | 32 (15/17) | 7.11 (6.64/7.59) |
| Qiaoyu Ye[551] | 2013 | Zhejiang (Qingtian) | 2010- | Rural | | ≥18 (49.58±16.24) | 1248 (584/664) | 134 (56/78) | 10.74 (9.59/11.75) |
| Jian Xu[552] | 2013 | Zhejiang (Cixi) | 2010.7-2010.12 | Rural | | 18-88 (45.72±15.12) | 1147 (570/577) | 105 (44/61) | 9.15 (7.72/10.57) |
| Liming Ji[553] | 2007 | Zhejiang(Cixi) | 2005.3-2006.10 | Urban and Rural | |  | 868(293/575) | 111(27/84) | 12.79(9.22/14.61) |
| Fenfen Wang[554] | 2013 | Zhejiang (Hangzhou) | 2010- | Urban | | 60-81 (72) | 2650 | 156 | 5.9 |
| Chunrong Hu[555] | 2013 | Zhejiang (Hangzhou) | 2010.7-2010.11 | Rural | | ≥18 (53.22±14.69) | 885 (392-493) | 171 (83/88) | 19.32 (21.17/17.85) |
| Wei Zheng[556] | 2012 | Zhejiang(Hangzhou) | 2010 | Urban | | 74-95 (86.25±3.37) | 726(712/14) | 295 (290/5) | 40.63 (40.73/36.71) |
| Hong Zhou[557] | 2011 | Zhejiang(Hangzhou) | 2008 | Urban and Rural | | 45-90 | 13615(4169/9446) | 773 | 5.7 |
| Huaichu Lu[558] | 2013 | Zhejiang (Ningbo) | 2010- | Urban and Rural | | ≥18 | 4125 (1910/2215) | 296 (68/228) | 7.18 (3.61/10.33) |
| Xing Jiang[559] | 2012 | Zhejiang(Ningbo) | 2011 | Rural | | ≥35(59.0±12.7) | 1019(408/611) | 61(19/42) | 6.0(4.7/6.9) |
| Qingqun Zhu[560] | 2013 | Zhejiang (Chisong) | 2010 | Urban and Rural | | ≥18 | 9600 (3658/5942) | 346 | 3.6 |
| Xinhua Pan[561] | 2013 | Zhejiang (Quzhou) | 2010- | Urban and Rural | | ≥30 | 1730 (650/1080) | 84 (27/57) | 4.86 (4.15/5.28) |
| Xiangdong Xu[562] | 2012 | Zhejiang (Huzhou-Nanxun) | 2007.9-2008.3 | Rural | | 16-91 (51.0±15.5) | 2837 (1121/1716) | 70 (23/47) | 2.5 (2.1/2.7) |
| Huihong Fan[563] | 2011 | Zhejiang (Lishui) | 2009 | Urban | | ≥30 (42.59±11.62) | 8452(5385/3067) | 333(238/95) | 3.8(4.8/2.7) |
| Cuihong Xu[564] | 2010 | Zhejiang(Lishui) | 2006~2007 | Urban | |  | 1343(995/348) | 100(70/30) | 7.5(7.0/8.6) |
| Yunfeng Shen[565] | 2012 | Zhejiang(Wuxing) | 2010.5-8 | Urban and Rural | |  | 1107(513/594) | 95 | 8.58 |
| Haijing Ye[566] | 2012 | Zhejiang(Wen Zhou) | 2008 | Urban | | ≥35 | 4028(1764/2264) | 478(211/267) | 11.87(11.96/11.79) |
| Renyu Chen[567] | 2009 | Zhejiang(Wenzhou) | 2000- | Rural | | ≥35 | 1565(576/989) | 88(36/52) | 5.62(6.25/5.26) |
| Shufeng Jin[568] | 2010 | Zhejiang (Shaoxing) | 2008.6-2008.11 | Urban and Rural | | ≥18 | 6706 (3011/3695) | 132 (73/59) | 1.99 (2.42/1.60) |
| Hanjie Jin[569] | 2011 | Zhejiang | 2010- | Urban | | 20-74 | 1047(508/539) | 74(35/39) | 7.07(6.89/7.24) |
| Wei Feng[570] | 2011 | Zhejiang(fenghua) | 2010.10-2010.11 | Rural | | ≥18 | 600(263/337) | 34(13/21) | 5.67(4.94/6.23) |
| Zhong Ye[571] | 2011 | Zhejiang(Xiangshan) | 2009 | Urban and Rural | | ≥25 | 2313 | 106 | 4.58 |
| Jianyong Zheng[572] | 2011 | Zhejiang(Wenzhou) | 2008 | Urban | | ≥18 | 1872(836/1036) | 174(78/96) | 9.29(9.33/9.27) |
| Lan Wang[573] | 2006 | Zhejiang(Yu Yao) | 2004.9-11 | Urban | | 30-60(45±12.8) | 2388(1552/836) | 168(115/53) | 7.04(7.41/6.34) |
| Lina Zhang[574] | 2006 | Zhejiang(Ning Bo) | 2002.10-2003.1 | Urban | | 35-74 | 6589(2976/3613) | 357(161/196) | 5.44(5.42/5.45) |
| Fen Xu[575] | 2009 | Zhejiang(Linan) | 2008.9-11 | Urban and Rural | | ＞35 | 4246(2030/2216) | 148(59/89) | 3.5(2.9/4) |
| Baoding Huang[576] | 2004 | Zhejiang(Wenling) | 2002 | Urban | | ≥20 | 533(479/54) | 19(19/0) | 3.56(3.97/0) |
| Yuping Pan[577] | 2012 | Zhejiang(Yuhuan) | 2010.7-2010.9 | Rural | | ≥35 (52.9±10.1/52.5±8.5) | 25368(12813/12555) | 1576(742/834) | 6.21(5.79/6.64) |
| Weimin Xu[578] | 2004 | Zhejiang(Hangzhou) | 2002-2003 | Urban and Rural | | ≥15 | 4682 | 215 | 4.59 |
| Yifeng Zhang[579] | 2004 | Zhejiang(Shaoxing) | 2000- | Urban and Rural | | 20-74 | 2103(1026/1077) | 61(33/28) | 2.9(3.22/2.6) |
| Ruying Hu[580] | 2005 | Zhejiang | 2001.10-2002.01 | Urban and Rural | | ≥35 | 7259(3077/4182) | 494(200/294) | 6.81(6.5/7.03) |
| Xu Jing[581] | 2002 | Zhejiang(Quzhou) | 2000.11 | Urban and Rural | | (39.59) | 3035(1621/1414) | 80 | 2.64 |
| Guozhang Xu[582] | 1999 | Zhejiang(Ningbo) | 1996-1998 | Urban and Rural | | 20-74 | 2696(1088/1608) | 68(33/35) | 2.52(3.03/2.18) |
| Yunmiao Mei[583] | 1999 | Zhejiang(Yuyao) | 1990- | Urban and Rural | | ≥20 | 2023(866/1157) | 35(19/16) | 1.73(2.19/1.38) |
| Chunhong Fan[584] | 2008 | Zhejiang | 2000- | Urban/Rural | | ≥35 | 6902(2620/4282) | 400 (162/240) | 5.8(6.2/5.6) |
| Diabetes Group[585] | 1998 | Zhejiang | 1994.11-1995.3 | Urban and Rural | | ≥25 | 7949(4467/3482) | 166(119/47) | 2.75(3.6/1.72) |
| Diabetes Group[586] | 1983 | zhejiang | 1974-1976 | Urban | | ≥20 | 17582 | 220 | 1.25 |
|  |  | hangzhou | 1974-1976 |  | |  | 11757 | 169 | 1.44 |
|  |  | fenghua | 1974-1976 |  | |  | 5825 | 51 | 0.88 |
| Jinyun Zhao[587] | 2008 | Zhejiang | 2007.01.01-2007.11.30 | Urban | | 70-97(78.52±5.00) | 3302(2511/791) | 564 | 17.08 |
| Yu Xu[588] | 2013 | China | 2010 | Urban and Rural | | ≥18 | 98658(45143/53515) | 10951(5417/5887) | 11.1(12.1/11.0) |
| Yuna He[589] | 2008 | China | 2002 | Urban and Rural | | ≥18 | 50905(23980/26925) | 1323.53 | 2.6（2.54/2.66） |
| Shuqian Liu[590] | 2011 | China | 2002 | Urban and Rural | | ≥20 | 47729 | 1288.683 | 2.7(2.5/2.8) |
| Wei Liu[591] | 2014 | China(Northwest) | 2009.1-2011.2 | Rural | | ≥20 (45.79±14.65) | 660(288/372) | 33 |  |
| F.Bragg[592] | 2013 | 10 areas in China | 2004-2008 |  | | 30-79 | 512891 (210222/302669) | 14361 | 2.8(2.6/2.8) |
| Jun Liu[593] | 2013 | China | 2009.6-2009.12 |  | | 58.5 ±10.4 | 4942(2466/2476) | 1202(604/598) | 24.3(24.5/24.2) |
| Dongsheng Hu[594] | 2009 | China | 2000–2001 | Urban and Rural | | 35-74 | 15236(7368/7868) | 986(489/497) | 6.5(5.24/6.8) |
| Xiaoren Pan[595] | 1997 | China | 1994 | Urban and Rural | | 25-64 | 213515 | 5338 | 2.5 |
|  |  |  | 1980 |  | | ≥30 | 107954 | 972 | 0.9 |
|  |  |  | 1986 |  | | 25-64 | 109629 | 1140 | 1.04 |
| Yangfeng Wu[596] | 2005 | China | 1993-1994 | Urban/Rural | | 35-59 | 8642 | 328 | 3.8 |
|  |  |  | 1998 |  | | 35-59 | 13643(6493/7150) | 588(285/303) | 4.3(4.4/4.2) |
| Liangqing Zhang[597] | 2002 | China | 2000- | Urban | | ≥35 | 7644 | 894 | 11.7 |
| Diabetes Research Cooperation Group of National[598] | 2002 | China | 1997.8~1998.8 | Urban and Rural | | ≥40 | 29558(13402/16156) | 1675(722/953) | 5.7(5.4/5.9) |
|  |  | Beijing |  |  | |  | 2210(864/1346) | 256(84/172) | 11.6(9.7/12.8) |
|  |  | Huhehaote |  |  | |  | 2424(1033/1391) | 159(75/84) | 6.6(7.3/6.0) |
|  |  | Xilinhaote |  |  | |  | 2821(1234/1587) | 99(40/59) | 3.5(3.2/3.7) |
|  |  | Dalian |  |  | |  | 1671(742/929) | 153(64/89) | 9.2(8.6/9.6) |
|  |  | Haerbin |  |  | |  | 1903(1221/682) | 65(44/21) | 3.4(3.6/3.1) |
|  |  | Shanghai |  |  | |  | 2973(1219/1754) | 204(73/131) | 6.9(6.0/7.5) |
|  |  | Zhengzhou |  |  | |  | 2493(1053/1440) | 112(43/69) | 4.5(4.1/4.8) |
|  |  | Changsha |  |  | |  | 2491(1114/1377) | 123(52/71) | 4.9(4.7/5.0) |
|  |  | Chengdu |  |  | |  | 3233(1465/1768) | 207(103/104) | 6.4(7.0/5.9) |
|  |  | Zunyi |  |  | |  | 2176(941/1235) | 115(45/70) | 5.3(4.8/5.7) |
|  |  | Xi'an |  |  | |  | 2593(1173/1420) | 79(46/33) | 3.1(3.9/2.3) |
|  |  | Lanzhou |  |  | |  | 2570(1343/1227) | 103(53/50) | 4.0(4.0/4.1) |
| Wenying Yang[599] | 2002 | China | 1994 | Urban and Rural | | ≥25 | 223000 | 6266 | 2.81 |
| Hongbing Shen[600] | 1998 | China | 1988-1995 |  | |  | 648144 | 7235 | 1.116 |
|  |  | Shanghai | 1980 |  | |  | 100000 | 1012 | 1.012 |
|  |  | China | 1980-1981 |  | |  | 304537 | 1855 | 0.609 |
|  |  | Beijing | 1982 |  | |  | 25640 | 215 | 0.839 |
|  |  | Sichuan | 1982 |  | |  | 12966 | 63 | 0.486 |
|  |  | Daqing | 1987 |  | |  | 108660 | 815 | 0.75 |
|  |  | Beijing | 1993 |  | |  | 29859 | 953 | 3.19 |
|  |  | Liangning | 1994 |  | |  | 3004 | 62 | 2.06 |
|  |  | Beijing | 1994 |  | |  | 20682 | 711 | 3.44 |
|  |  | China | 1996-1997 |  | |  | 42796 | 1549 | 3.62 |
| Diabetes Research Cooperation Group of National[601] | 1997 | China | 1994 |  | | 25-64 | 213515 | 4864 | 2.28 |
| Nanzi Xie[602] | 1996 | Shanghai and Heilongjiang(Qiqihaer) | 1990- | Urban | |  | 4081 | 195 | 4.78 |
| Hongding Xiang[603] | 1993 | shānxi(qizhou)/beijng/liaoning | 1989-1990 |  | |  | 44747(27938/16809) | 1181(808/373) | 2.02(2.16/1.66) |
| Dongsheng Hu[604] | 2008 | national | 2000-2001 |  | | 35-74 | 15236(7368/7868) | 986(489/497) |  |
